# Discovery and biocatalytic characterization of opine dehydrogenases by metagenome mining

**DOI:** 10.1007/s00253-023-12871-z

**Published:** 2024-01-13

**Authors:** András Telek, Zsófia Molnár, Kristóf Takács, Bálint Varga, Vince Grolmusz, Gábor Tasnádi, Beáta G. Vértessy

**Affiliations:** 1 Department of Applied Biotechnology, Budapest University of Technology and Economics, Budapest, Hungary; 2 Servier Research Institute of Medicinal Chemistry, Budapest, Hungary; 3 Institute of Molecular Life Sciences, Research Centre for Natural Sciences, HUN-REN, Budapest, Hungary; 4 Department of Organic Chemistry and Technology, Budapest University of Technology and Economics, Budapest, Hungary; 5 PIT Bioinformatics Group, Institute of Mathematics, Eötvös University, Budapest, Hungary

**Keywords:** Opine dehydrogenases, Reductive amination, Biocatalysis, Metagenome mining

## Abstract

**Abstract:**

Enzymatic processes play an increasing role in synthetic organic chemistry which requires the access to a broad and diverse set of enzymes. Metagenome mining is a valuable and efficient way to discover novel enzymes with unique properties for biotechnological applications. Here, we report the discovery and biocatalytic characterization of six novel metagenomic opine dehydrogenases from a hot spring environment (mODHs) (EC 1.5.1.X). These enzymes catalyze the asymmetric reductive amination between an amino acid and a keto acid resulting in opines which have defined biochemical roles and represent promising building blocks for pharmaceutical applications. The newly identified enzymes exhibit unique substrate specificity and higher thermostability compared to known examples. The feature that they preferably utilize negatively charged polar amino acids is so far unprecedented for opine dehydrogenases. We have identified two spatially correlated positions in their active sites that govern this substrate specificity and demonstrated a switch of substrate preference by site-directed mutagenesis. While they still suffer from a relatively narrow substrate scope, their enhanced thermostability and the orthogonality of their substrate preference make them a valuable addition to the toolbox of enzymes for reductive aminations. Importantly, enzymatic reductive aminations with highly polar amines are very rare in the literature. Thus, the preparative-scale enzymatic production, purification, and characterization of three highly functionalized chiral secondary amines lend a special significance to our work in filling this gap.

**Key points:**

*• Six new opine dehydrogenases have been discovered from a hot spring metagenome*

*• The newly identified enzymes display a unique substrate scope*

*• Substrate specificity is governed by two correlated active-site residues*

**Supplementary Information:**

The online version contains supplementary material available at 10.1007/s00253-023-12871-z.

## Introduction

Imine reducing enzymes produce a chiral amine often with excellent stereoselectivity. Therefore, there is a constant interest towards them in the pharmaceutical industry. This interest is driven by the high number of active pharmaceutical ingredients (APIs) bearing a chiral amine moiety as illustrated among the FDA-approved small molecule drugs between 2015 and 2020 (Bhutani et al. 2021). Key chiral amine intermediates of these APIs are often produced enzymatically using transaminases (TAs) (Gomm and O’Reilly 2018), amine dehydrogenases (AmDHs), imine reductases (IREDs), or monoamine oxidases (MAOs) (Sharma et al. 2017; Cosgrove et al. 2018; Patil et al. 2018). These enzymes result in chiral primary amines (TAs, AmDHs), perform asymmetric reduction of preformed imines (IREDs), or stereoselectively oxidize cyclic amines to imines (MAOs). Recently, a subclass of IREDs was found to catalyze reductive amination of carbonyls with small primary amines in close to stoichiometric ratio (Aleku et al. 2017); thus, they were termed reductive aminases (RedAms). The applications of RedAms are expanding ever since (Schober et al. 2019), but their substrate scope is still limited to aliphatic or smaller aromatic amines, while reactions with more functionalized polar amines are lagging behind.

Opine dehydrogenases (ODHs) represent a unique and yet largely underexplored class in this rapidly expanding landscape of biocatalysts for reductive amination (Telek et al. 2023). They catalyze the coupling of α-ketoacids with α-amino acids yielding a variety of opine products. These molecules have diverse structures and physiological roles, and different organisms evolved specialized ODHs for their synthesis (Kato and Asano 2002; Harcet et al. 2013; McFarlane and Lamb 2020; Matveeva and Otten 2021). Despite the large evolutionary distance and low apparent sequence similarity among these ODHs, they share general structural and catalytic features (Sharma et al. 2017), while their substrate and cofactor preference can vary significantly. The fact that they are able to perform reductive amination with close to stoichiometric ketoacid/amino acid ratio makes them promising prospects to be utilized as industrial biocatalysts. Considering that their natural substrates are chemically very different from the typical imine reductase or reductive aminase substrates, they can fill in a valuable complementary role next to those classes. Highly functionalized polar secondary amines (such as opine derivatives) are potential building blocks of bioactive compounds such as peptidomimetics (Li Petri et al. 2022). Their stereoselective synthesis by ODHs can be easily envisaged. However, this potential of ODHs is largely unexploited probably due to the significant difference between their native substrates and the potential target substrates (Ducrot et al. 2021) as well as their zwitterionic and highly polar nature. The only example of their utilization as biocatalysts is the ODH from *Arthrobacter* sp. 1C (*Ar*ODH), which was discovered by Asano et al. (1989) and used for the synthesis of secondary amine dicarboxylic acids with apolar sidechains (Kato et al. 1996). A decade later, Codexis engineered this enzyme to accept substrates resembling the classic imine reductase substrates like cyclohexanone or *n*-butylamine. This work—that was only disclosed in a patent (Chen et al. 2013)—also shows that ODHs have to undergo significant engineering to accept substrates largely deviating from their natural ones. However, protein engineering towards structurally similar analogues with orthogonally protected or masked functional groups might be less exhaustive. Isolation of such derivatives could also be more straightforward enabling more versatility for further functionalization. There are a few other well-characterized enzymes among ODHs such as the octopine dehydrogenase from *Pecten maximus* (*Pm*OcDH) (Smits et al. 2008) or metallophore biosynthetic enzymes from *Staphylococcus aureus* (*Sa*ODH), *Pseudomonas aeruginosa* (*Pa*ODH), and *Yersinia pestis* (*Yp*ODH) (McFarlane et al. 2018). These all have different substrate preferences; *Pm*OcDH is specific for pyruvate and l-arginine, while the enzymes from pathogens use exotic nicotianamine-like amino acid derivatives and pyruvate or α-ketoglutarate. We may add that this chemical diversity has not yet been exploited fully for biocatalysis. In addition, there are a lot of sequences in public databases annotated as opine dehydrogenases, but the information on their activity and substrate preferences is lacking; therefore, the above-mentioned diversity is probably even greater. Metagenome search is a method that is capable of sampling a diversity while simultaneously selecting for advantageous properties (Robinson et al. 2021). It involves the interrogation of the whole genetic information extracted from a certain environment. Today, we have the technology of finding genes and enzymes in such metagenomic samples, without identifying the—possibly unknown—host organisms (Kerepesi et al. 2014, 2015; Kerepesi and Grolmusz 2016a, b, 2017). This allows access to potential biocatalysts from previously uncharacterized species that could exhibit altered substrate specificity, increased thermotolerance or catalytic activity. This method was already applied to find novel enzymes for chiral amine synthesis such as transaminases (Baud et al. 2017; Leipold et al. 2019) or amine dehydrogenases (Caparco et al. 2020). For opine dehydrogenases, a recent paper describes one novel metagenome-derived alanopine dehydrogenase (Kaličanin et al. 2023), but no further enzymes are reported to date.

In our study, we set out to expand the class of ODHs with new enzymes with useful properties for industrial biocatalysis. We applied metagenome mining to identify novel ODHs from extreme environments. The catalytic activity and substrate preference of the newly discovered enzymes were investigated under conditions relevant for biocatalytic applications. Their unique structural and sequential features were thoroughly analyzed to aid future engineering of these enzymes towards industrial applications with specific substrates.

## Materials and methods

All reagents and solvents, amino acids, and ketoacids were commercially available and used without further purification.

### HPLC methods

Analytical HPLC measurements were performed on Agilent Technologies 1260 LC system equipped with a DAD detector using Gemini® 3 µm NX-C18, 50 mm × 3.00 mm i.d. 110 Å column, and 5 mM aqueous NH_4_HCO_3_ solution and MeCN as eluents in gradient mode. Analytical LC–MS was performed on an Agilent Technologies 1200 LC system equipped with Agilent 6140 quadrupole MS, operating in positive or negative ion electrospray ionization mode (molecular weight scan range was 100 to 1350 m/z) with parallel UV detection using Gemini® 3 µm NX-C18, 50 mm × 3.00 mm i.d. 110 Å column, and 5 mM aqueous NH_4_HCO_3_ solution and MeCN as eluents in gradient mode. Purifications were carried out with Teledyne Isco preparative HPLC using Gemini® 5 µm NX-C18, 250 mm × 50 mm i.d. 110 Å column, and 5 mM aqueous NH_4_HCO_3_ solution and MeCN as eluents in gradient mode.

### NMR measurements

^1^H NMR and ^13^C NMR spectra were recorded on a Bruker Avance Ultrashield 400 (100 MHz ^13^C) instrument with Bruker Prodigy Cryo Probe and were internally referenced to residual protium solvent signals (note: D_2_O referenced at 4.70 ppm). Samples (5–10 mg) were dissolved in 0.5 mL D_2_O. In the case of Fmoc-**2a** products, 4 µL trifluoroacetic acid (TFA) was added for complete dissolution. Data for ^1^H NMR are reported as follows: chemical shift (*δ* ppm), multiplicity (*s* = singlet, *d* = doublet, dd = doublet of a doublet, *t* = triplet, dt = doublet of a triplet, *m* = multiplet), coupling constant (Hz), and integration. Fmoc-derivatized products form amide rotamers appearing as doubled signals which is indicated by the two *δ* (ppm) values separated by a slash, e.g., 8.38/8.31 (d/d, *J* = 1.3/1.4 Hz, 1 H). Assignments of protons are listed on the spectra (Figure S7-S14). Data for ^13^C NMR are reported in terms of chemical shift, and no special nomenclature is used for equivalent carbons.

### High resolution mass spectrometry

HRMS measurements were carried out on an Agilent 6545 Q-TOF mass spectrometer system: mass resolution, 45.000 FWHM @ m/z 2.722 Da; ion source, AJS-ESI; sheath gas temperature, 300 °C; drying gas temperature, 300 °C; ionizing voltage, 2500 V; and nozzle voltage, 1000 V.

### Metagenome search

The method is based on an artificial intelligence tool, the hidden Markov models (HMMs) (Yoon 2009), described in detail by Szalkai and Grolmusz (2019), and is demonstrated on our webserver for smaller metagenomes (< 1 GB) at the address https://metahmm.pitgroup.org.

First, we trained our metagenome search tool on ten different, already described opine dehydrogenase sequences (listed in Table S1). From these sequences, using multiple alignment with Clustal Omega software as a middle step (Sievers et al. 2011), a hidden Markov model was built with the hmmbuild tool (Eddy 2009). Applying the Markov model, we selected tens of thousands of short reads from the target metagenomes applying the hmmsearch tool (Eddy 2009). The target metagenomes were downloaded from the NCBI Short Read Archive, with accession numbers SRR2915707 (deep-sea sediment metagenome), SRR16646004 (tropical soil metagenome), SRR16606022 (hot spring metagenome, Sativali), and SRR16588181 (hot spring metagenome, Tuwa). The hits from the metagenomes were assembled into the longest possible sequences by the MegaHIT assembler (Li et al. 2015), and from these sequences, we selected potential complete genes with start and end codons (Table S2). Nucleotide sequence data reported are available in the Third Party Annotation (TPA) Section of the DDBJ/ENA/GenBank databases under the accession numbers TPA: BK063522-BK063532.

### Sequence analysis and structural modelling of mODHs

Sequence similarity network (SSN) of the new mODHs and the template ODHs was created using the Enzyme Similarity Tool (EFI-EST, https://efi.igb.illinois.edu/efi-est/) (Zallot et al. 2019). Multiple sequence alignments and phylogenetic trees were generated using Clustal Omega (https://www.ebi.ac.uk/Tools/msa/clustalo/). Structural modelling of mODHs was carried out by open source AlphaFold prediction algorithm accessed through ColabFold (Mirdita et al. 2022). Structures were visualized and analyzed in PyMOL. Electrostatic surface potential calculations were performed using the APBS (Adaptive Poisson-Boltzmann Solver, https://www.poissonboltzmann.org/) plugin in PyMOL (https://www.pymol.org/pymol). Analysis of binding pockets was aided by Caver Web v1.2 (https://loschmidt.chemi.muni.cz/caverweb/) (Stourac et al. 2019).

### Enzyme production and purification

Nine selected mODH genes as well as the coding sequence of *Ar*ODH were codon optimized for *E. coli*, synthesized by Genescript and cloned into pET14b vector using *Nde*I and *Bam*HI cloning sites resulting in N-terminal His-tag proteins (for protein and nucleotide sequences see Table S2). Plasmids were transformed into *E.coli* BL21(DE3) chemically competent cells, and 5 mL overnight cultures were grown in Luria–Bertani (LB) medium supplemented with carbenicillin. Then, 4 mL was added to 500 mL fresh LB medium. The culture was grown at 37 °C to OD = 0.5 and induced by addition of 0.5 mM IPTG. The flasks were then incubated in a shaker at 18 °C overnight. The cells were harvested by centrifugation at 4000 rpm, 4 °C for 30 min. The pellets were resuspended in lysis buffer (50 mM Tris pH = 8.5, 300 mM NaCl, 1 mM benzamidine, 2 mM phenylmethylsulfonyl fluoride (PMSF), 1 mM Tris(2-carboxyethyl)phosphine (TCEP), DNase) and lysed by ultrasonication. The suspension was centrifuged at 11,000 rpm, 4 °C for 30 min. The supernatant was loaded onto equilibrated Ni–NTA column and washed with 15 mM imidazole in lysis buffer. The bound protein was eluted with 250 mM imidazole in lysis buffer and then dialyzed in 50 mM Tris pH = 8.5. The resulting solution was aliquoted after addition of 10% glycerol and then stored at − 20 °C until further use. Total protein concentration was determined based on absorption at 280 nm measured on a NanoDrop spectrophotometer. With this method, six mODHs and *Ar*ODH could be purified (see Table S2). Three mODHs were not expressed in soluble form in this experimental setting.

### Site-directed mutagenesis

Mutagenesis experiments were conducted according to the Q5® Site-Directed Mutagenesis Kit Quick Protocol provided by the manufacturer (Table S6). KLD reactions were transformed into XL1Blue chemically competent cells and were plated on LB-TC/CAR agarose plates. Single colonies were picked and used to inoculate 5 mL overnight cultures. From these, plasmids were purified according to the NucleoSpin Plasmid Prep protocol. The purified plasmids were sent to sequencing to verify mutations and then used for protein expression.

### Differential scanning fluorimetry (Thermofluor) measurements

To 1 mg/mL solutions of enzymes (mODHs, *Ar*ODH) was added 1000 × SYPRO Orange dye. Twenty-five-microliter samples were loaded onto 96-well plates in triplicates. Fluorescent signal versus temperature was in a CFX96 real-time PCR detection system (Bio-Rad) with a gradient from 25 to 95 °C (0.5 °C steps).

### Kinetic measurements

Enzyme activities were measured spectrophotometrically at 30 °C in 96-well half-area plates in a Thermo Scientific™ Multiskan SkyHigh Microplate Spectrophotometer (Thermo Fisher Scientific Inc., Waltham, MA, USA). Standard assays were carried out by using 15 mM amino acid and 0.2 mM NAD(P)H in 100 mM sodium phosphate buffer (pH = 8.0). The reaction was started by the addition of 10 mM pyruvate. The decrease in absorbance was monitored at 340 nm. Initial velocities (*v*_0_) were calculated from the linear section of the plots using an NAD(P)H calibration. For the determination of kinetic constants, amino acid concentrations were varied between 1 and 30 mM, while cofactor concentrations were between 0.04 and 0.5 mM. Depending on the specific activities of each enzyme, enzyme concentrations were fixed between 0.015 and 1.5 µM (Tables S9-S12). Kinetic constants were determined from the Michaelis–Menten plots using GraphPad Prism software (Tables S9-S12, Figures S15-S18). Heat stability experiments were carried out by incubating the enzymes at 40, 50, or 60 °C for 1 h before measuring their specific activities.

### Small scale biocatalytic reactions, parameter optimizations

For preliminary activity measurements and reaction optimization, the reaction mixtures (500 µL) contained 62.5 mM d-glucose, 6 U/mL GDH (Codexis CDX-901, 50 U/mg), 0.4 mM NAD^+^/NADP^+^, 25 mM amino acid substrate, and 5 equivalents of sodium pyruvate substrate in Na-phosphate buffer adjusted to pH 7.5 in a 1.5-mL Eppendorf tube. To the blank mix was added 30 µg purified enzyme (mODHs, *Ar*ODH). The reaction mixtures were incubated at 30 °C with shaking at 300 rpm for 24 h. Parameters were optimized on the following ranges: pH 6.0–8.0, temperature 30–50 °C, pyruvate equivalence 1.5–5.0, and enzyme loading 7.5–60 µg/mL. To follow the reactions, 100-µL samples were taken and derivatized based on the protocol from Tassano et al. (2022). Samples were diluted 1:1 with Na-borate buffer (300 mM, pH 9.2), then 200 µL of a solution of Fmoc-Cl (15 mM in ACN) was added, and the samples were shaken for 10 min, 30 °C, and at 1000 rpm. Next, 200 µL solution of amantadine hydrochloride (300 mM in 1:1 H_2_O/ACN) was added. The formed white precipitate was centrifuged 5 min/1500 rpm, and the supernatant was analyzed. Analysis was done on HPLC–MS. The conversion was calculated by measuring the depletion of the amino acid as follows: HPLC area of the Fmoc-amino acid peak in the enzymatic reaction was compared to that of blank reactions carried out under identical conditions without ODH present.

### Substrate screening

On a 1-mL 96-deep-well plate, 500 µL reactions were carried out. The reaction mixtures contained 62.5 mM d-glucose, 6 U/mL GDH (Codexis CDX-901, 50 U/mg), 0.4 mM NADP^+^ (or NAD^+^ for *Ar*ODH), 25 mM amino acid substrate, and 75 mM of ketoacid substrate in Na-phosphate buffer adjusted to pH = 8.0. To the blank mix was added 15 µg purified enzyme (six mODHs, *Ar*ODH). The reaction mixtures were incubated at 40 °C with shaking at 300 rpm for 24 h. Reactions were performed in triplicates. Sample preparation and calculation of conversion were done as described in the previous section, and analysis was carried out on HPLC.

### Preparative scale reactions and purifications

30 mL reaction mixture contained 62.5 mM d-glucose, 6 U/mL GDH (Codexis CDX-901, 50 U/mg), 0.4 mM NADP^+^, and 25 mM amino acid substrate with 3 equivalents of sodium pyruvate in Na-phosphate buffer adjusted to pH 8.0. To the blank mix was added 900 µg purified enzyme (*Ar*ODH or mODH-582). The reaction mixture was stirred at 40 °C for 24 h. After 24 h, the reaction was boiled for 15 min, and then, the precipitate was filtered out. To the filtrate was added 8 equivalents Fmoc-Cl in 60 mL ACN, and the mixture was stirred at 60 °C for 16 h. Afterwards, the pH was adjusted to 7.5, and the mixture was extracted twice with EtOAc. The aqueous phase was concentrated and purified by preparative HPLC.

### (2S)-2-[[(1R)-1-carboxyethyl]-(9H-fluoren-9-ylmethoxycarbonyl)amino]-3-(1H-imidazol-4-yl)propanoic acid (Fmoc-(1R,2S)-2a)

The above protocol performed with mODH-582 afforded Fmoc-(1*R*,2*S*)-**2a** as a white powder (yield = 20%, 51 mg).

^1^H-NMR (400 MHz, D_2_O + TFA): *δ* ppm 8.38/8.31 (d/d, *J* = 1.3/1.4 Hz, 1 H), 7.74–7.26 (*m*, 8 H), 6.81/6.14 (s/s, 1 H), 5.02–4.56 (*m*, 2 H), 4.25/3.81 (dd/dd, *J* = 8.2/8.9, 6.0/6.2 Hz, 1 H), 4.20/4.13 (bs/bs, 1 H), 4.13/3.70 (q/q, *J* = 14.2/14.3, 7.1/7.2 Hz, 1 H), 3.17–1.96 (*m*, 2 H), 0.98/0.47 (d/d, *J* = 7.2/7.1 Hz, 3 H).

^13^C-NMR (100 MHz, D_2_O + TFA): *δ* ppm 174.9, 174.6, 173.2, 172.3, 163.3, 162.9, 162.6, 162.2, 156.3, 155.9, 143.7, 143.6, 143.5, 143.4, 141.4, 141.3, 141.1, 140.9, 132.7, 129.6, 129.0, 127.9, 127.8, 127.7, 127.6, 127.4, 127.3, 127.1, 124.4, 124.3, 124.2, 124.1, 120.5, 120.1, 120.0, 117.6, 116.8, 116.7, 114.7, 111.8, 67.0, 66.7, 59.2, 57.9, 55.9, 55.8, 46.8, 46.6, 24.4, 14.1, 13.7.

HRMS *m/z* ([M + H]^+^) calcd. for C_24_H_24_N_3_O_6_ 450.1660 found 450.1661 (*δ* = 0.22 ppm).

The above protocol performed with *Ar*ODH afforded Fmoc-(1*R*,2*S*)-**2a** as a white powder (yield = 40%, 91 mg).

The analytical data are identical to those obtained for Fmoc-(1*R*,2*S*)-**2a** using mODH-582.

### (2S)-2-[[(1R)-1-carboxyethyl]-(9H-fluoren-9-ylmethoxycarbonyl)amino]butanedioic acid (Fmoc-(1R,2S)-3a)

The above protocol performed with mODH-582 afforded Fmoc-(1*R*,2*S*)-**3a** as a white powder (yield = 24%, 77 mg).

^1^H-NMR (400 MHz, D_2_O): *δ* ppm 7.77–7.26 (*m*, 8 H), 4.76–4.48 (*m*, 2 H), 4.21–4.16 (*m*, 1 H), 4.10/4.00 (dd/dd, *J* = 9.7/8.0, 4.7/6.8 Hz, 1 H), 3.90/3.74 (q/q, *J* = 14.0/14.1, 7.0/7.0 Hz, 1 H), 2.80–2.00 (*m*, 2 H), 1.19/0.73 (d/d, *J* = 7.0/7.0 Hz, 3 H).

^13^C-NMR (100 MHz, D_2_O): *δ* ppm 178.2, 177.6, 177.4, 177.3, 176.8, 175.6, 156.1, 156.1, 143.8, 143.8, 143.5, 143.4, 141.2, 141.1, 141.0, 141.0, 127.9, 127.8, 127.8, 127.8, 127.4, 127.4, 127.3, 127.2, 124.7, 124.5, 124.4, 120.2, 120.1, 120.1, 120.0, 67.0, 66.9, 64.4, 61.6, 61.4, 46.8, 46.8, 37.1, 15.1, 14.9.

HRMS *m/z* ([M + H]^+^) calcd. for C_22_H_22_NO_8_ 428.1340 found 428.1342 (*δ* = 0.47 ppm).

### (2R)-2-[[(1R)-1-carboxyethyl]-(9H-fluoren-9-ylmethoxycarbonyl)amino]-3-sulfo-propanoic acid (Fmoc-(1R,2R)-7a)

The above protocol performed with mODH-582 afforded Fmoc-(1*R*,2*R*)-**7a** as a white powder (yield = 23%, 80 mg). Note that there is no switch in stereoselectivity but the substituent priority according to the CIP convention changed.

^1^H-NMR (400 MHz, D_2_O): *δ* ppm 7.78–7.26 (*m*, 8 H), 4.83–4.54 (*m*, 2 H), 4.17–4.14 (*m*, 1 H), 4.04/3.88 (dd/dd, *J* = 9.3/9.2, 3.3/3.2 Hz, 1 H), 3.81–3.74 (*m*, 1 H), 3.47–2.13 (*m*, 2 H), 1.28/0.83 (d/d, *J* = 7.0/7.0 Hz, 3 H).

^13^C-NMR (100 MHz, D_2_O): *δ* ppm 177.9, 176.7, 175.6, 174.1, 155.9, 155.6, 143.7, 143.7, 143.5, 143.4, 141.3, 141.3, 141.1, 128.0, 127.8, 127.8, 127.4, 127.3, 127.2, 124.6, 124.4, 124.3, 124.2, 120.2, 120.2, 120.1, 120.1, 67.0, 66.9, 63.9, 63.1, 62.8, 62.1, 51.0, 50.8, 46.7, 46.6, 14.6, 14.6.

HRMS *m/z* ([M + NH_4_]^+^) calcd. for C_21_H_25_N_2_O_9_S 481.1275 found 481.1276 (*δ* = 0.21 ppm).

### Synthesis of reference materials Fmoc-(1R,2S)-2a* and Fmoc-(1S,2S)-2a

In a 100-mL round-bottom flask, methanol (40 mL) was added to l-histidine (2 mmol, 310.3 mg) and Na-acetate (4 mmol, 328.1 mg) followed by pyruvic acid (4 mmol, 278.0 µL). Then, NaCNBH_3_ (4 mmol, 4 mL of 1 M solution in THF) was added dropwise at RT. The solution was stirred at RT for 20 h. After histidine appeared absent on LC–MS, 9-fluorenylmethyl chloroformate (Fmoc-Cl) (4 mmol, 1.03 g) was added, and the mixture was stirred at 60 °C. After 3 h, another batch of Fmoc-Cl (2 mmol, 515 mg) was added, and stirring was continued for 4 h at 60 °C and then for 4 days at RT. MeOH was removed under reduced pressure. The residue was partially dissolved in 1 M HCl (8 mL) and extracted with 15 mL EtOAc. Both phases contained the product diastereomers. The aqueous phase was purified on preparative HPLC. The organic phase was evaporated to dryness. The residue was dissolved in acetonitrile (8 mL) and purified on preparative HPLC. Fractions containing the corresponding diastereomers were combined and freeze dried overnight resulting in Fmoc-(1*R*,2*S*)-2a* (6%, 53 mg; * labels its chemical origin) and Fmoc-(1*S*,2*S*)-**2a** (3%, 30 mg).

### (2S)-2-[[(1R)-1-carboxyethyl]-(9H-fluoren-9-ylmethoxycarbonyl)amino]-3-(1H-imidazol-4-yl)propanoic acid (Fmoc-(1R,2S)-2a*)

The obtained analytical data are identical to those of Fmoc-(1*R*,2*S*)-**2a**.

### (2S)-2-[[(1R)-1-carboxyethyl]-(9H-fluoren-9-ylmethoxycarbonyl)amino]-3-(1H-imidazol-4-yl)propanoic acid (Fmoc-(1S,2S)-2a)

^1^H-NMR (400 MHz, D_2_O + TFA): *δ* ppm 8.37/8.27 (bs/d, *J* = 1.2 Hz, 1 H), 7.69–7.21 (*m*, 8 H), 6.88/5.84 (s/s, 1 H), 4.98–4.53 (*m*, 2 H), 4.23/3.81 (dd/dd, *J* = 8.1/10.5, 6.1/5.8 Hz, 1 H), 4.16/4.06 (bs/bs, 1 H), 3.67–3.58 (*m*, 1 H), 3.18–1.81 (*m*, 2 H), 0.94/0.55 (d/d, *J* = 7.0/7.1 Hz, 3 H).

^13^C-NMR (100 MHz, D_2_O + TFA): *δ* ppm 174.4, 171.7, 163.0, 162.6, 156.0, 155.9, 143.8, 143.7, 143.4, 141.5, 141.2, 140.8, 132.9, 132.8, 129.1, 128.5, 128.0, 127.8, 127.7, 127.7, 127.4, 127.3, 127.2, 124.3, 124.2, 120.5, 120.2, 120.0, 117.6, 116.9, 114.7, 111.8, 66.7, 66.6, 60.1, 59.9, 58.6, 56.5, 46.8, 46.6, 24.5, 24.3, 14.1, 13.2.

HRMS *m/z* ([M + H]^+^) calcd. for C_24_H_24_N_3_O_6_ 450.1660 found 450.1660 (*δ* = 0.00 ppm).

## Results

### Metagenome mining of ODHs

**Fig. 1:**
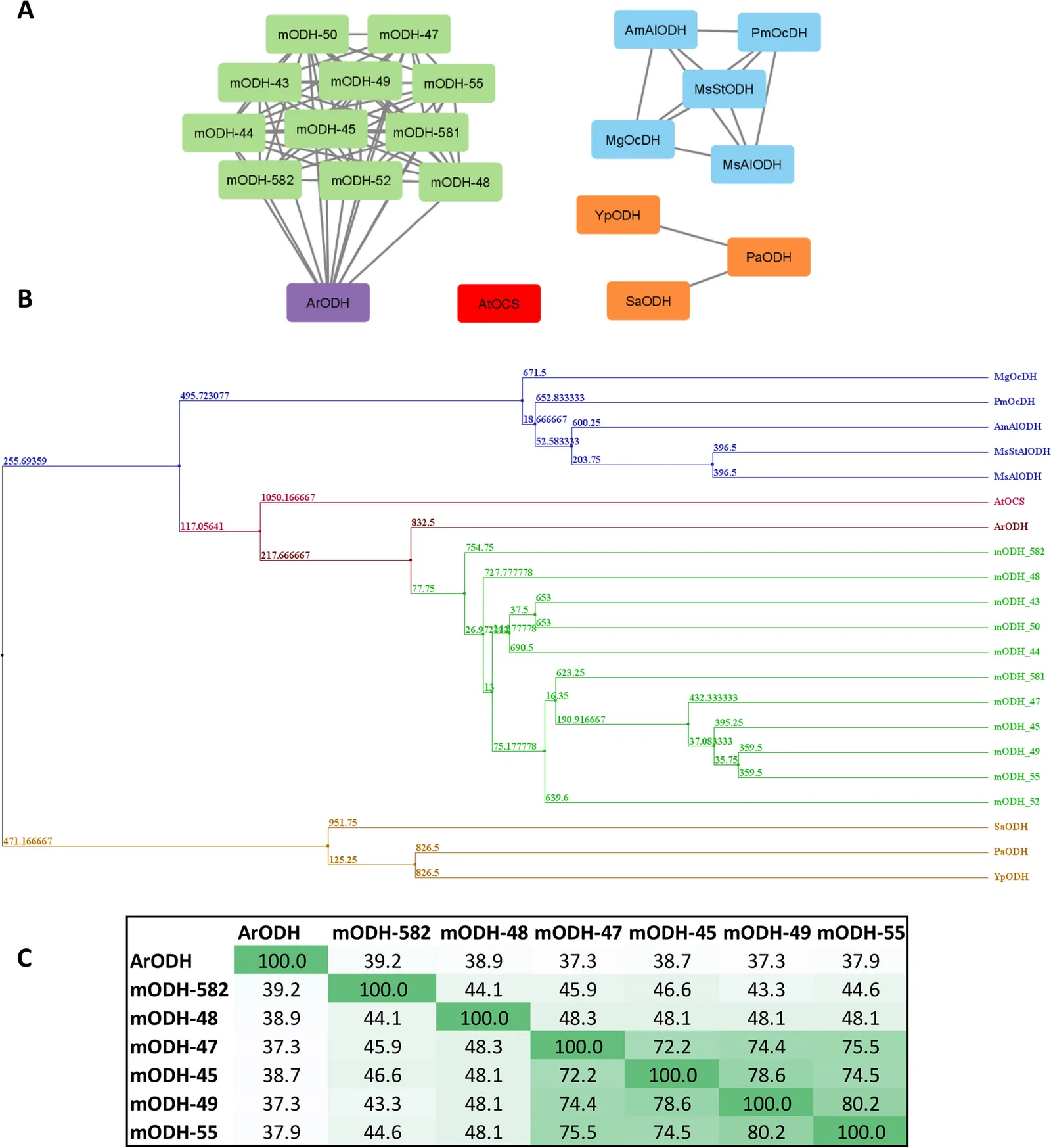
**A** Sequence similarity network (SSN) of ODHs with a connection threshold of 30% sequence identity. **B** Phylogenetic tree of ODHs. In both cases, template sequences used for the metagenome search are colored according to their origin: marine invertebrates (blue), pathogenic bacteria, naturally transgenic plants (red), soil bacteria (purple). New metagenomic ODHs are light green. **C** Sequence identity matrix of six mODHs (that could be purified and tested) and *Ar*ODH

We started with building a database containing ODH sequences that can be used as templates for metagenome search. We mined the literature for enzymes with known primary structure and confirmed activity to avoid falsely annotated sequences. In the end, we have collected 11 sequences from a diverse set of organisms and with wide substrate and cofactor preferences (see Fig. 1 and Table S1). These were used to construct a multiple sequence alignment to train a hidden Markov model (HMM), as described in a recent publication (Szalkai and Grolmusz 2019). In order to find enzymes with preferable properties for industrial biocatalysis, we selected datasets from NCBI Short Read Archive collected from extreme environments (see “Materials and Methods”). The trained HMM was used to extract full-length sequences resembling the general features of ODHs. From the four different datasets (see “Materials and Methods”), only one, the Sativali hot spring (67 °C) (Narsing Rao et al. 2022), metagenome provided new putative ODH genes. From that dataset, 11 sequences were obtained that showed remarkably low sequence identity (< 40%) with all template sequences used while displaying higher degree of similarity among each other (Fig. 1A and B). Interestingly, although the HMM included characteristics from all the 11 diverse ODHs, still, for all of the finally identified mODHs, the closest homologue in the template set is the ODH from *Arthrobacter* sp. 1C (*Ar*ODH). Since *Ar*ODH is the only known example of an ODH used in biocatalysis, we decided to use it as a reference enzyme for the characterization of the metagenomic ODHs (mODHs) as potential new biocatalysts. From the 11 sequences, we have discarded two due to the lack of a consensus sequence required for cofactor binding at the front of these genes (see Table S2). Thus, we have ordered ten synthetic genes (nine mODHs and *Ar*ODH), cloned into pET14b vector, expressed them in *E. coli* and purified them. Three metagenomic enzymes did not exhibit soluble expression in our system, so in the end, six mODHs and *Ar*ODH were obtained with yields between 10 and 70 mg/L culture. Those metagenomic enzymes have close to 40% sequence identity to *Ar*ODH but have varying degree of similarity (~ 45–80%) among each other (Fig. 1C).

### Substrate screen for mODHs

**Scheme 1:**
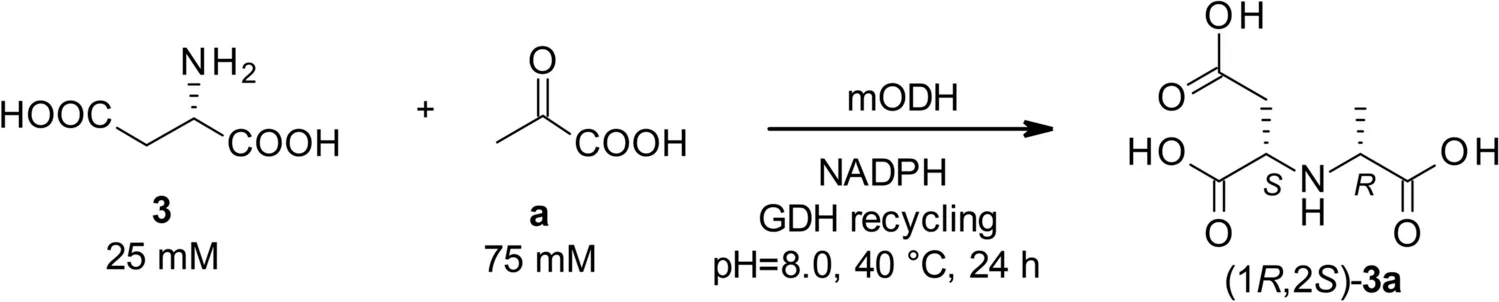
Representative chemical reaction catalyzed by mODHs. Reaction conditions (pH, temperature, pyruvate equivalence, enzyme loading) were optimized to achieve the highest conversions in 24 h. Screening was conducted by varying either the amino acid (labelled with numbers) or the ketoacid substrate (labelled with letters)

As the main aim of our study was to explore and exploit the synthetic utility of the newly discovered enzymes, we have tested their activity in small scale biocatalytic reactions (Scheme 1), where their activity is measured by the apparent conversion to the product. The reactions were followed by HPLC with UV and MS detection. Since most of the used substrates had poor retention on normal phase and weak UV signal, we have applied a derivatization method (Tassano et al. 2022) to follow the consumption of the amino acid substrate (see “Materials and Methods”). The presence of the product could be confirmed based on its MS signal. Preliminary experiments showed that mODHs have negligible activity with the best amino acid substrates of *Ar*ODH, like l-norvaline or l-phenylalanine and only few percent conversion with l-alanine when using NADH, the usual cofactor for *Ar*ODH. In the latter case, the observable product formation prompted us to test NADPH instead of NADH as cofactor, which yielded enhanced conversions (data not shown). Therefore, NADPH was used for the optimization of reaction conditions and substrate screening. Next, after screening a restricted set of natural amino acids including apolar (alanine, leucine, valine, methinonine), polar (serine, threonine, asparagine, glutamine), and charged ones (aspartate, glutamate), we have identified l-aspartate (**3**) to be a preferred substrate for mODHs. Pyruvate (**a**) is often the only ketoacid substrate of ODHs; therefore, we did not consider changing that substrate at this stage. We used the l-aspartate (**3**) and pyruvate (**a**) substrate combination to optimize reaction conditions for extensive substrate screening using two selected best performing enzymes (mODH-45 and mODH-49). The optimum conditions are different from the ones normally used for *Ar*ODH with higher temperature (30 vs. 40 °C) and slightly higher pH (7.5 vs. 8.0) (Kato et al. 1996) (Scheme 1).

**Fig. 2:**
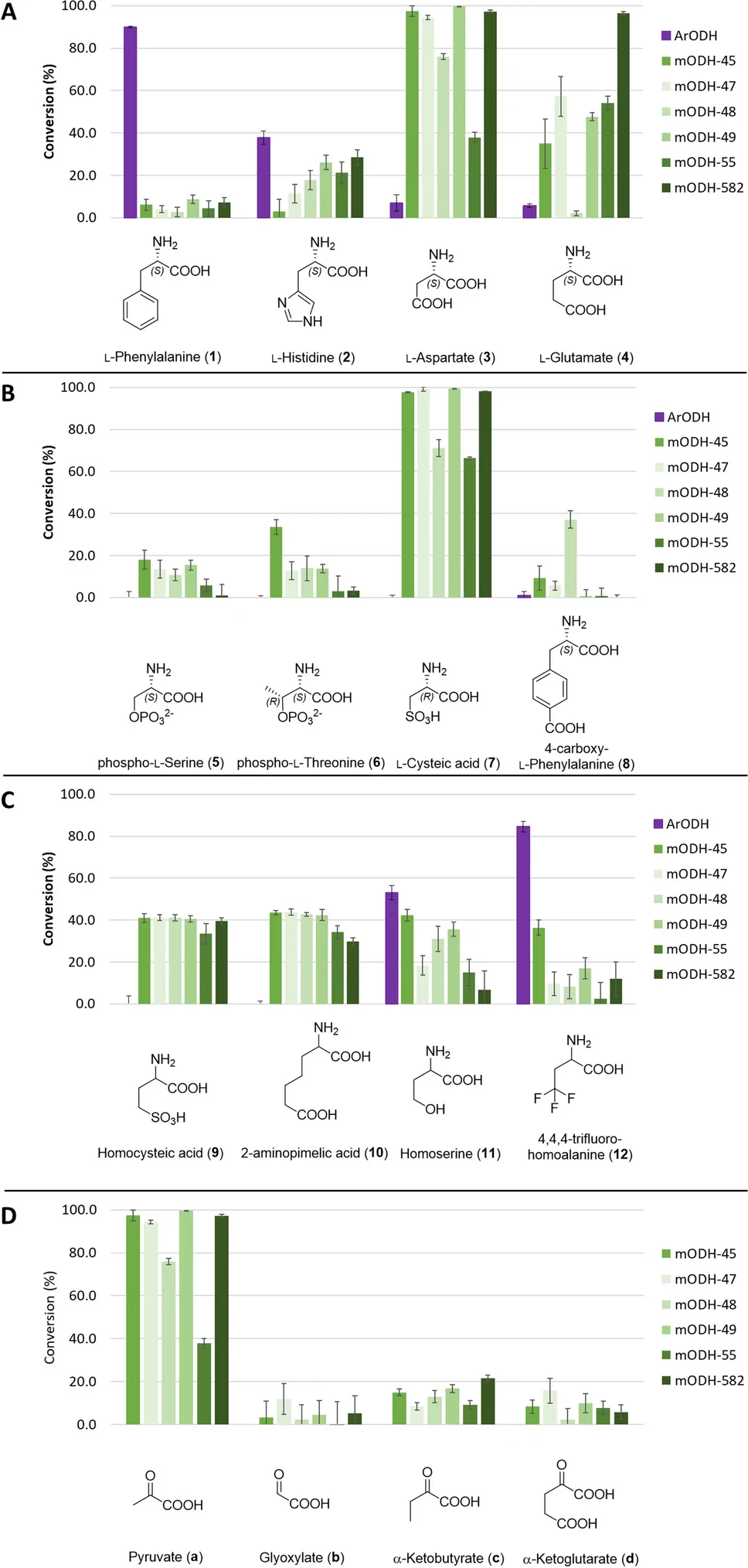
Selected results from the substrate screening of mODHs. (For data for all substrates, see SI.) **A** Canonical amino acid substrates (pyruvate is fixed as ketoacid substrate). **B** Enantiopure non-canonical amino acid substrates (pyruvate is fixed as ketoacid substrate). **C** Racemic non-natural amino acid substrates (pyruvate is fixed as ketoacid substrate). Since these substrates were used as racemates, the maximal conversion is expected to be 50% assuming full enantiomer selectivity towards l-amino acids. **D** Ketoacid substrates (l-aspartate is fixed as amino acid substrate). Conversions were calculated as depletion of the amino acid substrate in a small scale biocatalytic reaction. Each reaction was measured in triplicates, and error bars represent the standard deviation calculated (see Tables S3 and S4)

Using these conditions, we set out to investigate the activity of mODHs on a diverse set of amino acid and ketoacid substrates. Amino acids included 13 canonical (**1–4**, **13–21**) and 13 non-canonical amino acids (**5–12**, **22–26**), while on the ketoacid side, only natural substrates were tested (see Tables S3 and S4). The screening results show that in contrast to *Ar*ODH’s preference for apolar amino acid substrates, mODHs exhibit the highest conversions with polar amino acids, mostly with negatively charged sidechains (Fig. 2). l-Aspartate (**3**) and l-glutamate (**4**) clearly stand out among the natural amino acids with four of six mODHs exhibiting 100% conversion with the former and above 50% with the latter. Exchanging the carboxylate with a sulfonate group (l-cysteic acid (**7**) or homocysteic acid (**9**)) results in similar or even better substrates for mODHs. Notably, phosphorylated amino acids (**5**, **6**) are converted significantly less efficiently, even though mODHs are still superior to *Ar*ODH in these reactions. Aromatic side chains are generally not well tolerated by mODHs; however, l-histidine (**2**) is transformed with conversions comparable to *Ar*ODH, while mODH-48 is also capable of transforming 4-carboxy-l-phenylalanine (**8**) with moderate conversion. Opine dehydrogenases have strong enantiomer specificity towards l-amino acids (Telek et al. 2023). mODHs share this feature as we could demonstrate by comparing conversions with d- and l-aspartate (data not shown). This strong specificity can explain the moderate conversions with racemic amino acids (homocysteic acid (**9**), 2-aminopimelic acid (**10**), homoserine (**11**)) that otherwise proved to be good substrates of mODHs. Among ketoacids, pyruvate (**a**) is clearly preferred above all others. α-Ketobutyrate (**c**) is accepted to some extent, while conversions with glyoxylate (**b**) and α-ketoglutarate (**d**) are negligible (except for mODH-47).

### Kinetic characterization of mODHs

After testing the mODHs in small-scale biocatalytic reactions, we wanted to gain insight into their kinetic properties that govern the above-described apparent catalytic activities. We have performed Michaelis–Menten kinetic studies to assess the amino acid substrate kinetics and determined kinetic constants for NADH/NADPH (Table 1). The mODHs show similar characteristics in their turnover numbers (*k*_cat_); the differences between their catalytic efficiencies can be attributed to their *K*_M_ values which can deviate more than an order of magnitude from each other. The cofactor kinetics corroborate our assumption of NADPH preference for most of mODHs with the exception of mODH-48 which shows similar catalytic efficiency with NADH and mODH-582 which appears to prefer NADH. In the latter case, the catalytic efficiency with NADPH is still comparable to the other mODHs.

**Table 1:** Kinetic parameters of mODHs. The cofactor preference is indicated in the last column as the ratio of catalytic efficiencies (*k*_cat_/*K*_M_) with NADH and NADPH; thus, a value > 1 indicates NADH preference, and a value < 1 indicates NADPH preference

Enzyme	l-Asp			NADH			NADPH			NADH/NADPH
	*K*_M_ (mM)	*k*_cat_ (1/s)	*k*_cat_/*K*_M_ (s^−1^ mM^−1^)	*K*_M_ (µM)	*k*_cat_ (1/s)	*k*_cat_/*K*_M_ (s^−1^ mM^−1^)	*K*_M_ (µM)	*k*_cat_ (1/s)	*k*_cat_/*K*_M_ (s^−1^ mM^−1^)	
mODH-45	21.5 ± 5.3	0.76 ± 0.11	0.04	447 ± 64	0.90 ± 0.08	2.0	118 ± 24	1.00 ± 0.08	8.5	0.24
mODH-47	1.2 ± 0.3	0.11 ± 0.01	0.09	No reaction			93 ± 21	0.33 ± 0.03	3.6	0.00
mODH-48	0.5 ± 0.1	0.70 ± 0.02	1.46	93 ± 15	4.4 ± 0.3	47.0	17 ± 7	0.76 ± 0.04	44.5	1.06
mODH-49	15.0 ± 2.3	0.93 ± 0.07	0.06	1828 ± 423	4.9 ± 1.0	2.7	85 ± 17	1.23 ± 0.09	14.4	0.19
mODH-55	1.5 ± 0.2	1.55 ± 0.05	1.07	442 ± 63	0.50 ± 0.04	1.1	68 ± 13	1.80 ± 0.11	26.5	0.04
mODH-582	24.8 ± 4.6	0.89 ± 0.10	0.04	29 ± 11	18.1 ± 1.4	627	318 ± 112	0.62 ± 0.14	1.9	323.31

### Heat stability of mODHs

The mODH sequences have been identified from the metagenomic data that were collected for microbial communities living under extreme conditions such as in hot springs. It was therefore of interest to decide if their protein stability may reflect adaptation to the extreme conditions. We applied differential scanning fluorimetry (Niesen et al. 2007; Gao et al. 2020) to determine the melting point of these mODH proteins. As also shown in Table 2, all mODHs that we purified showed drastic increase (15–30 °C) in thermal stability as compared to the model *Ar*ODH (see also Figure S2 for raw melting curve data). This result is encouraging for enzyme stability under chemical process conditions.

**Table 2:** Melting temperatures of mODHs measured by differential scanning fluorimetry

Enzyme	Melting temperature (°C)
*Ar*ODH	44.0 ± 0.3
mODH-45	74.0 ± 0.5
mODH-47	60.0 ± 0.3
mODH-48	63.0 ± 0.3
mODH-49	61.0 ± 0.0
mODH-55	71.0 ± 0.3
mODH-582	74.5 ± 0.5

**Fig. 3:**
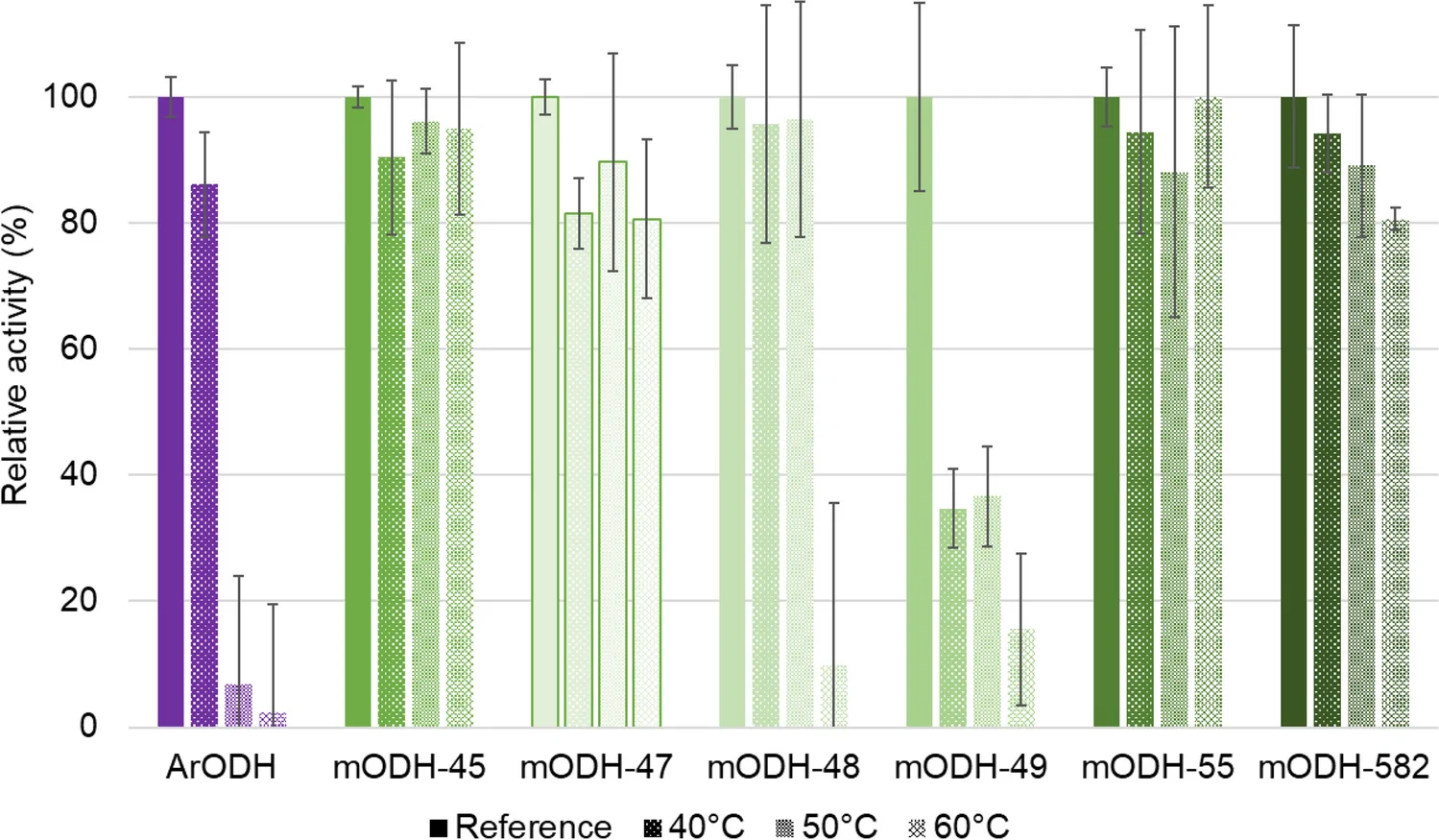
Residual specific activities of ODHs after 1-h incubation at different temperatures

In addition, we have performed heat stability experiments by incubating the enzymes at varying temperatures before measuring their specific activities. From the residual activities, it is clear that while *Ar*ODH loses activity at 50 °C, the metagenomic enzymes show moderate or no loss of activity even at 60 °C (Fig. 3). This result indicates that the metagenomic enzymes derived from a hot spring have not only higher melting temperatures (see above) but they can also retain their activity after exposure to higher temperatures.

### Molecular determinants of substrate preference

**Fig. 4:**
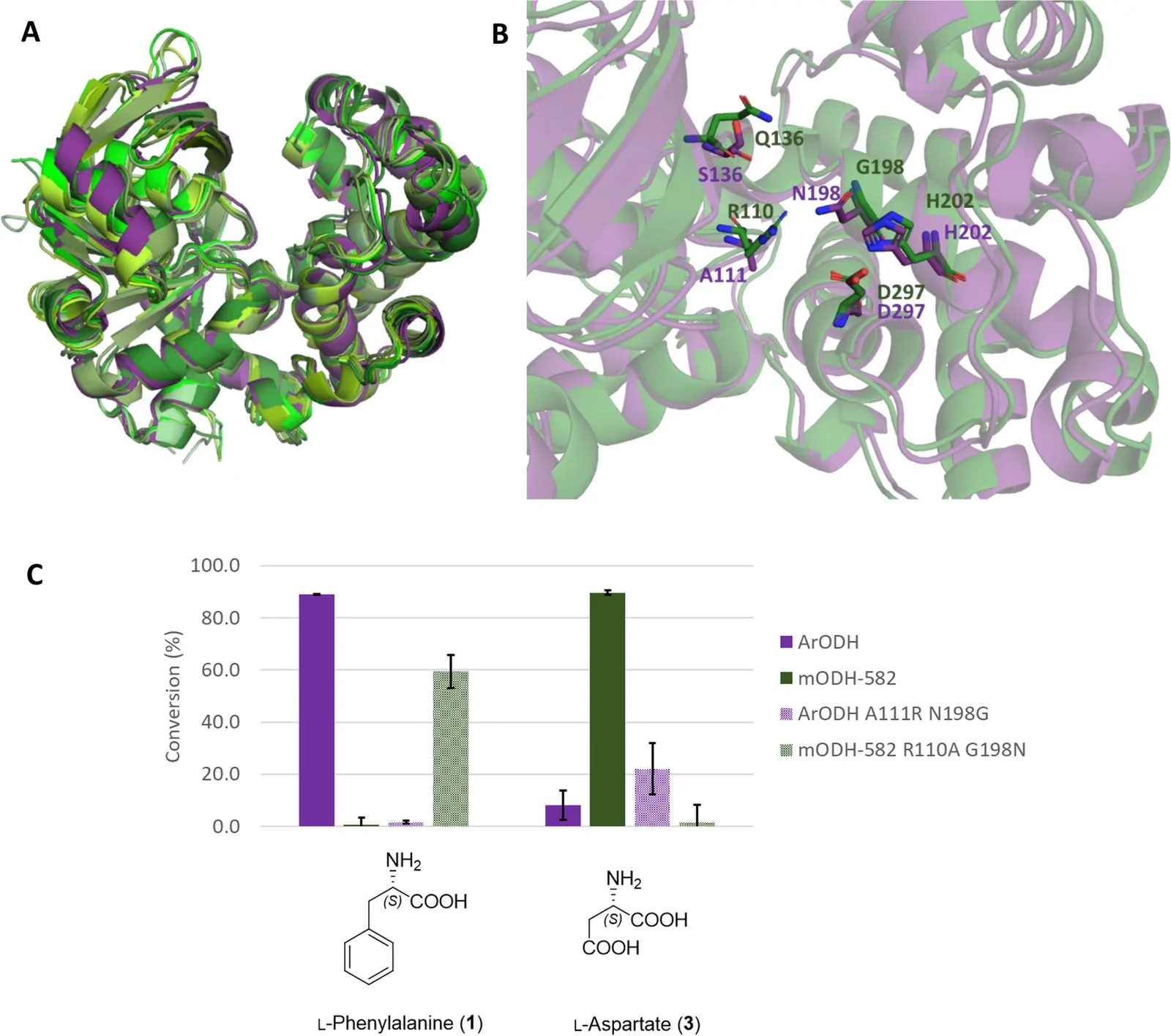
**A** Alignment of AlphaFold models of the six mODHs (greens) with the crystal structure of ArODH (purple, PDB:1BG6). **B** Comparison of the active sites of mODH-582 (green) and *Ar*ODH (purple). The conserved catalytic histidine-aspartate dyad as well as some notably different residues surrounding the active site are highlighted. **C** Switch of substrate preference of mODH-582 and *Ar*ODH as a result of a double point-mutation in their active sites indicated by conversion values with different substrates in small-scale biocatalytic reactions

In order to better understand the factors determining the different amino acid substrate preferences of ODHs, we performed detailed analysis of their sequences and structures. For the latter structures, models predicted by AlphaFold were used. The overall structure of mODHs resembles well other opine dehydrogenases even with very low sequence identity (Fig. 4). The structural family of enzymes sharing this overall fold is named octopine dehydrogenases (Pfam 02317) after the first and most well-characterized enzyme from *Pecten maximus* (*Pm*OcDH) (Smits et al. 2008) and octopine synthases (OCS) from naturally transgenic plants (Hack and Kemp 1980; Matveeva and Otten 2021). *Ar*ODH is also part of this structural class as outlined by several publications (Britton et al. 1998; Smits et al. 2008; Sharma et al. 2017). A general feature of this class is the two-domain structure, an NAD(P)H-binding domain at the N-terminus and a substrate binding domain at the C-terminus. The two domains form a cleft together, wherein catalysis can take place upon closure of the cleft by domain motions (Fig. 4A). On the first domain, a key structural motif is the Rossmann-fold helix (GxGxxG/A) responsible for cofactor binding, widely shared among NAD(P)^+^-dependent dehydrogenases. The substrate binding domain contains a few residues that are conserved among ODHs from any organism, such as a catalytic histidine-aspartate dyad or a key tryptophan residue in the active site (Fig. 4B). Based on these similarities, it can be assumed that findings describing the general mode of action of ODHs (Smits et al. 2010; Sharma et al. 2017; McFarlane et al. 2018) are applicable to the new mODHs as well.

However, the factors determining substrate specificity were still unclear, so we set out to investigate that by bioinformatic as well as experimental means. Comparing active site residues corresponding to those mutated by Codexis (see Table S5), mostly only subtle differences appear between *Ar*ODH and mODHs. A notable exception is the position 111 in *Ar*ODH, where an alanine is replaced by an arginine in all mODHs. We hypothesized that this change can be responsible for the substrate preference of mODHs towards amino acids with negatively charged side chains. Therefore, we performed mutagenesis on mODH-582 to have the variant R110A. We also created a “control mutant” *Ar*ODH A111R. Testing the activity of these variants on l-aspartate and l-phenylalanine and comparing them to the wild-type enzymes revealed loss of activity with the original substrates, but no gain of activity towards the other (Table S7). This result prompted us to further inspect the active site of ODHs, and we realized that the position 198 might be spatially correlated with the position 111. ArODH has an asparagine opposite to A111 while all mODHs possess glycine in that position leaving more space to the longer arginine side chain. Therefore, we prepared double mutant variants mODH-582 R110A G198N and *Ar*ODH A111R N198G. We tested these variants with l-aspartate and l-phenylalanine and observed a complete switch of substrate preference (Fig. 4C). We carried out the kinetic characterization of these variants and compared the results to the wild-type enzymes (Table 3). Here, we observed no apparent reaction with the non-preferred substrates; thus, the change in substrate preference could be clearly demonstrated.

**Table 3:** Kinetic parameters of ArODH and mODH-582 and the respective double mutant variants

Enzyme	l-Phe			l-Asp		
	*K*_M_ (mM)	*k*_cat_ (1/s)	*k*_cat_/*K*_M_ (s^−1^ mM^−1^)	*K*_M_ (mM)	*k*_cat_ (1/s)	*k*_cat_/*K*_M_ (s^−1^ mM^−1^)
ArODH	1.4 ± 0.5	861 ± 70	630	No reaction		
ArODH A111R N198G	No reaction			58.3 ± 19.6	2.8 ± 0.7	0.05
mODH-582	No reaction			24.8 ± 4.6	0.9 ± 0.1	0.04
mODH-582 R110A G198N	32.7 ± 4.7	1.66 ± 0.15	0.05	No reaction		

**Fig. 5:**
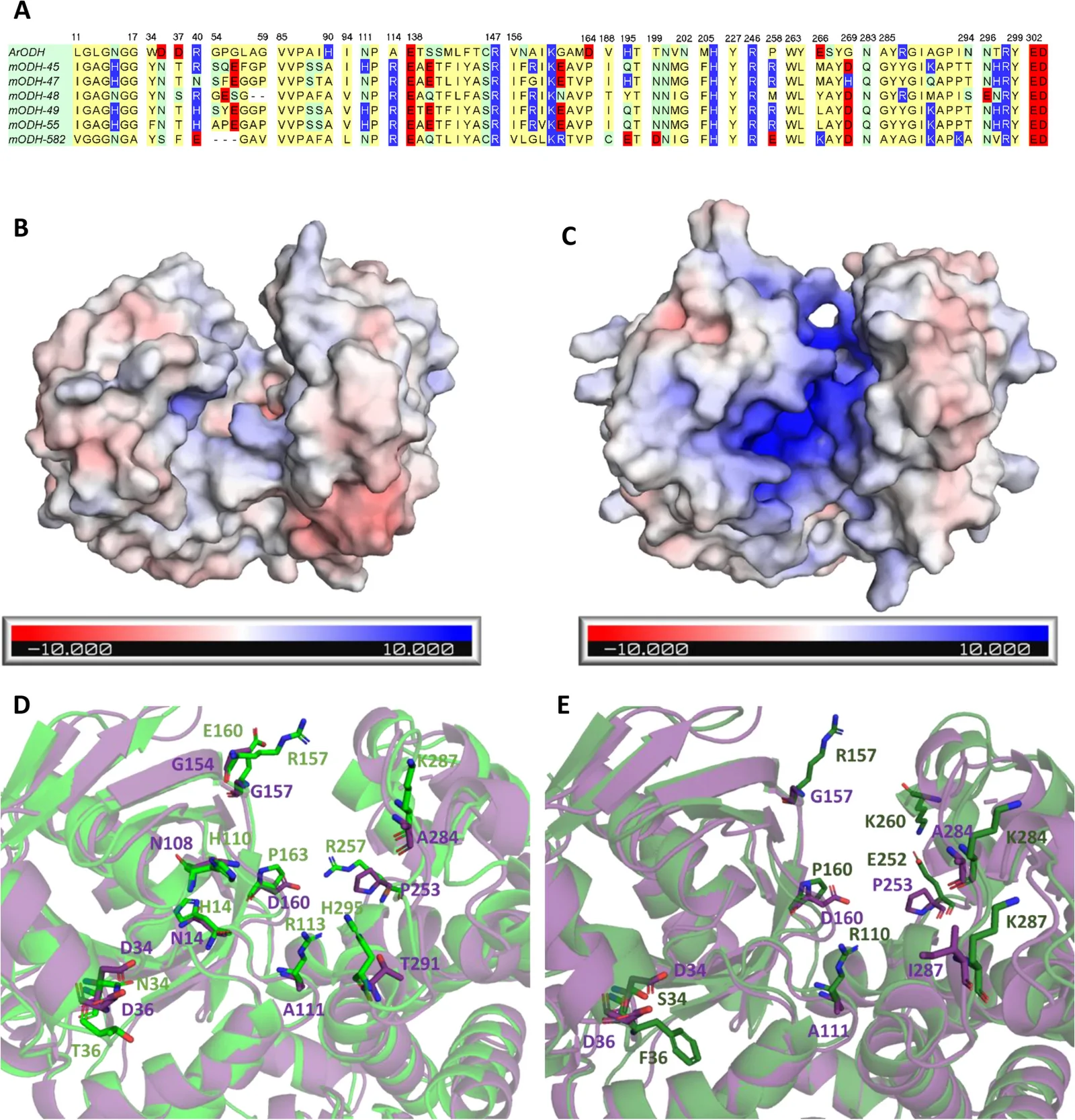
**A** Multiple sequence alignment of *Ar*ODH and six mODHs containing only residues forming the catalytic cleft between the two domains of the enzyme. Residues are colored by polarity/charge: apolar (yellow), polar (green), positively charged (blue), negatively charged (red). Relative positions of the alignment are displayed. **B** Electrostatic surface potential of ArODH. **C** Electrostatic surface potential of mODH-45. **D** Comparison of active sites of mODH-45 (green) and ArODH (purple). Residues contributing to the accumulation of positive charge inside the mODH active site are highlighted. **E** Comparison of active sites of mODH-582 (green) and ArODH (purple). Residues contributing to the accumulation of positive charge inside the mODH active site are highlighted

In addition to the site-directed mutagenesis studies, we decided to also take a holistic approach towards rationalizing the substrate preference of ODHs. We performed APBS (Adaptive Poisson-Boltzmann Solver) calculations to assess the electrostatic surface potential of mODHs. In accordance with our experimental results showing a preference of mODHs for negatively charged substrates, all mODHs show a remarkable accumulation of positive surface charge inside their active sites in strong contrast to the close to neutral surface potential of the *Ar*ODH active site (Fig. 5B and C, Figure S3). To follow up on this result, we wanted to identify specific residues that could be responsible for this charge accumulation inside the cleft between the two domains. To this end, we identified the residues lining the walls of this cleft using the Caver Web tool of Loschmidt Laboratories (Stourac et al. 2019). Then, we reduced a multiple sequence alignment of *Ar*ODH and mODHs to only contain these residues (Fig. 5A). Interestingly, we could not identify large differences in terms of charged residues. The only position, which contains a neutral to positive change for all mODHs, is the above-mentioned position 111 of *Ar*ODH. There are several other positions, however, that show positive charge changes (i.e., negative to neutral or neutral to positive), but not for all mODHs: 14 (N to H), 34 (D to N/S), 36 (D to T/S/F), 108 (N to H), 154 (A to R), 157 (G to R), 160 (D to P), 253 (P to R), 261 (E to M/Y/L/K), 284 (A to K), 287 (I to K), and 291 (T to H) (*Ar*ODH numbering is used) (exemplary cases shown on Fig. 5D and E).

### Preparative scale transformations and diastereoselectivity

**Scheme 2:**
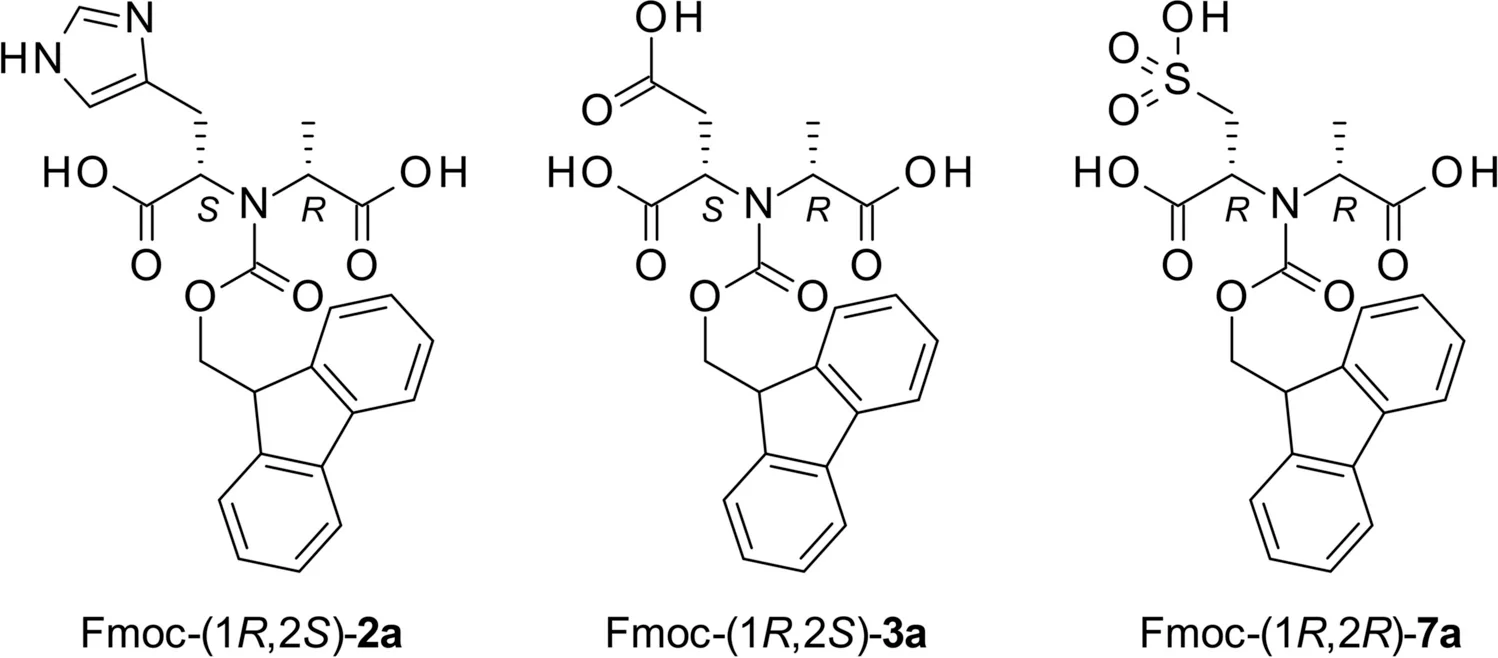
Structure of products isolated from preparative scale enzymatic reactions. Note: there is no switch in stereoselectivity in the case of **7a** but the substituent priority according to the CIP convention changed

After establishing the substrate preference of the newly discovered mODHs, we wanted to assess the diastereoselectivity of these enzymes and demonstrate their applicability in preparative scale biocatalysis. First, 100 mg scale transformations were conducted on l-histidine (**2**) with pyruvate (**a**) using mODH-582 and *Ar*ODH as this amino acid was accepted by both enzymes to a similar extent. The reactions were run until complete amino acid depletion was observed followed by our Fmoc-derivatization protocol. However, **2a** has poor retention on reverse-phase HPLC and gives low UV signal. Our attempts to use ion-exchange chromatography for product isolation failed, and derivatization was still necessary, but the opine-type secondary amines do not react with Fmoc-Cl under the conditions used for derivatization. Therefore, we scaled up and modified the protocol to yield better conversion to the Fmoc derivative of **2a** (Fmoc-**2a**). This compound was isolated from both reactions with yields 40% for *Ar*ODH and 20% for mODH. Meanwhile, we have also prepared reference compounds (Fmoc-(1*R*,2*S*)-2a* and Fmoc-(1*S*,2*S*)-**2a**) by chemical reductive amination of pyruvate (**a**) with l-histidine (**2**) (see “Materials and Methods”). The resulting diastereomers were isolated, and their NMR spectra were compared with the enzymatic products (Figure S4). Fmoc-(1*R*,2*S*)-2a* and Fmoc-(1*S*,2*S*)-**2a** give distinguishable signals, and the enzymatic products clearly correspond to Fmoc-(1*R*,2*S*)-2a*. In addition, comparison of the retention times of Fmoc-**2a** products of chemical and enzymatic origin also supports identical selectivity of *Ar*ODH and mODH-582 (Figure S5). Since the R-selectivity of *Ar*ODH is already well established (Asano et al. 1989), we concluded that mODH-582 has also R-stereoselectivity in the reductive amination. The diastereoselectivity of the rest of the mODHs was found identical to mODH-582 and *Ar*ODH by performing analytical scale reactions with l-histidine (**2**) and comparing the LC chromatograms after Fmoc-derivatization (Figure S6). We have also carried out preparative scale transformations with mODH-582 using the best substrates of mODHs, l-aspartate (**3**), and l-cysteic acid (**4**) with pyruvate (**a**) as ketoacid partner. The enzymatic reactions reached > 99% conversion, and the products were isolated as their Fmoc derivatives (24% for Fmoc-(1*R*,2*S*)-**3a**, 23% for Fmoc-(1*R*,2*R*)-**7a**, Scheme 2). Here, the configuration of the new stereocenter is assumed to be R based on the diastereoselectivity observed with l-histidine.

While scaling up the reactions with the preferred substrates, we have revisited the question of cofactor preference, which was only determined before in preliminary experiments with l-alanine and later corroborated by kinetic measurements (see above), but not in biocatalytic reactions. To our surprise, with l-aspartate, some enzymes (mODH-48, mODH-49, mODH-55, and mODH-582) have exhibited better conversions using NADH as cofactor (see Table S8). This result shows that the better kinetic parameters not necessarily translate into higher conversion values under biocatalytic reaction conditions. It also suggests that the cofactor preference of some mODHs might be substrate dependent.

## Discussion

Opine dehydrogenases are a diverse class of enzymes specialized to perform wide ranging physiological roles in organisms across the tree of life. Substrate specificities of each enzyme have evolved to perform a certain physiological function in the given organism. Mining opine dehydrogenases from metagenomes of extreme environments allows for identification of new enzymes with altered substrate specificities that could evolve under significantly different evolutionary pressure. In fact, the metagenomic opine dehydrogenases (mODHs) discovered within the framework of this study appear to be from an evolutionarily distant subclass of ODHs (Fig. 1) that was previously not described. Sequence identity to closest homologues deposited in UniProtKB varies between 62 and 99%, and all of them are unreviewed, while mODH-48 is identical to an obsolete entry in UniParc (Table S2). Their overall structure—as predicted by AlphaFold—resembles well other opine dehydrogenases even with very low sequence identity. However, they exhibit unique properties that make them a valuable addition to this underexplored enzyme class. Being derived from a hot spring metagenome, they show increased thermotolerance which is indicated by higher melting temperatures, resistance to heat inactivation, and increased optimum temperature. Moreover, their substrate preference towards negatively charged amino acid substrates is so far unprecedented for ODHs. The molecular background of the different amino acid substrate specificities of ODHs has not yet been elucidated to date. Several residues have been proposed to form key interactions in the active site with the amino acid substrate (Smits et al. 2008; McFarlane et al. 2018), but there was no indication that the amino acid preference would be tuneable by manipulating any of these residues. In this study, we demonstrated the switch of the amino acid preference in two investigated ODHs by mutating two spatially correlated positions. In addition to these two residues that directly influence substrate specificity, we have also considered global factors that can contribute to the preference of mODHs towards negatively charged amino acids. We have found that there is a significant accumulation of positive surface charge in the mODHs active site compared to the neutral surface of *Ar*ODH as revealed by APBS electrostatic potential calculations.

We have thoroughly analyzed the positions that can be responsible for this phenomenon and found that these positions are located mostly at the edges of the catalytic cleft rather than inside, where the catalysis takes place and negative charge changes can also be pinpointed. Overall, we can hypothesize that the preference of mODHs towards negatively charged substrates is aided by this positively charged microenvironment inside the catalytic pocket. The fact that substitutions generating this charged microenvironment are not identical for all mODHs suggests that they might have evolved independently from a common ancestor to perform the same or similar functions.

The newly discovered opine dehydrogenases have a clear preference for l-amino acids in line with previous observations (Telek et al. 2023). The diastereoselectivity (dictated by the stereoselectivity in the formation of the new stereocenter) also follows the general trend of ODHs, i.e., R-selectivity can be assumed in the reductive amination step based on experimental results with l-histidine.

**Fig. 6:**
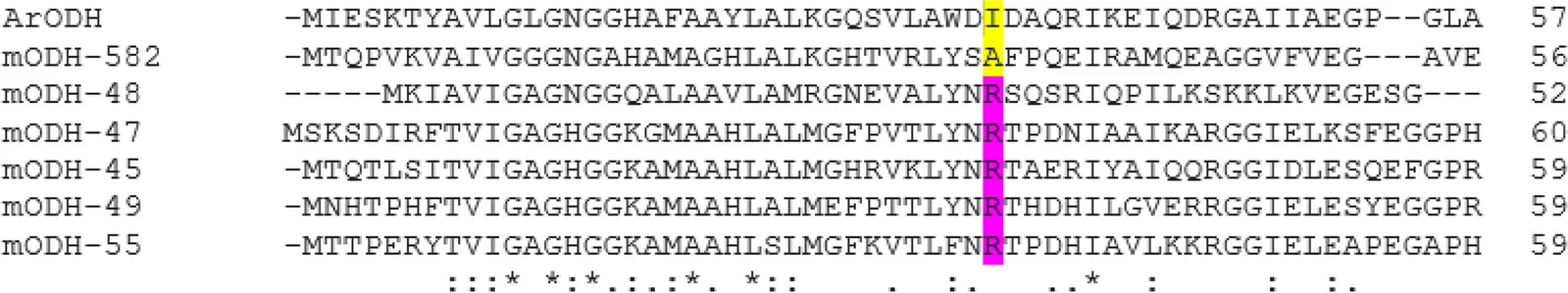
Multiple sequence alignment of *Ar*ODH an mODHs. Position 35 (ArODH numbering) is highlighted. An arginine (magenta) in this position indicates NADPH preference

The kinetic parameters of mODHs also align well with previous reports (Telek et al. 2023) even though their catalytic efficiency compared to ArODH (which is at the higher end of the reported values) was found to be lower (Table 1 and 3). This might be an adverse effect of their increased stability which can compromise flexibility reflected in lower *k*_cat_ values. The latter parameter could be targeted by enzyme engineering which would allow the exploitation of their superior thermal stability and distant substrate preference. However, it is worth mentioning that the better catalytic efficiency observed in kinetic measurements does not necessarily translate into higher conversions in biocatalytic reactions. One example is mODH-55 which shows superior kinetic parameters to most of other mODHs but fails to reach > 95% conversion with l-aspartate like others (Fig. 2 and Table 2). These discrepancies between kinetic measurements and biocatalytic reactions might be attributed to several factors (e.g., long-term enzyme stability and product inhibition) that can influence an enzyme’s stability in an overnight reaction in contrast to a quick kinetic measurement. These factors might also have an effect on the cofactor preference of these enzymes that is somewhat contradictory. In biocatalytic reactions with l-alanine, NADPH has proved to be preferred, and this preliminary observation was also corroborated by kinetic data measured with the best substrate l-aspartate (Table 2). A notable exception was mODH-582 that showed clear NADH preference while mODH-48 appeared to have no preference. However, in biocatalytic reactions, four of six enzymes performed better with NADH (see Table S8). The cofactor preferences revealed by the kinetic data can be explained by examining the cofactor binding site of the enzymes. Position 35 (*Ar*ODH numbering) has already been proposed to determine cofactor preference of ODHs in pathogenic bacteria (Laffont et al. 2019), where an arginine in that position ensures preferential binding of NADPH. In *Ar*ODH and mODH-582 that show clear NADH preference, aliphatic amino acids can be found in this position (alanine and isoleucine, respectively), while all other mODHs possess an arginine there (Fig. 6). This suggests that an arginine in position 35 is a good indicator that NADPH is accepted as a cofactor for the enzyme even if a clear preference towards it is not always observed. However, since these predictions are not necessarily translatable to behaviors in biocatalytic reactions (especially with different substrates), for further experiments on these enzymes, we suggest that cofactor preference should be established for each enzyme using the particular conditions and substrates for any future development.

In conclusion, with the discovery of metagenomic opine dehydrogenases (mODHs), we were able to add a new subclass to the opine dehydrogenase family with so far unprecedented substrate specificity. The newly identified enzymes have a strong preference for polar amino acids, especially those with negatively charged side chain. This preference is governed by two spatially correlated positions that can be used to switch substrate specificity of ODHs by site-directed mutagenesis. The preference towards negatively charged amino acids can be further rationalized by an overall positively charged surface area within the active site. Importantly, the six mODHs display higher melting temperature and better resistance to heat inactivation compared to the so far best characterized enzyme (*Ar*ODH) which is a promising property from an industrial perspective. Their enantio- and diastereoselectivity are proposed to be identical to known ODHs (preference for l-amino acids and R-selectivity on the newly form C-N bond). Overall, these enzymes offer good starting points for biocatalytic applications aiming at the synthesis of highly functionalized peptidomimetic building blocks.

## Supplementary Information

Below is the link to the electronic supplementary material.

Supplementary file1 (PDF 2.56 MB)

## Data Availability

Most data generated or analyzed during this study are included in this published article (and its supplementary information file).

## References

[CR1] Aleku GA, France SP, Man H, Mangas-Sanchez J, Montgomery SL, Sharma M, Leipold F, Hussain S, Grogan G, Turner NJ (2017) A reductive aminase from *Aspergillus**oryzae*. Nat Chem 9:961–969. 10.1038/nchem.2782 28937665 10.1038/nchem.2782

[CR2] Asano Y, Yamaguchi K, Kondo K (1989) A new NAD+-dependent opine dehydrogenase from *Arthrobacter* sp. strain 1C. J Bacteriol 171:4466–4471. 10.1128/jb.171.8.4466-4471.1989 2753861 10.1128/jb.171.8.4466-4471.1989 PMC210226

[CR3] Baud D, Jeffries JWE, Moody TS, Ward JM, Hailes HC (2017) A metagenomics approach for new biocatalyst discovery: application to transaminases and the synthesis of allylic amines. Green Chem 19:1134–1143. 10.1039/c6gc02769e

[CR4] Bhutani P, Joshi G, Raja N, Bachhav N, Rajanna PK, Bhutani H, Paul AT, Kumar R (2021) U.S. FDA approved drugs from 2015–June 2020: a perspective. J Med Chem 64:2339–2381. 10.1021/acs.jmedchem.0c01786 33617716 10.1021/acs.jmedchem.0c01786

[CR5] Britton KL, Asano Y, Rice DW (1998) Crystal structure and active site location of N-(1-D-carboxylethyl)-L-norvaline dehydrogenase. Nat Struct Biol 5:593–601. 10.1038/854 9665174 10.1038/854

[CR6] Caparco AA, Pelletier E, Petit JL, Jouenne A, Bommarius BR, Berardinis V, Zaparucha A, Champion JA, Bommarius AS, Vergne-Vaxelaire C (2020) Metagenomic mining for amine dehydrogenase discovery. Adv Synth Catal 362:2427–2436. 10.1002/adsc.202000094

[CR7] Chen H, Collier SJ, Nazor J, Sukumaran J, Smith D, Moore JC, Hughes G, Janey J, Huisman G, Novick S, Agard N, Alvizo O, Cope G (2013) Engineered imine reductases and methods for the reductive amination of ketone and amine compounds. US Pat. 2013/0302859 1:102pp.

[CR8] Cosgrove SC, Brzezniak A, France SP, Ramsden JI, Mangas-Sanchez J, Montgomery SL, Heath RS, Turner NJ (2018) Imine reductases, reductive aminases, and amine oxidases for the synthesis of chiral amines: discovery, characterization, and synthetic applications. In: Methods in Enzymology, 1st edn. Elsevier Inc., pp 131–149 10.1016/bs.mie.2018.04.022 30173761

[CR9] Ducrot L, Bennett M, Grogan G, Vergne-Vaxelaire C (2021) NAD(P)H-dependent enzymes for reductive amination: active site description and carbonyl-containing compound spectrum. Adv Synth Catal 363:328–351

[CR10] Eddy SR (2009) A new generation of homology search tools based on probabilistic inference. Genome Inform 23:205–211. 10.1142/9781848165632_0019 20180275

[CR11] Gao K, Oerlemans R, Groves MR (2020) Theory and applications of differential scanning fluorimetry in early-stage drug discovery. Biophys Rev 12:85–104 32006251 10.1007/s12551-020-00619-2 PMC7040159

[CR12] Gomm A, O’Reilly E (2018) Transaminases for chiral amine synthesis. Curr Opin Chem Biol 43:106–112. 10.1016/j.cbpa.2017.12.007 29278779 10.1016/j.cbpa.2017.12.007

[CR13] Hack E, Kemp JD (1980) Purification and characterization of the crown gall-specific enzyme, octopine synthase. Plant Physiol 65:949–955. 10.1104/pp.65.5.949 16661312 10.1104/pp.65.5.949 PMC440454

[CR14] Harcet M, Perina D, Pleše B (2013) Opine dehydrogenases in marine invertebrates. Biochem Genet 51:666–676. 10.1007/s10528-013-9596-7 23644944 10.1007/s10528-013-9596-7

[CR15] Kaličanin N, Balaž AM, Prodanović O, Prodanović R (2023) Heterologous expression and partial characterization of a putative opine dehydrogenase from a metagenomic sequence of *Desulfohalobium retbaense*. ChemBioChem e202300414. 10.1002/CBIC.202300414 10.1002/cbic.202300414 37531452

[CR16] Kato Y, Asano Y (2002) Opine dehydrogenase, secondary amine dicarboxylic acids. Encyclopedia of Industrial Biotechnology: Fermentation, Biocatalysis, and Bioseparation. John Wiley & Sons Inc, Hoboken, NJ, USA, pp 1851–1858

[CR17] Kato Y, Yamada H, Asano Y (1996) Stereoselective synthesis of opine-type secondary amine carboxylic acids by a new enzyme opine dehydrogenase use of recombinant enzymes. J Mol Catal B Enzym 1:151–160. 10.1016/1381-1177(95)00011-9

[CR18] Kerepesi C, Grolmusz V (2016) Evaluating the quantitative capabilities of metagenomic analysis software. Curr Microbiol 72:612–616. 10.1007/s00284-016-0991-2 26831696 10.1007/s00284-016-0991-2

[CR19] Kerepesi C, Grolmusz V (2016) Giant viruses of the Kutch Desert. Arch Virol 161:721–724. 10.1007/s00705-015-2720-8 26666442 10.1007/s00705-015-2720-8

[CR20] Kerepesi C, Grolmusz V (2017) The “Giant Virus Finder” discovers an abundance of giant viruses in the Antarctic dry valleys. Arch Virol 162:1671–1676. 10.1007/s00705-017-3286-4 28247094 10.1007/s00705-017-3286-4

[CR21] Kerepesi C, Bánky D, Grolmusz V (2014) AmphoraNet: the webserver implementation of the AMPHORA2 metagenomic workflow suite. Gene 533:538–540. 10.1016/J.GENE.2013.10.015 24144838 10.1016/j.gene.2013.10.015

[CR22] Kerepesi C, Szalkai B, Grolmusz V (2015) Visual analysis of the quantitative composition of metagenomic communities: the AmphoraVizu Webserver. Microb Ecol 69:695–697. 10.1007/s00248-014-0502-6 25296554 10.1007/s00248-014-0502-6

[CR23] Laffont C, Brutesco C, Hajjar C, Cullia G, Fanelli R, Ouerdane L, Cavelier F, Arnoux P (2019) Simple rules govern the diversity of bacterial nicotianamine-like metallophores. Biochem J 476:2221–2233. 10.1042/BCJ20190384 31300464 10.1042/BCJ20190384

[CR24] Leipold L, Dobrijevic D, Jeffries JWE, Bawn M, Moody TS, Ward JM, Hailes HC (2019) The identification and use of robust transaminases from a domestic drain metagenome. Green Chem 21:75–86. 10.1039/c8gc02986e 30930686 10.1039/c8gc02986e PMC6394892

[CR25] Li D, Liu CM, Luo R, Sadakane K, Lam TW (2015) MEGAHIT: an ultra-fast single-node solution for large and complex metagenomics assembly via succinct de Bruijn graph. Bioinformatics 31:1674–1676. 10.1093/bioinformatics/btv033 25609793 10.1093/bioinformatics/btv033

[CR26] Li Petri G, Di Martino S, De Rosa M (2022) Peptidomimetics: an overview of recent medicinal chemistry efforts toward the discovery of novel small molecule inhibitors. J Med Chem 65:7438–7475 35604326 10.1021/acs.jmedchem.2c00123

[CR27] Matveeva T, Otten L (2021) Opine biosynthesis in naturally transgenic plants: genes and products. Phytochemistry 189:112813. 10.1016/J.PHYTOCHEM.2021.112813 34192603 10.1016/j.phytochem.2021.112813

[CR28] McFarlane JS, Davis CL, Lamb AL (2018) Staphylopine, pseudopaline, and yersinopine dehydrogenases: a structural and kinetic analysis of a new functional class of opine dehydrogenase. J Biol Chem 293:8009–8019. 10.1074/jbc.RA118.002007 29618515 10.1074/jbc.RA118.002007 PMC5971449

[CR29] McFarlane JS, Lamb AL (2020) Opine metallophore biosynthesis. In: Comprehensive natural products III, 3rd edn. Elsevier, pp 395–414

[CR30] Mirdita M, Schütze K, Moriwaki Y, Heo L, Ovchinnikov S, Steinegger M (2022) ColabFold: making protein folding accessible to all. Nat Methods 19:679–682. 10.1038/s41592-022-01488-1 35637307 10.1038/s41592-022-01488-1 PMC9184281

[CR31] Narsing Rao MP, Luo Z-H, Dong Z-Y, Li Q, Liu B-B, Guo S-X, Nie G-X, Li W-J (2022) Metagenomic analysis further extends the role of *Chloroflexi* in fundamental biogeochemical cycles. Environ Res 209:112888. 10.1016/j.envres.2022.112888 35143804 10.1016/j.envres.2022.112888

[CR32] Niesen FH, Berglund H, Vedadi M (2007) The use of differential scanning fluorimetry to detect ligand interactions that promote protein stability. Nat Protoc 2007 29 2:2212–2221. 10.1038/nprot.2007.321 10.1038/nprot.2007.321 17853878

[CR33] Patil MD, Grogan G, Bommarius A, Yun H (2018) Oxidoreductase-catalyzed synthesis of chiral amines. ACS Catal 8:10985–11015. 10.1021/ACSCATAL.8B02924

[CR34] Robinson SL, Piel J, Sunagawa S (2021) A roadmap for metagenomic enzyme discovery. Nat Prod Rep 38:1994–2023. 10.1039/D1NP00006C 34821235 10.1039/d1np00006c PMC8597712

[CR35] Schober M, MacDermaid C, Ollis AA, Chang S, Khan D, Hosford J, Latham J, Ihnken LAF, Brown MJB, Fuerst D, Sanganee MJ, Roiban G-D (2019) Chiral synthesis of LSD1 inhibitor GSK2879552 enabled by directed evolution of an imine reductase. Nat Catal 2:909–915. 10.1038/s41929-019-0341-4

[CR36] Sharma M, Mangas-Sanchez J, Turner NJ, Grogan G (2017) NAD(P)H-dependent dehydrogenases for the asymmetric reductive amination of ketones: structure, mechanism, evolution and application. Adv Synth Catal 359:2011–2025. 10.1002/adsc.201700356 30008635 10.1002/adsc.201700356 PMC6033044

[CR37] Sievers F, Wilm A, Dineen D, Gibson TJ, Karplus K, Li W, Lopez R, McWilliam H, Remmert M, Söding J, Thompson JD, Higgins DG (2011) Fast, scalable generation of high-quality protein multiple sequence alignments using Clustal Omega. Mol Syst Biol 7:539. 10.1038/msb.2011.75 21988835 10.1038/msb.2011.75 PMC3261699

[CR38] Smits SHJ, Mueller A, Schmitt L, Grieshaber MK (2008) A structural basis for substrate selectivity and stereoselectivity in octopine dehydrogenase from *Pecten**maximus*. J Mol Biol 381:200–211. 10.1016/j.jmb.2008.06.003 18599075 10.1016/j.jmb.2008.06.003

[CR39] Smits SHJ, Meyer T, Mueller A, van Os N, Stoldt M, Willbold D, Schmitt L, Grieshaber MK (2010) Insights into the mechanism of ligand binding to octopine dehydrogenase from *Pecten maximus* by NMR and crystallography. PLoS One 5. 10.1371/journal.pone.0012312 10.1371/journal.pone.0012312 PMC2924402 20808820

[CR40] Stourac J, Vavra O, Kokkonen P, Filipovic J, Pinto G, Brezovsky J, Damborsky J, Bednar D (2019) Caver Web 1.0: identification of tunnels and channels in proteins and analysis of ligand transport. Nucleic Acids Res 47:W414–W422. 10.1093/NAR/GKZ378 31114897 10.1093/nar/gkz378 PMC6602463

[CR41] Szalkai B, Grolmusz V (2019) MetaHMM: a webserver for identifying novel genes with specified functions in metagenomic samples. Genomics 111:883–885. 10.1016/J.YGENO.2018.05.016 29802977 10.1016/j.ygeno.2018.05.016

[CR42] Tassano E, Moore C, Dussauge S, Vargas A, Snajdrova R (2022) Discovery of new Fe(II)/α-ketoglutarate-dependent dioxygenases for oxidation of l -proline. Org Process Res Dev 26:1996–2003. 10.1021/acs.oprd.1c00405

[CR43] Telek A, Molnár Z, Vértessy BG, Tasnádi G (2023) Opine dehydrogenases, an underexplored enzyme family for the enzymatic synthesis of chiral amines. Biotechnol Bioeng 120:2793–2808. 10.1002/bit.28469 37334502 10.1002/bit.28469

[CR44] Yoon B-J (2009) Hidden Markov models and their applications in biological sequence analysis. Curr Genomics 10:402–415. 10.2174/138920209789177575 20190955 10.2174/138920209789177575 PMC2766791

[CR45] Zallot R, Oberg N, Gerlt JA (2019) The EFI web resource for genomic enzymology tools: leveraging protein, genome, and metagenome databases to discover novel enzymes and metabolic pathways. Biochemistry 58:4169–4182. 10.1021/ACS.BIOCHEM.9B00735 31553576 10.1021/acs.biochem.9b00735 PMC7057060

