# Supplementary material for: Discovery and biocatalytic characterization of opine dehydrogenases by metagenome mining
Source: Appl Microbiol Biotechnol. 2024 Jan 13;108:101. doi: 10.1007/s00253-023-12871-z (PMC10787698; doi:10.1007/s00253-023-12871-z)
Supplement: Supplementary file 1 [file 253_2023_12871_MOESM1_ESM.pdf]

Applied Microbiology and Biotechnology

Supplementary Information

Discovery and biocatalytic characterization of opine dehydrogenases by metagenome mining

András Telek,[1,2] Zsófia Molnár,[3,4] Kristóf Takács,[5] Bálint Varga,[5] Vince Grolmusz,[5] Gábor Tasnádi,[2]* Beáta G. Vértessy[1,3]*

[1] Department of Applied Biotechnology, Budapest University of Technology and Economics, Budapest, Hungary

[2] Servier Research Institute of Medicinal Chemistry, Budapest, Hungary

[3] Institute of Molecular Life Sciences, Research Centre for Natural Sciences, HUN-REN, Budapest, Hungary

[4] Department of Organic Chemistry and Technology, Budapest University of Technology and Economics, Budapest, Hungary

[5] PIT Bioinformatics Group, Institute of Mathematics, Eötvös University, Budapest, Hungary

Corresponding authors: Beáta G. Vértessy, vertessy.beata@ttk.hu, +36 1 382 6707 and Gábor Tasnádi, gabor.tasnadi@servier.com, +36 1 881 2010

Content

Table S1 - Protein sequences used as templates for metagenome search 3

Table S2 – Full length ODH protein sequences extracted from the Sativali hot spring metagenome and their synthetic nucleotide sequences. 6

Table S3 - ~~Screening results~~ Conversion data with all amino acid substrates.[a,b] 13

Table S4 - ~~Screening results~~ Conversion data with all ketoacid substrates.[a,b] 15

Table S5 – Comparison of key active site residues of ODHs, that were mutated by Codexis when engineering ArODH 15

Table S6 – Primers used for mutagenesis 16

Table S7 – Conversions obtained with mutant and wild type enzymes. 17

Table S8 – Conversion data for cofactor preference study of mODHs with L-aspartate.[a,b] 18

Table S9 – Kinetic constants of ODHs with L-aspartate. The corresponding Michaelis-Menten curves can be seen on Figure S15. 19

Table S10 – Kinetic constants of ODHs with L-phenylalanine. The corresponding Michaelis-Menten curves can be seen on Figure S16. 19

Table S11 – Kinetic constants of ODHs with NADPH. The corresponding Michaelis-Menten curves can be seen on Figure S17. 19

Table S12 – Kinetic constants of ODHs with NADH. The corresponding Michaelis-Menten curves can be seen on Figure S18. 20

Figure S1 – Representative SDS-PAGE analysis of the Ni-affinity purification of mODHs. 21

Figure S2 – Melting curves of ODHs measured by differential scanning fluorimetry. 21

Figure S3 – Surface potential of ArODH and all mODHs 22

Figure S4 – Comparison of NMR spectra of enzymatic products with chemically synthesized reference compounds 23

Figure S5 – Comparison of HPLC/UV chromatograms of enzymatic products with chemically synthesized reference compounds 24

Figure S6 – Comparison of HPLC/UV chromatograms of *Ar*ODH and mODHs in enzymatic reaction with L-histidine (2) and pyruvate (a) with chemically synthesized reference compounds 25

Figure S7 - ^{1}H-NMR spectrum of Fmoc-(1*R*,2*S*)-2a (prepared by mODH-582) 26

Figure S8 - ^{13}C-NMR spectrum of Fmoc-(1*R*,2*S*)-2a (prepared by mODH-582) 27

Figure S9 - ^{1}H-NMR spectrum of Fmoc-(1*S*,2*S*)-2a 28

Figure S10 - ^{13}C-NMR spectrum of Fmoc-(1*S*,2*S*)-2a 29

Figure S11 - ^{1}H-NMR spectrum of Fmoc-(1*R*,2*S*)-3a 30

Figure S12 - ^{13}C-NMR spectrum of Fmoc-(1*R*,2*S*)-3a 31

Figure S13 - ^{1}H-NMR spectrum of Fmoc-(1*R*,2*R*)-7a 32

Figure S14 - ^{13}C-NMR spectrum of Fmoc-(1*R*,2*R*)-7a 33

Figure S15 – Measured Michaelis-Menten curves of ODHs for l-aspartate. 34

Figure S16 – Measured Michaelis-Menten curves of ODHs for L-phenylalanine. 35

Figure S17 – Measured Michaelis-Menten curves of ODHs for NADPH. 36

Figure S18 – Measured Michaelis-Menten curves of ODHs for NADH. 37

Table S1 - Protein sequences used as templates for metagenome search

UniprotID	***Name***	***Organism***	***Sequence***
Q44297	Opine dehydrogenase (N-(1-D-carboxyethyl)-L-norvaline dehydrogenase)	*Arthrobacter* sp. (strain 1C)	MIESKTYAVLGLGNGGHAFAAYLALKGQSVLAWDIDAQRIKEIQDRGAIIAEGPGLAGTAHPDLLTSDIGLAVKDADVILIVVPAIHHASIAANIASYISEGQLIILNPGATGGALEFRKILRENGAPEVTIGETSSMLFTCRSERPGQVTVNAIKGAMDFACLPAAKAGWALEQIGSVLPQYVAVENVLHTSLTNVNAVMHPLPTLLNAARCESGTPFQYYLEGITPSVGSLAEKVDAERIAIAKAFDLNVPSVCEWYKESYGQSPATIYEAVQGNPAYRGIAGPINLNTRYFFEDVSTGLVPLSELGRAVNVPTPLIDAVLDLISSLIDTDFRKEGRTLEKLGLSGLTAAGIRSAVE
Q9BHM6	Octopine dehydrogenase (OcDH)	*Pecten maximus* (King scallop) (Pilgrim's clam)	MTVKVCVCGGGNGAHTLSGLAASRDGVEVRVLTLFADEAERWTKALGADELTVIVNEKDGTQTEVKSRPKVITKDPEIAISGADVVILTVPAFAHEGYFQAMAPYVQDSALIVGLPSQAGFEFQCRDILGDKAAAVSMMSFETLPWACRIKEFGRKVEVLGTKSVLAASLIKGTAKTVDPLSTLQMLHGAEPVFRLAKHFLEMLIMSYSFVHPAILFGRWGSWDGKPVPEAPLFYQGIDQATADMLTACSNECKDVANAIMAACPGNDLSDVKDIYQWYLEYYHEDIQDDHDLYHAITTNKSYKGLVHPVKAVDGGVAPDFGNRYLTEDIPMGMIVFKGVAIAAGVAIPSNDKLIMWAQEKIGKEYLVDGALTGKDVATTRCPQRYGFNTLDAILTGKK
Q9HUX5	Pseudopaline synthase (Opine dehydrogenase) (ODH) (Pseudopaline dehydrogenase)	*Pseudomonas aeruginosa*	MNAADESLGNVLLVGLGAVAIQVALDLRRHGAGRLGALNHPGRRSQRIAEALARGACLQLEGQGQHRWLSGNAALDVFHQDPAELRDDWQTLVLCVPADSYLDVVRGLPWERLGGVRTLLLVSAFIGANLLVRSALPAGCQATVLSLSSYYAATKVIDETQPLRALTKAVKRRVYLGSSRPDCPARETWRRVLAGSGVEVVPLATPEAAEGRNVTTYVHSPFFLGEFALARILSEQGPPGFMYKLYPEGPITPGAIGAMRRLWCELSELLRRMGAEPLNLLRFLNDDNYPVHETMLPRASIDGFAEAGAERQEYLLFVRYAALLVDPFSPADEQGRHFDFSAVPFRRVSRDEDGLWRLPRVPLEDYRKLALIVALAAHFDLAMPQARSLLASYENAVSRFIDCQGASQCHPSLYPIDSRPAADAIYRQWCSTC
A0A0H3JT80	Staphylopine synthase (Opine	*Staphylococcus aureus*	MSKLLMIGTGPVAIQLANICYLKSDYEIDMVGRASTSEKSKRLYQAYKKEKQFEVKIQNEAHQHLEGKFEINRLYKDVKNVKGEYETVVMACTADAYYDTLQQLSLETLQSVKHVILI

	dehydrogenase) (ODH) (Staphylopine dehydrogenase)		SPTFGSQMIVEQFMSKFSQDIEVISFSTYLGDTRIVDKEAP NHVLTTGVKKKLYMGSTHSNSTMCQRISALAEQLKIQLE VVESPLHAETRNSSLYVHPPLFMNDFSLKAIFEGTDVPVY VYKLFPEGPITMTLIREMRLMWKEMMAILQAFRVPSVNL LQFMVKENYPVRPETLDEGDIEHFEILPDILQEYLLYVRYT AILIDPFSQPDENGHYFDFSAVPFKQVYKNEQDVVQIPRM PSEDYYRTAMIQHIGKMLGIKTPMIDQFLTRYEASCQAYK DMHQDQQLSSQFNTNLFEGDKALVTKFLEINRTLS
Q8CKU7	Yersinopine synthase (Opine dehydrogenase) (ODH) (Yersinopine dehydrogenase)	*Yersinia pestis*	MHNTLPTLILGAGPAAIQLAVDISATGDARLGLYNRPSTK GERLKQYLALTPTLYLQGTGKAQATQKESSVTIDCYIDQL AQAVGDWQRLILAVPADHYYAVLQQIPWAALPQLKSVILL SSSMGSGLMVQNLLNAAGKRDVEVISLSSYYADTKYIRA ETQDISANTQDINAGTQDIGAIQPYRAYTKAFKQRIYLAN QWGNAGSAEMSWLTAVLARHHIDTLPCSNLLAAERFSIT NYVHPPLALADTTLQALFYPEQRSQYLYKTQPEGPVCPAV IADLAGLADDYKRLLNRLGVEEINLLRFLNDDNYPVPAS MVSRRWIDEFPQLPPLEQQYALFVRYTALLVDPYSTPDEQ GRFYDFSAVKVATVYQDANALWHLPRVPLEDVHKLRTLL LLAGALDVVMPTAQRLLQRFQQALKAFIDRVGEEHCHPS LLGDDCDRQAAIIEQQWRSQT
M1FK93	D-octopine dehydrogenase	*Mytilus galloprovincialis* (Mediterranean mussel)	MSEKCRILVCGGGNGAHCLSALAASKDNLDVHVLTLYQD EAERWAKHVENENMKLTITKQDGTQYDTFSKPSLITKDA AKAMSGVEIVFLVVPAFAHAQYFTAIAPHLQPNTLIVGLP GQAGFELQCRHIIGDKASSCTIAASESLPFACRIVEFGRHA RILGLKDSLGMSALKGKACQLSFPVVETVQGILGEYPKLE LLKNYIAINLMADANVHPPMMYGRWGNWDGKPLKEEPL FYQGVDDRQADLLSRVSEELLAAAKAIEQKRKDVDMSE VIHLFDWYKIHYPDQITDKSSLKMAMRTNKAYDGLVHPM VKTDEGYVPNFNYRYTSEDVPFGMVVMKGIADLAGVPT PAMDEILAWGQQKLGKEYIVGSKLIGKDIGLARAPQSFG MTSVDELFDI
B5D5P2	Alanopine dehydrogenase	*Arenicola marina* (Lugworm) (Lumbricus marinus)	MGDSDITTRLLICGGGNGAHAYAGIASSKKNVEVRVLTLY SDEAERWTNAMENHDFVVNFDCGGKHNGEIRAKPSMVS KDPAEVALGCNVIVIVVPAFAHEQYLRALEPYIQPGTIIVG MPGQAGFEFACWGILGAKGKQCTLVSFESLPWACRIEEFG RTAEVKGTKQQLVGALLYGDPRPVREPSAVLQETLGTHP VLHIHGHLLGMTLMGVNAYIHPAILYGRWWNWDGRPVA

			EPPLFYHGMDEFTADTLTAMGDEVMAIAATLSKHPAGPD<br>FSNVCHVFDWYKRCYSDEMSDTSCLLKAIRSNKAYIGLT<br>HPCRPAEAAGDNNGGCNWEPNFGYRYITEDIPFGLVVLC<br>GVAQIIGVATPHMDKVVLWSQKVSGKEYLVDSTLSGRDM<br>PDTRSPQKYGLTTIDQILGYD
A0A9I9	Strombine/ alanopine dehydrogenase	*Marphysa sanguinea* (Polychaete worm) (Nereis sanguinea)	MVVLVICGGGNGAHVLAGIASSNPNADVRVLTLYADEAE<br>RWTKAMEGNDFVVTVNNQDKSQTILKNKPRLVTKDPAL<br>AANKADMIVFTVPAFAHRQYLDALKPHIRPGTVLVGLPG<br>QSGFEFEVWTAWGDLAKQCSIMSFESLPWACRFTEFGRA<br>ATVIGTKENLAGAAWYGSVPPKADPTLVLQGCLGPHPVL<br>LTRGALLGITLMATNGYIHPSILFGRWHKWDGNPVNEPPL<br>FYNGLDEFSAQTMSSVSDEILAVASSLMKQRPQVDLTNVA<br>HIWQWYLRVYADDIGDKTSLFTTIRTNAAYSGLTHPTTKT<br>DDGKFVPDFKHRYLMEDIPFGLLVSKGIAEVAGVPTPTIDS<br>VISWAQQKMGKEYIVNGKLAGKDVSSTRCPQRYGLTTVD<br>AILGL
A0A9J0	Alanopine dehydrogenase	*Marphysa sanguinea* (Polychaete worm) (Nereis sanguinea)	MVVVVVCGGGNGAHTLAGVAASRPNTEVRVLTLFADEA<br>ERWTKAMESSDFVTNIHNHDKSITKLTNKPRLVTKDPGK<br>AAIGAEIIILAVPAFVHAQYLEALKPHVTPGTILVGLPGQPG<br>FEFDVYTIWGPLASKCSIMAFESLPWACRIAEFGQCVEILG<br>TKESLVGAVHIGSVPSKFDPTLLLQGCLGPHPSLLTKGHLL<br>GITLMSVNSYIHSSILYGRWHDWNGTPLDEAPLFYQGIDQ<br>DTADLMSGMSDEILVIAKEIMKKNPKVDLHNVDHVLQW<br>YLRVYEDDIQDKSNLYTAIRSNEAYIGLTHPMSKTEDGKY<br>LPNFKHRYLSEDIPYGLAVSKGVAEVVGVNTPLIDKVLIW<br>CQEKLGKEYLVDGKMIGKDVTFTRCPQRYSMTTVDAILG<br>K
P0A395	Protein ocs; D-octopine dehydrogenase (Octopine synthase)	*Agrobacterium tumefaciens* (strain 15955)	MAKVAILGAGNVALTLAGDLARRLGQVSSIWAPISNRNSF<br>NSVRSLGSLELVGPDYGGDFQPQLEDDLETAISGAAFIFLT<br>VPTMGQQGILCELANFNLSSSVLVALPGSATSLACKQTLTP<br>AFAPIAVIEATTSPYACRRVNAQVLMLSVKRTFEVASTQAL<br>SEEVRGGFEILFPNRLQWYQNPASIFFSNTNPVAHPAGILA<br>AKDTIEQGISPIPKFYRKFVPQAITRVTAIDEERLTIVNALG<br>LESETDFAYCKKWYGGHASNAREFYETFEGYADIETPRN<br>MNHRYLSEDVKHILVLWVEIAEVIGVQVPEMKSVVQEAS<br>DVLNEDLSHTGRGLSSLNLEGSNANAIVRALNGV

Table S2 – Full length ODH protein sequences extracted from the Sativali hot spring metagenome and their synthetic nucleotide sequences.

Sequences that were not expressed due to the lack of a consensus cofactor binding sequence are labelled orange. Sequences are labelled yellow, where the expressed enzyme was not soluble. Sequences are labelled green, where the soluble expression allowed detailed characterization of the enzyme.

>mODH-43 (Nucleotide sequence accession number in the TPA Section of the DDBJ/ENA/GenBank databases: BK063522)<br>Closest homologue in UniProt: A0A7X9EIT5 (99%)
MAGHLAIMGFDVKLYNRSEERLRAVKMEGGIDLTGEVTGFGKVQMATSNPEEAVADVDVIMIVVPATAHKNLAKIFAPYLKSGQ<br>IVILNPGRTFGALEFSQILKNEGASEDITVGETQTFLYASRVTGPGQAKIFRIKNSVPLATVRAFKIPQTLSVIRTAFPHFVSGDSIFKT<br>SFDNIGCVFHPSLVVLNAGWIEDPSDFEFYFQGTTLSTSKILEQLDKERLSVASALGFRAMSAREWLYYAYDVSGKTLYEAIHSNP<br>GYRGIMAPHNTNMRYITEDVPCSLVPMSSIGKKFNVQTPLIDSIIRIASAMHETDYYKLGRTVESLGIENMDLKQLRLIAIGEHPN
ATGGCGGGTCACCTGGCGATCATGGGCTTTGACGTGAAGCTGTACAACCGTAGCGAGGAACGTCTGCGTGCGGTTAAAATG<br>GAGGGTGGCATTGATCTGACCGGTGAAGTGACCGGTTTCGGCAAGGTTCAGATGGCGACCAGCAACCCGGAGGAAGCGGT<br>GGCGGATGTGGATGTTATCATGATTGTGGTTCCGGCGACCGCGCACAAGAACCTGGCGAAAATCTTTGCGCCGTACCTGAAG<br>AGCGGCCAGATCGTTATTCTGAACCCGGGTCGTACCTTTGGCGCGCTGGAGTTCAGCCAAATCCTGAAAAACGAGGGTGCG<br>AGCGAAGACATTACCGTGGGCGAAACCCAGACCTTCCTGTATGCGAGCCGTGTTACCGGTCCGGGTCAAGCGAAGATCTTT<br>CGTATTAAAAACAGCGTGCCGCTGGCGACCGTTCGTGCGTTCAAGATCCCGCAAACCCTGAGCGTGATTCGTACCGCGTTTC<br>CGCACTTCGTTAGCGGTGACAGCATCTTTAAAACCAGCTTCGATAACATTGGCTGCGTGTTTCACCCGAGCCTGGTGGTTCT<br>GAACGCGGGTTGGATCGAGGACCCGAGCGATTTCGAATTTTATTTCCAGGGCACCACCCTGAGCACCAGCAAGATTCTGGA<br>GCAACTGGACAAAGAACGTCTGAGCGTGGCGAGCGCGCTGGGTTTCCGTGCGATGAGCGCGCGTGAGTGGCTGTACTATGC<br>GTACGATGTTAGCGGCAAAACCCTGTATGAAGCGATCCACAGCAACCCGGGTTACCGTGGCATTATGGCGCCGCACAACAC<br>CAACATGCGTTATATCACCGAGGACGTGCCGTGCAGCCTGGTTCCGATGAGCAGCATTGGTAAGAAATTTAACGTGCAGACC<br>CCGCTGATCGACAGCATCATTCGTATTGCGAGCGCGATGCACGAGACCGATTACTATAAGCTGGGTCGTACCGTTGAGAGCC<br>TGGGCATCGAAAACATGGATCTGAAACAACTGCGTCTGATCGCGATTGGTGAACACCCGAACTAA
>mODH-44 (Nucleotide sequence accession number in the TPA Section of the DDBJ/ENA/GenBank databases: BK063523)<br>Closest homologue in UniProt: A0A1F9ZJZ8 (83%)
MGLGHKKSRKERVPTFAVLGAGHGGLAMAGHLALMGFPTRIWNRSRERIESVQDRGGIDVEGIVDGVGRVDIATSDMGEALDG<br>ADVVMVVVPASGHRYVAERAAPHLKSNQIVVLNPGRTFGALEFLQVLKEQKTKTKPIISEAQTFLYVSRHSEPARARIIQIKNSVP<br>LAAIPAHKTPEVLEEVRKAFPQFVAASNVLETSLDNIGAIFHPGLTILNAGRIESTHGDFEYYLEGVSPSTAKILEAADAERVAIGSA<br>LGIHLHTAREWLYLAYDSPGKTLYEAIQATPGYKGVRAPATLTHRYLLEDVPMSLVPMVSVGQQIGVKTPTLAALIHLASTFHSR<br>DFWAEGRTVERVGLKGKSVKDIRLLAVRGISR
ATGGGTCTGGGCCACAAGAAAAGCCGTAAAGAGCGTGTTCCGACCTTTGCGGTTCTGGGTGCGGGTCATGGTGGCCTGGCG<br>ATGGCGGGTCACCTGGCGCTGATGGGCTTTCCGACCCGTATCTGGAACCGTAGCCGTGAGCGTATTGAAAGCGTGCAGGAC<br>CGTGGTGGCATCGATGTTGAGGGTATTGTTGACGGTGTGGGCCGTGTTGACATCGCGACCAGCGATATGGGTGAAGCGCTGG<br>ACGGCGCGGATGTGGTTATGGTGGTTGTTCCGGCGAGCGGTCACCGTTATGTGGCGGAACGTGCGGCGCCGCACCTGAAAA<br>GCAACCAAATTGTTGTGCTGAACCCGGGTCGTACCTTCGGCGCGCTGGAGTTTCTGCAGGTTCTGAAGGAACAAAAGACCA

AAACCAAGCCGATCATTAGCGAGGCGCAGACCTTCCTGTATGTTAGCCGTCACAGCGAACCGGCGCGTGCGCGTATCATTCA
AATCAAAAACAGCGTTCCGCTGGCGGCGATTCCGGCGCACAAAACCCCGGAAGTGCTGGAGGAAGTTCGTAAGGCGTTCC
CGCAGTTTGTGGCGGCGAGCAACGTTCTGGAAACCAGCCTGGATAACATCGGTGCGATTTTTCATCCGGGTCTGACCATCCT
GAACGCGGGTCGTATTGAGAGCACCCACGGCGACTTTGAGTACTATCTGGAAGGTGTGAGCCCGAGCACCGCGAAGATCCT
GGAGGCGGCGGATGCGGAACGTGTTGCGATCGGTAGCGCGCTGGGCATTCACCTGCACACCGCGCGTGAGTGGCTGTACCT
GGCGTATGACAGCCCGGGTAAAACCCTGTACGAAGCGATCCAAGCGACCCCGGGTTATAAGGGCGTGCGTGCGCCGGCGAC
CCTGACCCACCGTTACCTGCTGGAAGATGTGCCGATGAGCCTGGTTCCGATGGTTAGCGTGGGTCAGCAAATCGGCGTTAAA
ACCCCGACCCTGGCGGCGCTGATTCACCTGGCGAGCACCTTCCACAGCCGTGACTTTTGGGCGGAGGGTCGTACCGTGGAA
CGTGTTGGTCTGAAAGGCAAGAGCGTGAAGGATATCCGTCTGCTGGCGGTTCGTGGCATTAGCCGTTAA

>mODH-45 (Nucleotide sequence accession number in the TPA Section of the DDBJ/ENA/GenBank databases: BK063524)

Closest homologue in UniProt: E8MYZ6 (93%)

MTQTLSITVIGAGHGGKAMAAHLALMGHRVKLYNRTAERIYAIQQRGGIDLESQEFGPRGFGKLECATSNIEEALKGSQLIMVVV
PSSAHADIARAAAPYLQDGQVVVLHPGRTCGAIEFTMVMRRSGCQADVIVAEAETFIYASRSDGPAQARIFRIKEAVPLAALPAK
RTREALELVNIVYPQFIDGGNVLQTGLNNMGAIFHPALTLLNAGWIEATHGDYQFYIDGVTPSVARVLEALDRERITVAASLGIRA
RSALEWLKMAYDAVGNDLREAIHNQPGYYGIKAPTTLNHRYIFEDVPMSLVPIASLGMQYGVSVRGMDSIIRLACIVHNTDYWR
RGRTVENLGLRGLSVEELTHFVMEGELAV

ATGACCCAGACCCTGAGCATCACCGTTATTGGTGCGGGTCATGGTGGCAAAGCGATGGCGGCGCACCTGGCGCTGATGGGT
CACCGTGTGAAACTGTACAACCGTACCGCGGAGCGTATCTATGCGATTCAGCAACGTGGTGGCATCGACCTGGAGAGCCAG
GAATTCGGTCCGCGTGGTTTTGGCAAGCTGGAATGCGCGACCAGCAACATCGAGGAAGCGCTGAAAGGCAGCCAACTGAT
TATGGTGGTTGTGCCGAGCAGCGCGCATGCGGACATTGCGCGTGCGGCGGCGCCGTACCTGCAGGATGGCCAAGTTGTTGT
TCTGCACCCGGGTCGTACCTGCGGTGCGATTGAGTTCACCATGGTTATGCGTCGTAGCGGTTGCCAAGCGGATGTGATTGTT
GCGGAGGCGGAAACCTTCATTTATGCGAGCCGTAGCGATGGTCCGGCGCAAGCGCGTATCTTTCGTATTAAGGAAGCGGTTC
CGCTGGCGGCGCTGCCGGCGAAACGTACCCGTGAGGCGCTGGAACTGGTGAACATCGTTTATCCGCAGTTCATTGACGGTG
GCAACGTGCTGCAAACCGGTCTGAACAACATGGGCGCGATCTTTCATCCGGCGCTGACCCTGCTGAACGCGGGTTGGATTG
AGGCGACCCATGGTGACTACCAATTTTATATTGATGGTGTTACCCCGAGCGTGGCGCGTGTTCTGGAGGCGCTGGATCGTGA
ACGTATCACCGTGGCGGCGAGCCTGGGTATTCGTGCGCGTAGCGCGCTGGAGTGGCTGAAGATGGCGTACGACGCGGTGGG
CAACGATCTGCGTGAAGCGATCCACAACCAGCCGGGTTACTATGGCATTAAAGCGCCGACCACCCTGAACCACCGTTACATC
TTCGAGGATGTGCCGATGAGCCTGGTTCCGATTGCGAGCCTGGGTATGCAATATGGCGTGAGCGTTCGTGGTATGGACAGCA
TCATTCGTCTGGCGTGCATCGTTCACAACACCGATTATTGGCGTCGTGGCCGTACCGTGGAAAACCTGGGTCTGCGTGGCCT
GAGCGTGGAGGAACTGACCCACTTTGTTATGGAGGGTGAACTGGCGGTGTAA

>mODH-47 (Nucleotide sequence accession number in the TPA Section of the DDBJ/ENA/GenBank databases: BK063525)

Closest homologue in UniProt: A0A7C7RQQ9 (80%)

MSKSDIRFTVIGAGHGGKGMAAHLALMGFPVTLYNRTPDNIAAIKARGGIELKSFEGGPHGFAMLAKATSDIEEALKECDVIMV
VVPSTAHAEIARSLAPHLKDGQIIVLNPGRTCGALEVKHTLEENGCSADVTVAEAETLIYASRSDGPAQARIFGIKETVPLAALPAT
RTQLVLDTLAPAYPQFIDGGSVLHTGLNNMGAIFHPALALLNAGRIESTQGEFQFYIDGVSPSVARVLEVLDRERVTVASALGIRA
RTALEWLQMAYHATGNDLYEAIHNQPGYYGIQAPPTLNHRYITEDVPMSLVPIAALGERYGVSVRGMESIIRLACIIHRTDYWRR
GRTLEKLGIHDWSVSELTKYVMEGMV

ATGAGCAAGAGCGACATCCGTTTCACCGTGATTGGTGCGGGTCATGGTGGCAAGGGTATGGCGGCGCACCTGGCGCTGATG
GGTTTTCCGGTTACCCTGTACAACCGTACCCCGGATAACATCGCGGCGATTAAGGCGCGTGGTGGCATCGAGCTGAAAAGCT
TCGAAGGTGGCCCGCACGGTTTTGCGATGCTGGCGAAGGCGACCAGCGACATCGAGGAAGCGCTGAAAGAGTGCGATGTG
ATTATGGTGGTTGTTCCGAGCACCGCGCATGCGGAAATTGCGCGTAGCCTGGCGCCGCACCTGAAGGACGGTCAGATCATTG
TTCTGAACCCGGGTCGTACCTGCGGTGCGCTGGAAGTGAAACACACCCTGGAGGAAAACGGCTGCAGCGCGGATGTTACC
GTGGCGGAGGCGGAAACCCTGATCTATGCGAGCCGTAGCGATGGTCCGGCGCAAGCGCGTATCTTCGGCATTAAGGAAACC
GTGCCGCTGGCGGCGCTGCCGGCGACCCGTACCCAACTGGTTCTGGACACCCTGGCGCCGGCGTATCCGCAATTCATCGAT
GGTGGCAGCGTGCTGCACACCGGTCTGAACAACATGGGCGCGATTTTTCATCCGGCGCTGGCGCTGCTGAACGCGGGTCGT
ATCGAGAGCACCCAGGGCGAATTCCAATTTTATATTGACGGTGTTAGCCCGAGCGTTGCGCGTGTGCTGGAAGTGCTGGATC
GTGAACGTGTTACCGTGGCGAGCGCGCTGGGTATCCGTGCGCGTACCGCGCTGGAGTGGCTGCAGATGGCGTACCACGCGA
CCGGCAACGACCTGTATGAAGCGATCCACAACCAGCCGGGTTACTATGGCATTCAAGCTCCGCCGACCCTGAACCACCGTT
ACATCACCGAGGATGTGCCGATGAGCCTGGTTCCGATTGCGGCGCTGGGCGAGCGTTATGGCGTTAGCGTGCGTGGTATGGA
AAGCATCATTCGTCTGGCGTGCATCATTCACCGTACCGACTACTGGCGTCGTGGTCGTACCCTGGAAAAGCTGGGCATCCAC
GATTGGAGCGTTAGCGAGCTGACCAAATATGTGATGGAAGGCATGGTTTAA

>mODH-48 (Nucleotide sequence accession number in the TPA Section of the DDBJ/ENA/GenBank databases: BK063526)

Closest homologue in UniProt: A0A7X7W637 (100%)

MKIAVIGAGNGGQALAAVLAMRGNEVALYNRSQSRIQPILKSKKLKVEGESGGTARLKYVGTDMEKAVSDAELIMVVVPAFAH
AEVARKLSKFISEGQIIILNPGRTGGALEFDKILKECGVRNKAILAEAQTFLFASRISGPGMVRIFRIKNAVPVSVLPSKDNELLMPV
IGDVIPEFTLADNVLYTSFNNIGAIFHPGAIIMNAGWIESTHGDFQFYLDGISPAVARVLEEVDRERCEVTSKLGVQAMGAREWLD
YAYDARGEDLYNAIHNNEGYRGIMAPISMENRYIIEDVPMSLVPMSCFGKKLGVDTKTMDSVINIAGAIMGKDFWKEGRNIERL
GVKDMSVEDLWHYVEKGDGDG

ATGAAGATTGCGGTGATCGGTGCGGGTAACGGTGGCCAGGCGCTGGCGGCGGTGCTGGCGATGCGTGGTAACGAGGTTGCG
CTGTACAACCGTAGCCAGAGCCGTATTCAACCGATCCTGAAGAGCAAGAAACTGAAGGTGGAGGGTGAAAGCGGTGGCAC
CGCGCGTCTGAAGTATGTGGGTACCGACATGGAGAAAGCGGTTAGCGATGCGGAACTGATTATGGTGGTTGTTCCGGCGTTT
GCGCATGCGGAAGTGGCGCGTAAGCTGAGCAAATTTATCAGCGAAGGCCAGATCATTATTCTGAACCCGGGTCGTACCGGT
GGCGCGCTGGAGTTCGACAAGATTCTGAAAGAATGCGGCGTGCGTAACAAAGCGATCCTGGCGGAAGCGCAAACCTTCCT
GTTTGCGAGCCGTATCAGCGGTCCGGGCATGGTTCGTATTTTCCGTATCAAGAACGCGGTTCCGGTGAGCGTTCTGCCGAGC
AAAGATAACGAGCTGCTGATGCCGGTGATTGGTGACGTTATCCCGGAATTTACCCTGGCGGATAACGTGCTGTACACCAGCT
TCAACAACATTGGCGCGATCTTTCACCCGGGTGCGATTATCATGAACGCGGGCTGGATTGAGAGCACCCACGGTGACTTCCA
GTTTTATCTGGATGGTATTAGCCCGGCGGTGGCGCGTGTTCTGGAGGAAGTTGACCGTGAGCGTTGCGAAGTGACCAGCAA
GCTGGGCGTTCAAGCGATGGGTGCGCGTGAATGGCTGGACTACGCGTATGATGCGCGTGGTGAGGACCTGTACAACGCGAT
CCACAACAACGAAGGCTATCGTGGTATTATGGCGCCGATCAGCATGGAGAACCGTTACATTATCGAAGATGTGCCGATGAGC
CTGGTTCCGATGAGCTGCTTCGGCAAGAAACTGGGTGTGGACACCAAAACCATGGATAGCGTTATTAACATCGCGGGCGCG
ATTATGGGCAAGGATTTTTGGAAAGAGGGCCGTAACATCGAACGTCTGGGTGTGAAGGACATGAGCGTTGAGGATCTGTGG
CACTATGTTGAAAAAGGTGACGGCGATGGTTAA

>mODH-49 (Nucleotide sequence accession number in the TPA Section of the DDBJ/ENA/GenBank databases: BK063527)

Closest homologue in UniProt: A0A533SSB6 (91%)

MNHTPHFTVIGAGHGGKAMAAHLALMEFPTTLYNRTHDHILGVERRGGIELESYEGGPRGFGKLVKATSDMAEALKDADMIM
VVVPSSAHADVARSAAPHLKDGQIVVLHPGRTCGAIEFLKTLRDNNCTADVVVAETETFIYASRSDGPAQSRIFRIKEAVPLAALP
SKRTEEVLEMIHLPYPQFIDGGNVLQTGLNNMGAIFHPALTLLNSGWIEATHGDYQFYIDGVTPSVARVLEALDRERVTVAAALG
LRARTAMEWLKLAYDTTGEDLCEAIHNQPGYYGIKAPPTLNHRYIFEDVPMSLVPIASLGQRYGVSVRGMDSIIRLACIVHRTDY
WRRGRTIEKLGLGELSVTELTRFVIEGVLDPD

ATGAACCACACCCCGCACTTCACCGTGATTGGTGCGGGTCATGGTGGCAAAGCGATGGCGGCGCACCTGGCGCTGATGGAG
TTTCCGACCACCCTGTACAACCGTACCCACGACCACATCCTGGGTGTGGAGCGTCGTGGTGGCATTGAGCTGGAAAGCTAT
GAAGGTGGCCCGCGTGGTTTCGGCAAGCTGGTTAAAGCGACCAGCGATATGGCGGAAGCGCTGAAAGACGCGGATATGATC
ATGGTGGTTGTTCCGAGCAGCGCGCATGCGGATGTTGCGCGTAGCGCGGCGCCGCACCTGAAGGATGGTCAGATCGTTGTT
CTGCACCCGGGTCGTACCTGCGGTGCGATTGAGTTTCTGAAAACCCTGCGTGACAACAACTGCACCGCGGATGTTGTGGTT
GCGGAGACCGAAACCTTCATTTATGCGAGCCGTAGCGATGGTCCGGCGCAAAGCCGTATCTTTCGTATTAAAGAGGCGGTTC
CGCTGGCGGCGCTGCCGAGCAAACGTACCGAGGAAGTTCTGGAAATGATCCACCTGCCGTATCCGCAGTTCATTGATGGTG
GCAACGTGCTGCAAACCGGTCTGAACAACATGGGCGCGATCTTTCACCCGGCGCTGACCCTGCTGAACAGCGGTTGGATCG
AAGCGACCCACGGCGACTACCAGTTCTATATTGATGGTGTTACCCCGAGCGTGGCGCGTGTTCTGGAGGCGCTGGACCGTG
AACGTGTGACCGTTGCGGCGGCGCTGGGTCTGCGTGCGCGTACCGCGATGGAGTGGCTGAAGCTGGCGTACGACACCACC
GGCGAGGATCTGTGCGAAGCGATCCACAACCAGCCGGGTTACTATGGCATTAAAGCTCCGCCGACCCTGAACCACCGTTAC
ATCTTCGAAGATGTGCCGATGAGCCTGGTTCCGATTGCGAGCCTGGGTCAACGTTATGGCGTGAGCGTTCGTGGTATGGACA
GCATCATTCGTCTGGCGTGCATCGTGCACCGTACCGATTATTGGCGTCGTGGCCGTACCATTGAGAAGCTGGGTCTGGGCGA
GCTGAGCGTTACCGAACTGACCCGTTTTGTGATCGAAGGTGTTCTGGACCCGGATTAA

>mODH-50 (Nucleotide sequence accession number in the TPA Section of the DDBJ/ENA/GenBank databases: BK063528)

Closest homologue in UniProt: A0A973EU57 (100%)

MPNNMIKSKNKIAVLGAGHGGLAMAGHLALMGHNVNLFNRGEERLWGVKSSGAIEITGEVEGFGKINMATTSIKDAIEGVELI
MVVVPASGHRWIAEQIAPYLVDGQIIVLHPGRTFGALEFKNILIQKQVKADVIISEAQTFIYASRATGPSQVHIFRIKHSIPVASVRA
HLIPKVINKLRQFYPQFVPGDNIFKTSLENIGSVFHPALCVLNAGWIEHDIDYQFYHEGATPSVARVLEKIDEERVNVSEALGIRAIT
ARQWLYMAYSATGDTLFEAMRKNAGYRGILAPRTLKMRYLEEDIPCSLVPIASVGNMLGVETPTINSIINLACQLNGTNYWAIGR
TVENLGIAGMSLRELRLLAIGESPKQ

ATGCCGAACAACATGATCAAGAGCAAAAACAAGATTGCGGTTCTGGGTGCGGGTCATGGTGGCCTGGCGATGGCGGGTCAC
CTGGCGCTGATGGGCCACAACGTGAACCTGTTCAACCGTGGTGAGGAACGTCTGTGGGGTGTTAAAAGCAGCGGCGCGAT
CGAGATTACCGGCGAGGTGGAAGGTTTTGGCAAAATCAACATGGCGACCACCAGCATCAAGGACGCGATTGAGGGTGTTG
AACTGATTATGGTGGTTGTTCCGGCGAGCGGTCACCGTTGGATTGCGGAACAGATTGCGCCGTACCTGGTTGACGGTCAAAT
CATTGTTCTGCACCCGGGTCGTACCTTTGGTGCGCTGGAGTTTAAGAACATCCTGATTCAGAAACAAGTTAAGGCGGATGTG
ATCATTAGCGAAGCGCAGACCTTCATTTATGCGAGCCGTGCGACCGGTCCGAGCCAAGTGCACATCTTTCGTATTAAACACA
GCATTCCGGTTGCGAGCGTGCGTGCGCACCTGATCCCGAAAGTTATTAACAAGCTGCGTCAGTTCTATCCGCAATTTGTGCC
GGGTGACAACATCTTCAAGACCAGCCTGGAGAACATTGGCAGCGTTTTTCATCCGGCGCTGTGCGTGCTGAACGCGGGTTG
GATCGAGCACGACATTGATTACCAGTTCTATCACGAAGGTGCGACCCCGAGCGTTGCGCGTGTGCTGGAGAAAATCGATGA
GGAACGTGTTAACGTGAGCGAAGCGCTGGGTATCCGTGCGATTACCGCGCGTCAATGGCTGTACATGGCGTATAGCGCGACC
GGCGACACCCTGTTTGAAGCGATGCGTAAAAACGCGGGTTACCGTGGCATCCTGGCGCCGCGTACCCTGAAGATGCGTTAT

CTGGAGGAAGATATCCCGTGCAGCCTGGTTCCGATTGCGAGCGTGGGTAACATGCTGGGCGTTGAGACCCCGACCATCAAC
AGCATCATTAACCTGGCGTGCCAGCTGAACGGTACCAACTATTGGGCGATCGGCCGTACCGTGGAAAACCTGGGTATTGCGG
GCATGAGCCTGCGTGAGCTGCGTCTGCTGGCGATCGGTGAAAGCCCGAAGCAATAA

>mODH-52 (Nucleotide sequence accession number in the TPA Section of the DDBJ/ENA/GenBank databases: BK063529)
Closest homologue in UniProt: A0A6L9J0X1 (78%)

MAADLAVRGYRVCLYNRSYENIAAIDARHGITIDLEDGRQAFGRLHRATSDMAQALAGSQLIMVVVPASAHRDIALACAPHLQD
GQIVVLNPGRTGGALEFRQVLAEQNCTADVTIAEAETFIFASRSMGPAEARIFRRKDAVPLAALPATRTAQVLDLVQEIYPSFIAAP
NVLYTSLNNMGAIFHPALTLLNAGWIEATGGEFEFYIDGVTPSTARLLERLDRERVTVATAMGVRAQSAQEWLARAYSAHGEDL
YEAIHDNPGYKGINAPSTLRHRYIFEDVPYSMVPIAELGRRFGVDVWGMEAMIQLACVLHGTDYRYRGRTLARMGLEGLSLTEI
TNLVNTGRAEANHRSSD

ATGGCGGCGGATCTGGCGGTTCGTGGTTACCGTGTGTGCCTGTACAACCGTAGCTATGAGAACATCGCGGCGATTGACGCGC
GTCACGGCATCACCATTGACCTGGAAGATGGTCGTCAAGCGTTTGGCCGTCTGCACCGTGCGACCAGCGATATGGCGCAGG
CGCTGGCGGGTAGCCAACTGATTATGGTGGTTGTTCCGGCGAGCGCGCACCGTGACATTGCGCTGGCGTGCGCGCCGCACC
TGCAGGATGGTCAAATCGTTGTTCTGAACCCGGGTCGTACCGGTGGCGCGCTGGAGTTCCGTCAGGTTCTGGCGGAACAAA
ACTGCACCGCGGATGTGACCATTGCGGAGGCGGAAACCTTCATTTTTGCGAGCCGTAGCATGGGTCCGGCGGAGGCGCGTA
TCTTTCGTCGTAAAGATGCGGTTCCGCTGGCGGCGCTGCCGGCGACCCGTACCGCGCAGGTTCTGGATCTGGTGCAAGAAA
TCTACCCGAGCTTCATTGCGGCGCCGAACGTGCTGTATACCAGCCTGAACAACATGGGTGCGATTTTTCATCCGGCGCTGAC
CCTGCTGAACGCGGGTTGGATTGAGGCGACCGGTGGCGAGTTCGAATTTTACATTGACGGTGTTACCCCGAGCACCGCGCG
TCTGCTGGAGCGTCTGGATCGTGAACGTGTTACCGTGGCGACCGCGATGGGTGTGCGTGCGCAGAGCGCGCAAGAATGGCT
GGCGCGTGCGTACAGCGCGCATGGCGAGGACCTGTATGAAGCGATCCACGATAACCCGGGTTATAAAGGCATTAACGCGCC
GAGCACCCTGCGTCACCGTTACATCTTCGAGGACGTTCCGTATAGCATGGTGCCGATTGCGGAACTGGGTCGTCGTTTTGGC
GTTGATGTGTGGGGTATGGAGGCGATGATCCAACTGGCGTGCGTTCTGCACGGTACCGACTACCGTTATCGTGGCCGTACCC
TGGCGCGTATGGGTCTGGAGGGCCTGAGCCTGACCGAAATTACCAACCTGGTGAACACCGGCCGTGCGGAAGCGAACCAC
CGTAGCAGCGATTAA

>mODH-55 (Nucleotide sequence accession number in the TPA Section of the DDBJ/ENA/GenBank databases: BK063530)
Closest homologue in UniProt: A0A7C4P2H9 (96%)

MTTPERYTVIGAGHGGKAMAAHLSLMGFKVTLFNRTPDHIAVLKKRGGIELEAPEGAPHGFGKLAKATSDYGEAVKNAQVIMV
VVPSSAHADVAKGVARHLKKGQIILLHPGRTCGAIEFAKVLRDEGCKADVTIAEAETFIYASRSDGPAEARIFRVKEAVPLAALPAT
RTEQVLKAIEPAYPQYIDGGNVLQTGLNNMGAIFHPALTILNSGWIEATHGDYQFYIDGVTPSVARVLEALDRERVTVASSLGIRA
RTALEWLKLAYDTTGEDLHEAIHNQPGYYGIKAPPTLNHRYIFEDVPMSLVPIAALGQRYGVSVRGMDSIIRLASIIHRTDYWRRG
RTLEKLGIDNLSVGELTRYVTEGVLET

ATGACCACCCCGGAGCGTTACACCGTTATTGGCGCGGGCCACGGCGGCAAAGCGATGGCGGCGCACCTGAGCCTGATGGGT
TTCAAGGTTACCCTGTTCAACCGTACCCCGGACCACATCGCGGTGCTGAAGAAACGTGGTGGCATTGAACTGGAAGCGCCG
GAGGGTGCGCCGCACGGTTTTGGCAAGCTGGCGAAAGCGACCAGCGACTACGGCGAAGCGGTTAAGAACGCGCAGGTGAT
CATGGTGGTTGTTCCGAGCAGCGCGCATGCGGATGTTGCGAAAGGTGTGGCGCGTCACCTGAAGAAAGGCCAAATCATTCT
GCTGCACCCGGGTCGTACCTGCGGTGCGATTGAGTTCGCGAAGGTTCTGCGTGACGAAGGTTGCAAAGCGGATGTGACCAT
CGCGGAGGCGGAAACCTTCATTTATGCGAGCCGTAGCGATGGTCCGGCGGAGGCGCGTATCTTTCGTGTTAAGGAAGCGGT

GCCGCTGGCGGCGCTGCCGGCGACCCGTACCGAGCAGGTTCTGAAAGCGATCGAACCGGCGTACCCGCAGTATATTGATGG
TGGCAACGTGCTGCAAACCGGTCTGAACAACATGGGCGCGATCTTCCACCCGGCGCTGACCATCCTGAACAGCGGTTGGAT
TGAGGCGACCCACGGCGACTACCAATTTTATATTGATGGTGTTACCCCGAGCGTTGCGCGTGTGCTGGAGGCGCTGGACCGT
GAACGTGTTACCGTGGCGAGCAGCCTGGGTATTCGTGCGCGTACCGCGCTGGAGTGGCTGAAGCTGGCGTACGACACCACC
GGCGAGGATCTGCACGAAGCGATCCACAACCAGCCGGGTTACTATGGCATTAAAGCTCCGCCGACCCTGAACCACCGTTAC
ATCTTTGAAGATGTTCCGATGAGCCTGGTGCCGATTGCGGCGCTGGGTCAACGTTATGGCGTTAGCGTGCGTGGTATGGACA
GCATCATTCGTCTGGCGAGCATCATTCACCGTACCGATTATTGGCGTCGTGGTCGTACCCTGGAAAAGCTGGGCATCGACAAT
CTGAGCGTGGGCGAACTGACCCGTTATGTGACCGAAGGCGTGCTGGAGACCTAA

>mODH-581 (Nucleotide sequence accession number in the TPA Section of the DDBJ/ENA/GenBank databases: BK063531)

Closest homologue in UniProt: A0A937NJY1 (62%)

MDAGKPKILVIGAGHGGKAMAADLAIKGFPVRLYNRTYSRIEMIALRGGIDLEFEDGRSAFGPLEMVTSDLGMALEGVNLVMV
VVPASAHADIAAQCAPLLEDGQVIILNPGRTGGALEFRRVLRENGCRASVVVAEAQTFVLSSRSTGPAEARLFRSKITVPLAALPS
TDTPRALEMARLAYPQFIPARNVLETSLDNMGAVMHPSLMILNAGRIEATRGEFQFYIDGATPGVATVLEQVDAERVAVARALQV
RTRSALEWLRAAYASVGDDLFHAIQGTPTYRGVAAPRSLMHRYLFEDVPMSLVPIATIGAQYGVETPTIRAVIQLACVLHRKDYF
ALGRTPARLGIGGMSVEQLTRYVEGR

ATGGACGCGGGCAAGCCGAAAATCCTGGTGATTGGTGCGGGTCATGGTGGCAAGGCGATGGCGGCGGATCTGGCGATCAAA
GGCTTCCCGGTTCGTCTGTACAACCGTACCTATAGCCGTATCGAGATGATTGCGCTGCGTGGTGGCATTGACCTGGAGTTCGA
AGATGGTCGTAGCGCGTTTGGCCCGCTGGAGATGGTGACCAGCGACCTGGGTATGGCGCTGGAAGGCGTGAACCTGGTTAT
GGTGGTTGTTCCGGCGAGCGCGCATGCGGACATTGCGGCGCAGTGCGCGCCGCTGCTGGAAGATGGTCAAGTGATCATTCT
GAACCCGGGTCGTACCGGTGGCGCGCTGGAGTTCCGTCGTGTTCTGCGTGAAAACGGTTGCCGTGCGAGCGTTGTGGTTGC
GGAGGCGCAGACCTTCGTGCTGAGCAGCCGTAGCACCGGTCCGGCGGAAGCGCGTCTGTTTCGTAGCAAGATCACCGTTCC
GCTGGCGGCGCTGCCGAGCACCGACACCCCGCGTGCGCTGGAGATGGCGCGTCTGGCGTACCCGCAGTTTATTCCGGCGCG
TAACGTGCTGGAAACCAGCCTGGATAACATGGGTGCGGTTATGCACCCGAGCCTGATGATCCTGAACGCGGGTCGTATTGAG
GCGACCCGTGGCGAATTCCAATTTTACATTGATGGTGCGACCCCGGGCGTGGCGACCGTTCTGGAGCAGGTGGATGCGGAA
CGTGTGGCGGTTGCGCGTGCGCTGCAAGTTCGTACCCGTAGCGCGCTGGAATGGCTGCGTGCGGCGTATGCGAGCGTGGGT
GACGACCTGTTCCACGCGATTCAGGGTACCCCGACCTACCGTGGCGTTGCGGCGCCGCGTAGCCTGATGCACCGTTACCTGT
TCGAGGACGTGCCGATGAGCCTGGTTCCGATCGCGACCATTGGTGCGCAGTACGGCGTTGAAACCCCGACCATCCGTGCGG
TGATTCAACTGGCGTGCGTTCTGCACCGTAAAGATTATTTTGCGCTGGGTCGTACCCCGGCGCGTCTGGGTATCGGTGGCAT
GAGCGTGGAGCAACTGACCCGTTATGTTGAAGGTCGTTAA

>mODH-582 (Nucleotide sequence accession number in the TPA Section of the DDBJ/ENA/GenBank databases: BK063532)

Closest homologue in UniProt: A0A7V4ZEF3 (94%)

MTQPVKVAIVGGGNGAHAMAGHLALKGHTVRLYSAFPQEIRAMQEAGGVFVEGAVEGFGRLDDVSADPAAVIPWAEVIMVVV
PAFAHRPLAETLAPHLRDGQVLLLNPGRTGGALEVAAVLKRLGVTADVVVGEAQTLIYACRISGPARVRVLGLKRTVPVAALPAT
QNDRLLSQVTRLFPEFCAAQNVLETSLDNIGAVFHPATMVLNANRIEAGEDFDFYQGMTPMVTKTLEAVDEERLAVARAYGVAT
ESAADWLLKAYDGVRGNTLYERIQSNRAYAGIKAPKALNVRYITEDVPTGLVPIAAFGRAAGLTMRTCEGIIDLCCTLLGRDFWA
EGRNLENLGLAGMDREAILRYVNESASA

ATGACCCAGCCGGTGAAGGTTGCGATCGTGGGTGGCGGTAACGGTGCGCATGCGATGGCGGGTCACCTGGCGCTGAAAGG
CCACACCGTTCGTCTGTATAGCGCGTTCCCGCAGGAGATTCGTGCGATGCAAGAAGCGGGCGGTGTGTTCGTTGAGGGTGC
GGTGGAAGGCTTTGGTCGTCTGGATGATGTTAGCGCGGACCCGGCGGCGGTGATCCCGTGGGCGGAAGTGATTATGGTGGT
TGTTCCGGCGTTTGCGCATCGTCCGCTGGCGGAAACCCTGGCGCCGCACCTGCGTGATGGTCAGGTTCTGCTGCTGAACCC
GGGTCGTACCGGCGGTGCGCTGGAAGTGGCGGCGGTTCTGAAGCGTCTGGGTGTTACCGCGGATGTTGTGGTTGGCGAAGC
GCAAACCCTGATCTACGCGTGCCGTATTAGCGGTCCGGCGCGTGTGCGTGTTCTGGGTCTGAAACGTACCGTGCCGGTTGCG
GCGCTGCCGGCGACCCAGAACGACCGTCTGCTGAGCCAAGTGACCCGTCTGTTCCCGGAGTTTTGCGCGGCGCAGAACGT
TCTGGAAACCAGCCTGGATAACATCGGTGCGGTGTTCCACCCGGCGACCATGGTTCTGAACGCGAACCGTATTGAGGCGGG
TGAAGACTTCGATTTTTATCAAGGCATGACCCCGATGGTGACCAAGACCCTGGAGGCGGTTGATGAGGAACGTCTGGCGGT
GGCGCGTGCGTACGGCGTTGCGACCGAAAGCGCGGCGGACTGGCTGCTGAAAGCGTACGATGGCGTGCGTGGTAACACCC
TGTATGAGCGTATCCAAAGCAACCGTGCGTACGCGGGTATTAAGGCGCCGAAAGCGCTGAACGTTCGTTATATCACCGAAGA
TGTGCCGACCGGTCTGGTTCCGATTGCGGCGTTTGGTCGTGCGGCGGGTCTGACCATGCGTACCTGCGAGGGTATCATTGAC
CTGTGCTGCACCCTGCTGGGTCGTGATTTTTGGGCGGAGGGCCGTAACCTGGAAAACCTGGGCCTGGCGGGTATGGATCGT
GAGGCGATCCTGCGTTACGTGAACGAAAGCGCGAGCGCGTAA

Table S3 - Conversion data with all amino acid substrates.[a,b]

Name	Structure	*Ar*ODH	mODH-45	mODH-47	mODH-48	mODH-49	mODH-55	mODH-582
L-Phenylalanine (**1**)	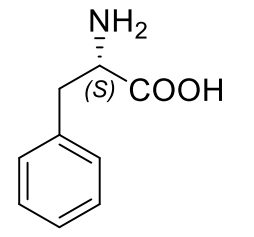	89.9±0.4	6.1±2.6	4.0±1.6	2.7±2.3	8.8±2.0	4.4±3.6	7.2±2.1
L-Histidine (**2**)	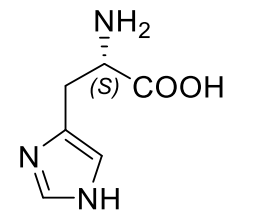	37.8±3.0	3.0±5.6	11.4±4.3	17.8±4.5	26.1±3.5	21.2±5.1	28.6±3.5
L-Aspartate (**3**)	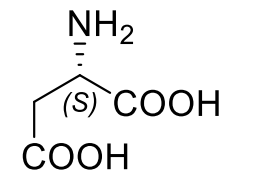	7.0±3.8	97.4±2.5	94.4±1.0	75.9±1.4	99.6±0.0	37.8±2.4	97.2±0.8
L-Glutamate (**4**)	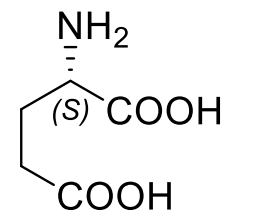	5.8±0.9	35.0±11.7	57.2±9.5	2.1±1.2	47.7±1.8	54.0±3.3	96.4±0.6
phospho-L-serine (**5**)	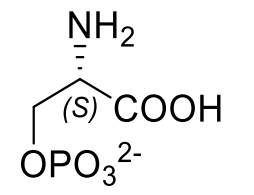	0.0±2.9	17.9±4.5	13.4±4.2	10.7±2.8	15.4±2.3	5.7±2.9	0.8±5.5
phospho-L-threonine (**6**)	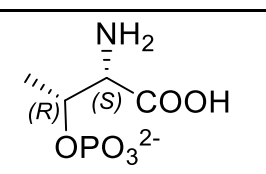	0.0±0.9	33.6±3.5	12.7±4.3	13.9±5.9	13.7±2.0	2.9±7.3	3.1±1.7
L-cysteic acid (**7**)	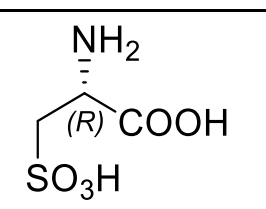	0.0±1.0	97.6±0.2	99.3±1.0	71.1±4.1	99.3±0.2	66.3±0.7	98.2±0.0
4-carboxy-L-phenyalanine (**8**)	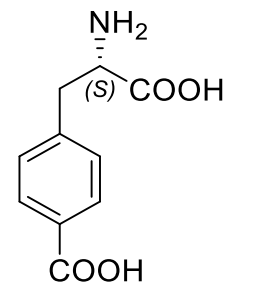	0.9±1.9	9.3±5.8	5.6±2.1	37.2±4.2	0.7±2.9	0.6±4.0	0.0±1.2
Homocysteic acid (**9**)	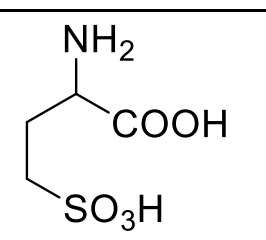	0.0±3.9	41.0±2.1	41.2±1.4	41.0±1.5	40.6±1.5	33.5±4.9	39.4±1.7
2-aminopimelic acid (**10**)	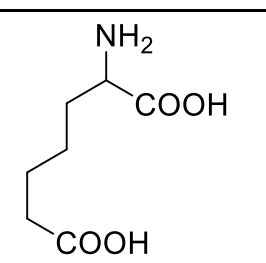	0.0±1.3	43.6±1.0	43.8±1.4	42.7±0.9	42.3±2.6	34.3±3.1	29.8±1.7
Homoserine (**11**)	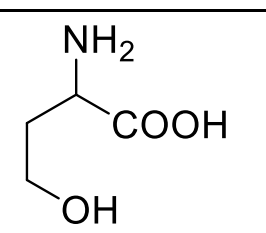	53.0±3.3	42.3±2.8	18.3±4.6	31.1±6.1	35.6±3.4	15.0±6.2	6.8±9.0
Trifluoro-homoalanine (**12**)	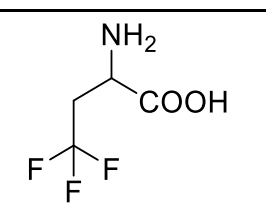	84.5±2.4	36.4±3.7	9.6±5.6	8.2±5.7	16.9±5.0	2.4±7.7	12.0±8.0
L-Serine (**13**)	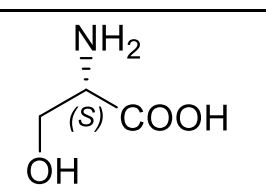	99.3±0.0	12.3±9.2	5.0±6.0	1.6±2.5	3.5±3.0	9.7±10.3	10.8±12.5
L-Threonine (**14**)	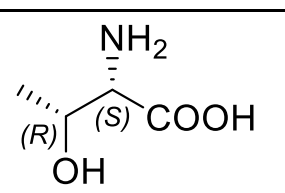	100.0±0.0	14.6±10.3	4.2±2.7	4.9±3.5	3.3±1.0	16.5±4.7	11.6±2.6
Glycine (**15**)	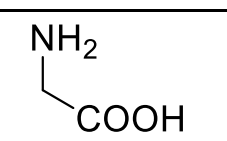	100.0±0.0	23.9±4.1	20.2±7.0	41.0±3.6	30.2±2.3	8.4±3.7	15.6±1.5
L-Alanine (**16**)	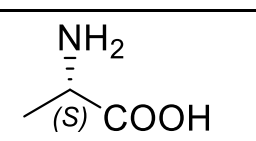	100.0±0.0	15.6±3.3	3.5±3.7	18.9±4.1	13.8±3.1	9.0±1.1	6.4±0.9
L-Valine (**17**)	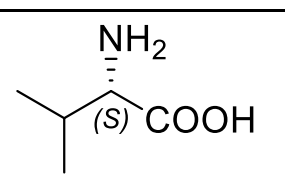	98.0±0.1	5.7±4.7	1.5±5.8	13.1±6.5	6.3±3.0	1.7±3.1	16.2±3.3

L-Leucine (**18**)	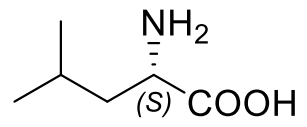	100.0±0.0	14.8±2.1	13.1±3.9	8.9±6.2	19.3±3.3	7.9±2.9	10.8±4.0
L-Isoleucine (**19**)	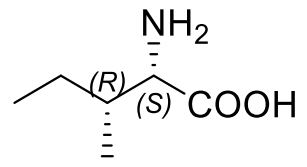	96.5±0.2	0.0±4.4	15.0±4.5	16.8±4.3	13.7±4.5	0.0±5.7	21.5±4.4
L-Methionine (**20**)	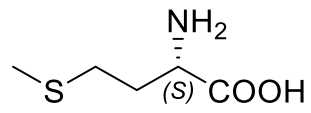	100.0±0.0	15.5±4.6	15.9±5.6	8.5±5.3	20.9±4.1	9.4±4.2	6.2±4.9
L-Arginine (**21**)	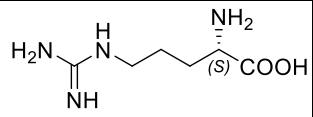	0.0±4.2	5.4±4.8	2.2±4.5	0.0±5.9	12.6±7.5	12.2±4.4	2.3±7.8
L-threo-phenylserine (**22**)	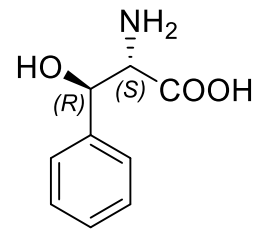	100.0±0.0	8.1±2.8	6.8±3.7	1.7±4.7	5.1±3.3	0.5±5.6	2.5±6.0
Dimethylamino-alanine (**23**)	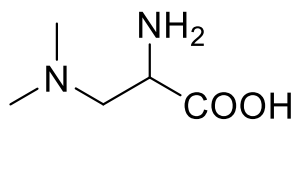	4.8±19.7	19.3±13.5	4.9±22.2	0.0±13.8	14.4±12.2	12.1±10.9	0.0±19.1
Dimethylamino-phenylalanine (**24**)	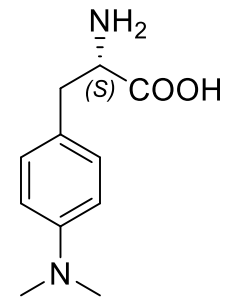	46.1±1.6	7.6±3.8	6.0±5.1	5.2±6.9	4.9±3.6	6.4±2.1	0.0±2.0
β-alanine (**25**)	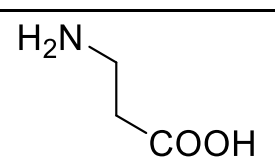	100.0±0.0	13.1±13.0[c]	33.7±2.2[c]	30.7±1.6[c]	30.5±2.5[c]	30.2±7.6[c]	21.3±12.3[c]
Taurine (**26**)	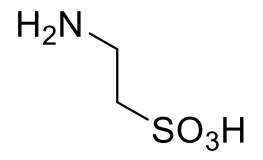	0.0±6.4	10.5±6.7	18.7±5.6	11.8±10.8	9.5±10.8	0.7±7.3	0.0±10.8

[a] Standard errors are calculated according to the Gaussian error propagation rules: $D\sqrt{\left(\frac{\sigma_A}{A}\right)^2+\left(\frac{\sigma_{bl}}{A_{bl}}\right)^2}$, where D is the % of amino acid depletion, A is the average area under the chromatographic peak of the amino acid substrate, σ_A is its standard deviation, A_{bl} is the same average area in a blank reaction (without the addition of the enzyme), σ_{bl} is its standard deviation.

[b] The conversion was calculated as described in Materials and Methods.

[c] No product formation was observed by HPLC-MS despite apparent conversions.

Table S4 - Conversion data with all ketoacid substrates.[a,b]

		*Ar*ODH	mODH-45	mODH-47	mODH-48	mODH-49	mODH-55	mODH-582
Pyruvate (**a**)	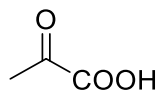	7.0±3.8	97.4±2.5	94.4±1.0	75.9±1.4	99.6±0.0	37.8±2.4	97.2±0.8
Glyoxylate (**b**)	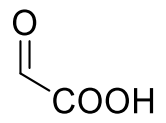	0.0±8.0	3.3±7.5	11.8±7.1	2.3±6.7	4.4±6.9	0.0±10.6	5.2±8.1
α-Ketobutyrate (**c**)	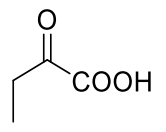	1.3±4.5	14.9±1.5	8.4±1.6	13.0±2.8	16.8±1.9	9.0±2.0	21.6±1.3
α-Ketoglutarate (**d**)	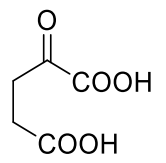	0.3±5.6	8.3±3.1	15.8±5.8	2.1±5.2	9.9±4.6	7.8±3.1	5.7±3.3
α-Ketoglutarate (**d**) [c]	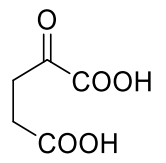	0.0±14.6	5.2±8.8	2.0±9.6	0.6±10.2	6.1±8.4	0.0±11.3	0.0±11.3
α-Ketovalerate (**e**)	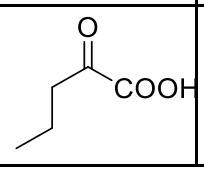	0.0±4.5	22.9±5.1	6.4±4.3	5.5±5.3	11.0±3.9	11.6±5.9	0.0±8.9

[a] Standard errors are calculated according to the Gaussian error propagation rules: $D\sqrt{\left(\frac{\sigma_A}{A}\right)^2+\left(\frac{\sigma_{bl}}{A_{bl}}\right)^2}$, where D is the % of amino acid depletion, A is the average area under the chromatographic peak of the amino acid substrate, σ_A is its standard deviation, A_{bl} is the same average area in a blank reaction (without the addition of the enzyme), σ_{bl} is its standard deviation.
[b] The conversion was calculated as described in Materials and Methods.
[c] L-Alanine substrate was used instead of L-aspartate

Table S5 – Comparison of key active site residues of ODHs, that were mutated by Codexis when engineering ArODH

ArODH	A111	S136	K156	V197	N198	M201	Y259	Y280	R292	Y293
**mODH-582**	R	Q	K	I	G	F	L	Y	R	Y
**mODH-48**	R	Q	K	I	G	F	L	Y	R	Y
**mODH-47**	R	E	K	M	G	F	L	Y	R	Y
**mODH-45**	R	E	K	M	G	F	L	Y	R	Y
**mODH-49**	R	E	K	M	G	F	L	Y	R	Y
**mODH-55**	R	E	K	M	G	F	L	Y	R	Y

Table S6 – Primers used for mutagenesis

*Ar*ODH A111R

Name	Oligo (Uppercase = target-specific primer)	Len	%GC	Tm	Ta *
pET14b-ArODH_A111R_fw	GAACCCGGGTcgtACCGGTGGCG	23	74	62°C	62°C
pET14b-ArODH_A111R_rev	AGAATGATCAGCTGACCC	18	50	61°C	

* Ta (recommended annealing temperature)

mODH-582 R110A

Name	Oligo (Uppercase = target-specific primer)	Len	%GC	Tm	Ta *
pET14b-mODH-582_R110A_fw	GAACCCGGGTgcgACCGGCGGTG	23	78	63°C	64°C
pET14b-mODH-582_R110A_fw	AGCAGCAGAACCTGACCA	18	56	66°C	

* Ta (recommended annealing temperature)

*Ar*ODH A111R N198G

Name	Oligo (Uppercase = target-specific primer)	Len	%GC	Tm	Ta *
pET14b-ArODH_N198G_fw	GACCAACGTGGGCGCGGTTATGC	23	65	64°C	63°C
pET14b-ArODH_N198G_rev	AGGCTGGTGTGCAG	14	64	62°C	

* Ta (recommended annealing temperature)

mODH-582 R110A G198N

Name	Oligo (Uppercase = target-specific primer)	Len	%GC	Tm	Ta *
pET14b-mODH-582_R110A_fw	GGATAACATCAACGCGGTGTTCCAC	25	52	58°C	58°C
pET14b-mODH-582_R110A_fw	AGGCTGGTTTCCAG	14	57	57°C	

* Ta (recommended annealing temperature)

Table S7 – Conversions obtained with mutant and wild type enzymes. Reaction conditions are the same as described for the screening.

	*Ar*ODH wt	*Ar*ODH A111R	mODH-582 wt	mODH-582 R110A
L-Aspartate	<5%	<5%	90%	<5%
L-Phenylalanine	>99%	<1%	<1%	<1%

Table S8 – Conversion data for cofactor preference study of mODHs with L-aspartate.[a,b]

Cofactor	mODH-45	mODH-47	mODH-48	mODH-49	mODH-55	mODH-582
NADH (2h)	16.0±8.4	n.d.	56.7±3.5	70.2±3.2	45.6±33.3	73.1±3.4
NADPH (2h)	28.6±2.1	n.d.	15.1±3.7	17.4±2.5	4.5±2.5	49.7±5.2
NADH (24h)	54.9±1.8	4.2±2.1	99.5±0.1	99.1±0.0	54.5±7.9	99.1±0.3
NADPH (24h)	94.7±1.4	76.7±4.6	75.1±10.3	99.6±0.3	39.0±5.5	99.4±0.5

[a] Standard errors are calculated according to the Gaussian error propagation rules: $D\sqrt{\left(\frac{\sigma_A}{A}\right)^2 + \left(\frac{\sigma_{bl}}{A_{bl}}\right)^2}$, where D is the % of amino acid depletion, A is the average area under the chromatographic peak of the amino acid substrate, σ_A is its standard deviation, A_{bl} is the same average area in a blank reaction (without the addition of the enzyme), σ_{bl} is its standard deviation.

[b] The conversion was calculated as described in Materials and Methods.

Table S9 – Kinetic constants of ODHs with L-aspartate. The corresponding Michaelis-Menten curves can be seen on Figure S15.

Enzyme	c [µM]	cofactor	V_{max} [µmol/min]	K_M [mM]	k_{cat} [1/s]
*Ar*ODH	0.015	NADH	no reaction observed		
*Ar*ODH A111R N198G	1.5	NADH	0.025 ± 0.006	58.3 ± 19.6	2.80 ± 0.69
mODH-45	0.71	NADPH	0.0032 ± 0.0005	21.5 ± 5.3	0.76 ± 0.11
mODH-47	0.72	NADPH	0.0005 ± 0.00003	1.2 ± 0.3	0.11 ± 0.01
mODH-48	0.72	NADPH	0.0030 ± 0.00009	0.5 ± 0.1	0.70 ± 0.02
mODH-49	0.70	NADPH	0.0039 ± 0.0003	15.0 ± 2.3	0.93 ± 0.07
mODH-55	0.71	NADPH	0.0066 ± 0.0002	1.5 ± 0.2	1.55 ± 0.05
mODH-582	0.73	NADH	0.058 ± 0.006	1.0 ± 0.5	13.1 ± 1.43
mODH-582	0.73	NADPH	0.0039 ± 0.0004	24.8 ± 4.6	0.89 ± 0.10

Table S10 – Kinetic constants of ODHs with L-phenylalanine. The corresponding Michaelis-Menten curves can be seen on Figure S16.

Enzyme	c [µM]	cofactor	V_{max} [µmol/min]	K_M [mM]	k_{cat} [1/s]
*Ar*ODH	0.015	NADH	0.077 ± 0.006	1.4 ± 0.5	861 ± 70
*Ar*ODH	0.015	NADPH	0.001 ± 0.0001	18.1 ± 4.5	11.1 ± 1.5
mODH-582	0.73	NADPH	no reaction observed		
mODH-582 R110A G198N	1.5	NADH	0.041 ± 0.009	n.d.	4.6 ± 1.0
mODH-582 R110A G198N	1.5	NADPH	0.015 ± 0.001	32.7 ± 4.7	1.7 ± 0.1

Table S11 – Kinetic constants of ODHs with NADPH. The corresponding Michaelis-Menten curves can be seen on Figure S17.

Enzyme	c [µM]	substrate	V_{max} [µmol/min]	K_M [µM]	k_{cat} [1/s]
ArODH	0.015	Phe	Non-Michaelis-Menten kinetics		
ArODH A111R N198G	1.5	Asp	0.0007 ± 0.0004	890 ± 636	0.08 ± 0.04
mODH-45	0.71	Asp	0.0042 ± 0.0003	118 ± 24	1.00 ± 0.08
mODH-47	0.72	Asp	0.0014 ± 0.0001	93 ± 21	0.33 ± 0.03
mODH-48	0.72	Asp	0.0033 ± 0.0002	17 ± 7	0.76 ± 0.04
mODH-49	0.70	Asp	0.0052 ± 0.0004	85 ± 17	1.23 ± 0.09
mODH-55	0.71	Asp	0.0077 ± 0.0005	68 ± 13	1.80 ± 0.11
mODH-582	0.73	Asp	0.0027 ± 0.0006	318 ± 112	0.62 ± 0.14
mODH-582 R110A G198N	1.5	Phe	Non-Michaelis-Menten kinetics		

Table S12 – Kinetic constants of ODHs with NADH. The corresponding Michaelis-Menten curves can be seen on Figure S18.

Enzyme	c [µM]	substrate	V_{max} [µmol/min]	K_M [µM]	k_{cat} [1/s]
ArODH	0.015	Phe	0.11 ± 0.02	292 ± 77	1240 ± 174
ArODH A111R N198G	1.5	Asp	0.0067 ± 0.0003	220 ± 18	0.75 ± 0.03
mODH-45	0.71	Asp	0.0038 ± 0.0003	447 ± 64	0.90 ± 0.08
mODH-47	0.72	Asp	no reaction observed		
mODH-48	0.72	Asp	0.019 ± 0.001	93 ± 15	4.36 ± 0.25
mODH-49	0.70	Asp	0.021 ± 0.004	1828 ± 423	4.95 ± 0.95
mODH-55	0.71	Asp	0.0021 ± 0.0002	442 ± 63	0.50 ± 0.04
mODH-582	0.73	Asp	0.016 ± 0.001	29 ± 11	18.1 ± 1.4

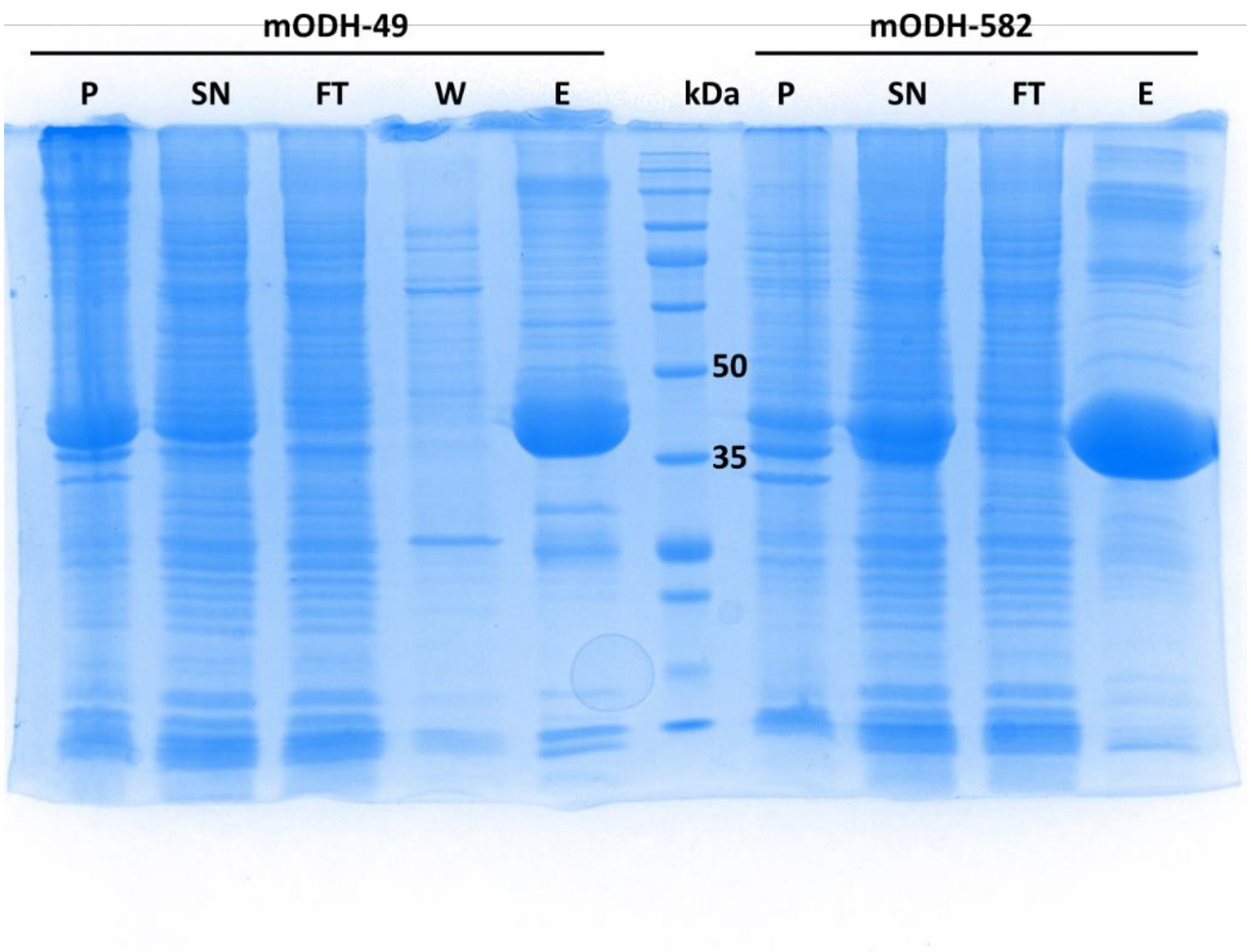

Figure S1 – Representative SDS-PAGE analysis of the Ni-affinity purification of mODHs.
P=pellet, SN=supernatant, FT=flowthrough, W=wash, E=elution; 5 mL sample was loaded for qualitative analysis.

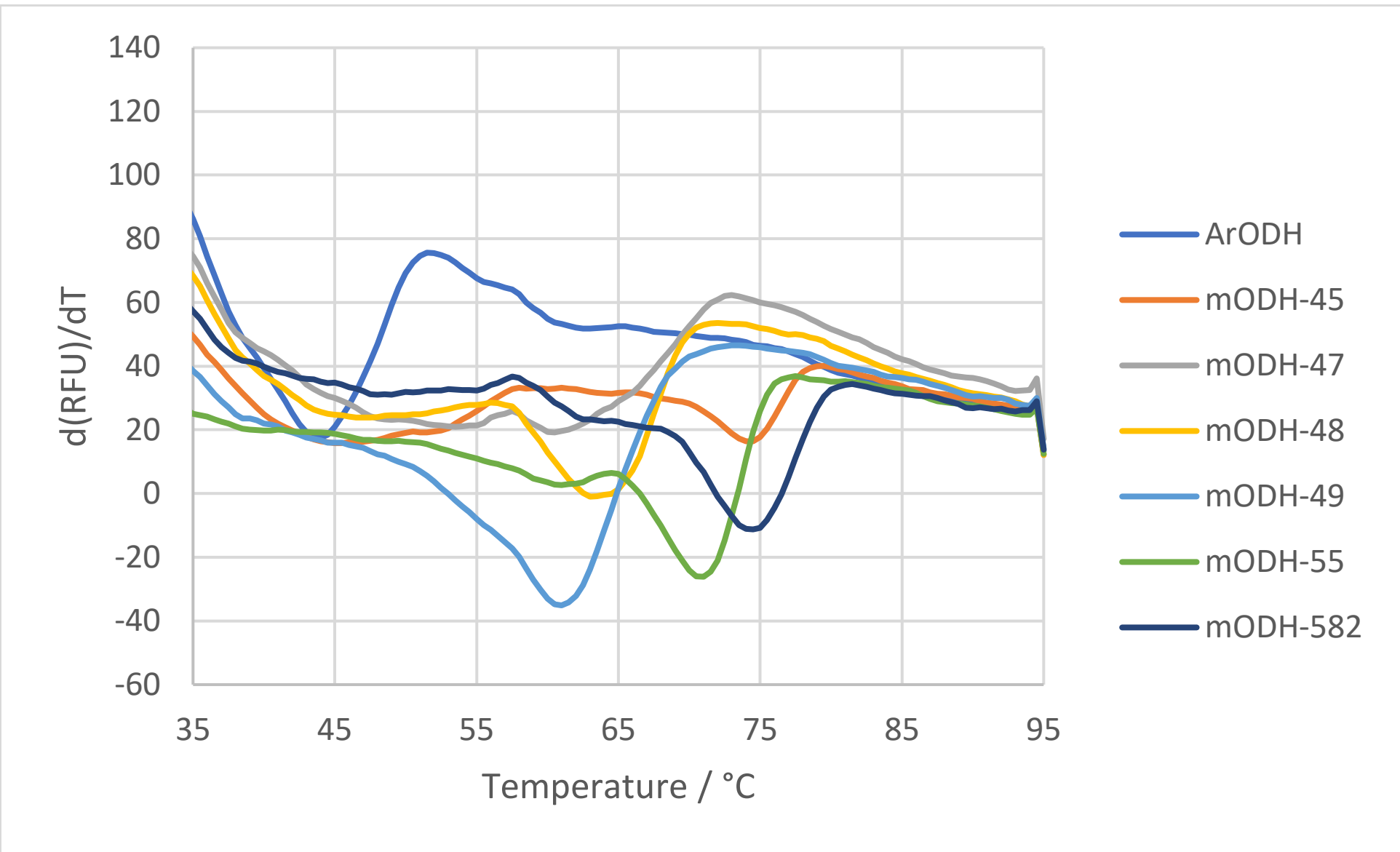

Figure S2 – Melting curves of ODHs measured by differential scanning fluorimetry.

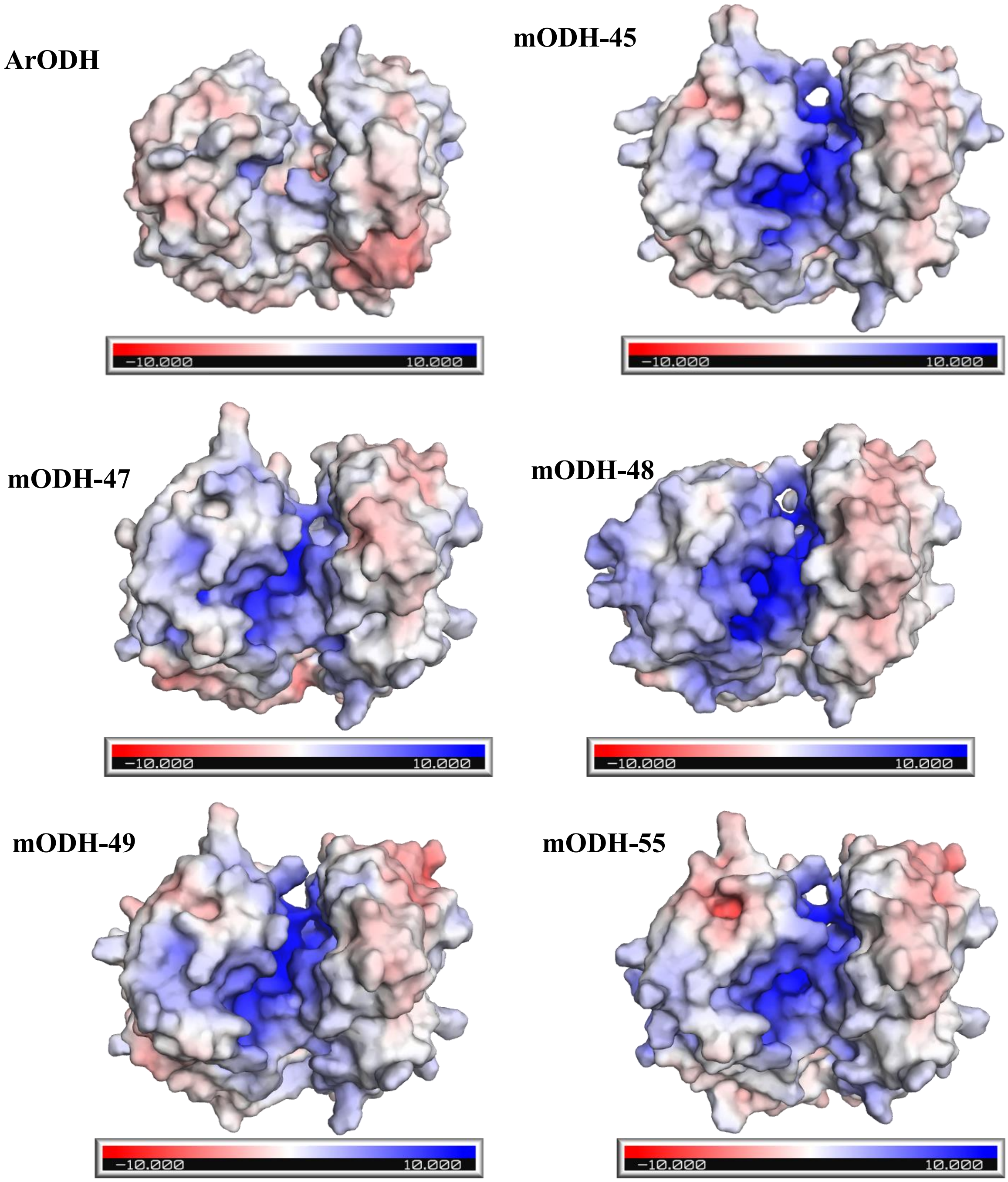

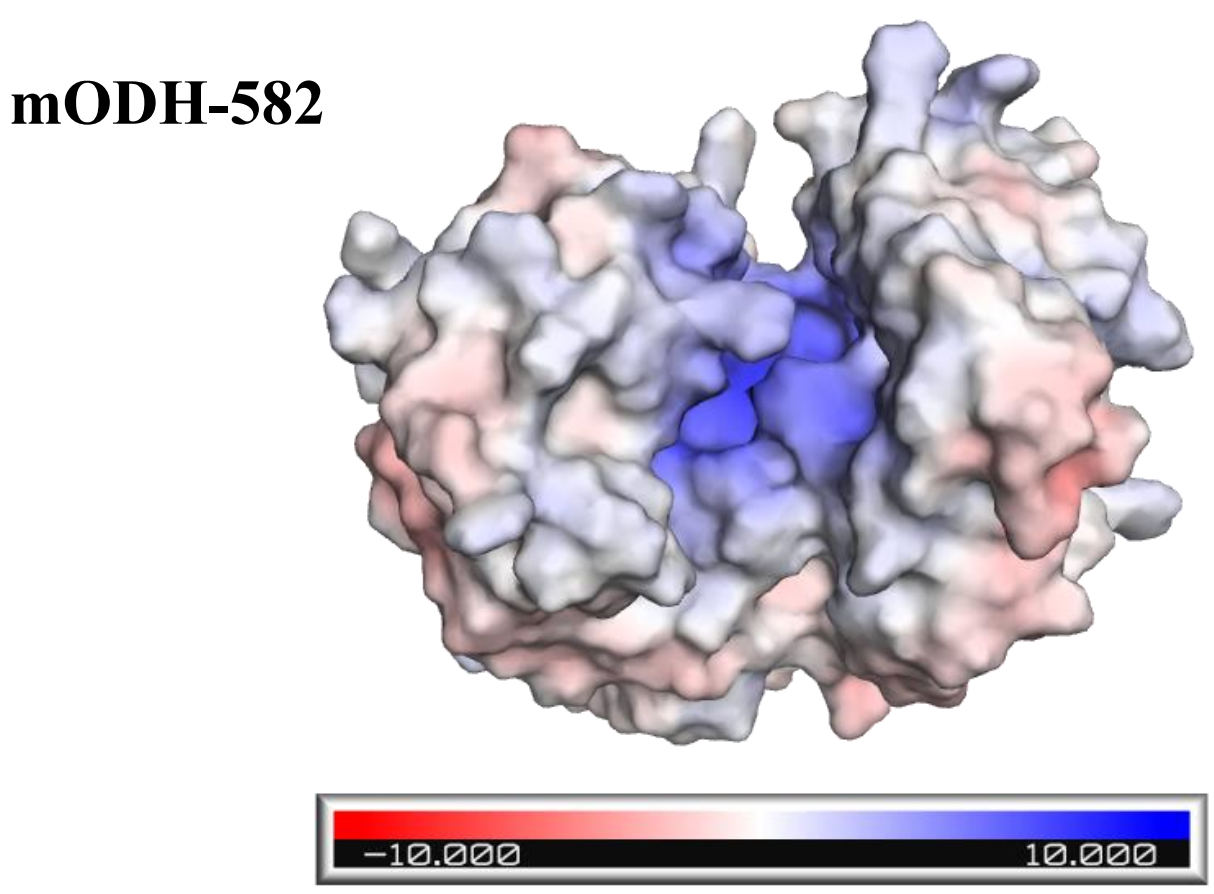

Figure S3 – Surface potential of ArODH and all mODHs
Surface potential is given between -10 and 10 in units K_bT/E_c, where K_b is the Boltzmann constant, T is the temperature and E_c is the charge of an electron.

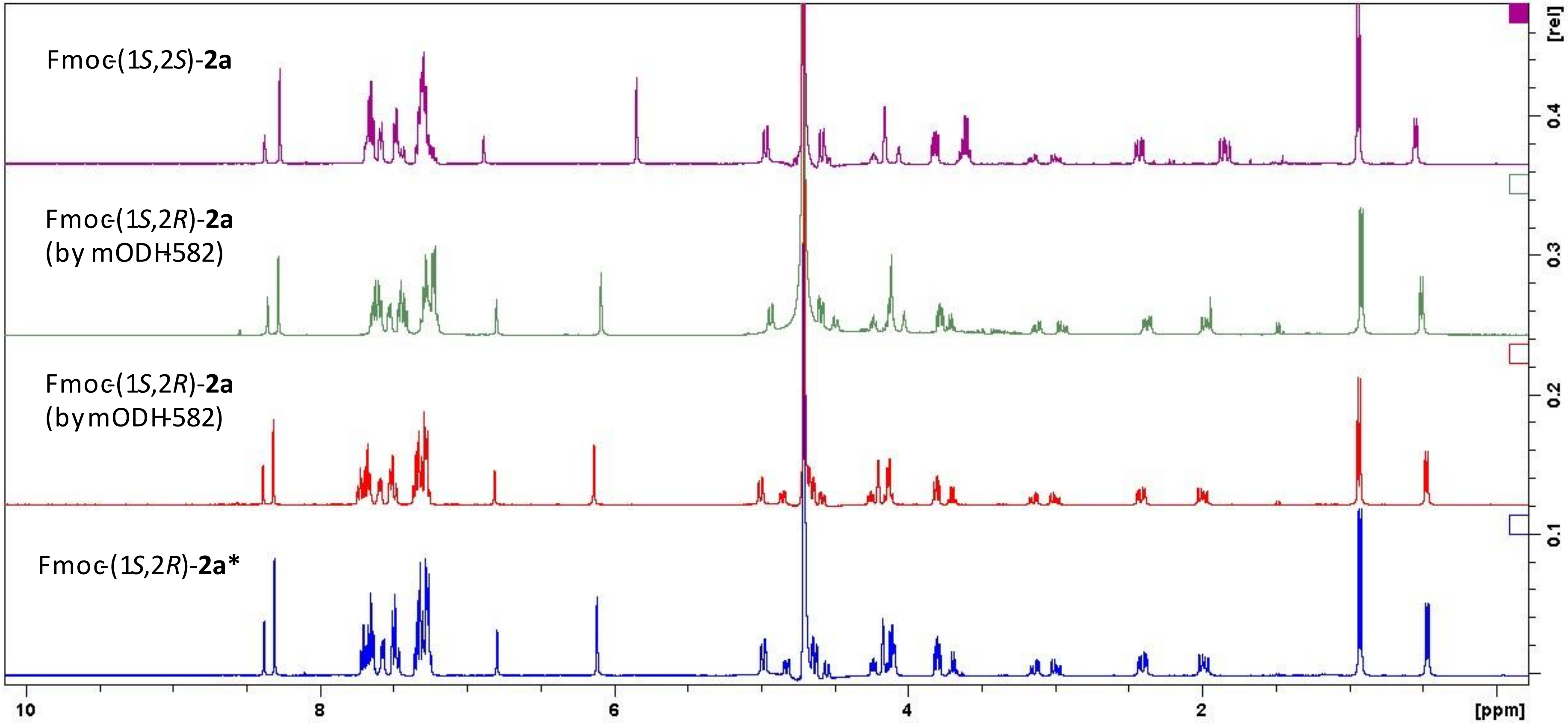

Figure S4 – Comparison of NMR spectra of enzymatic products with chemically synthesized reference compounds

Figure S5 – Comparison of HPLC/UV chromatograms of enzymatic products with chemically synthesized

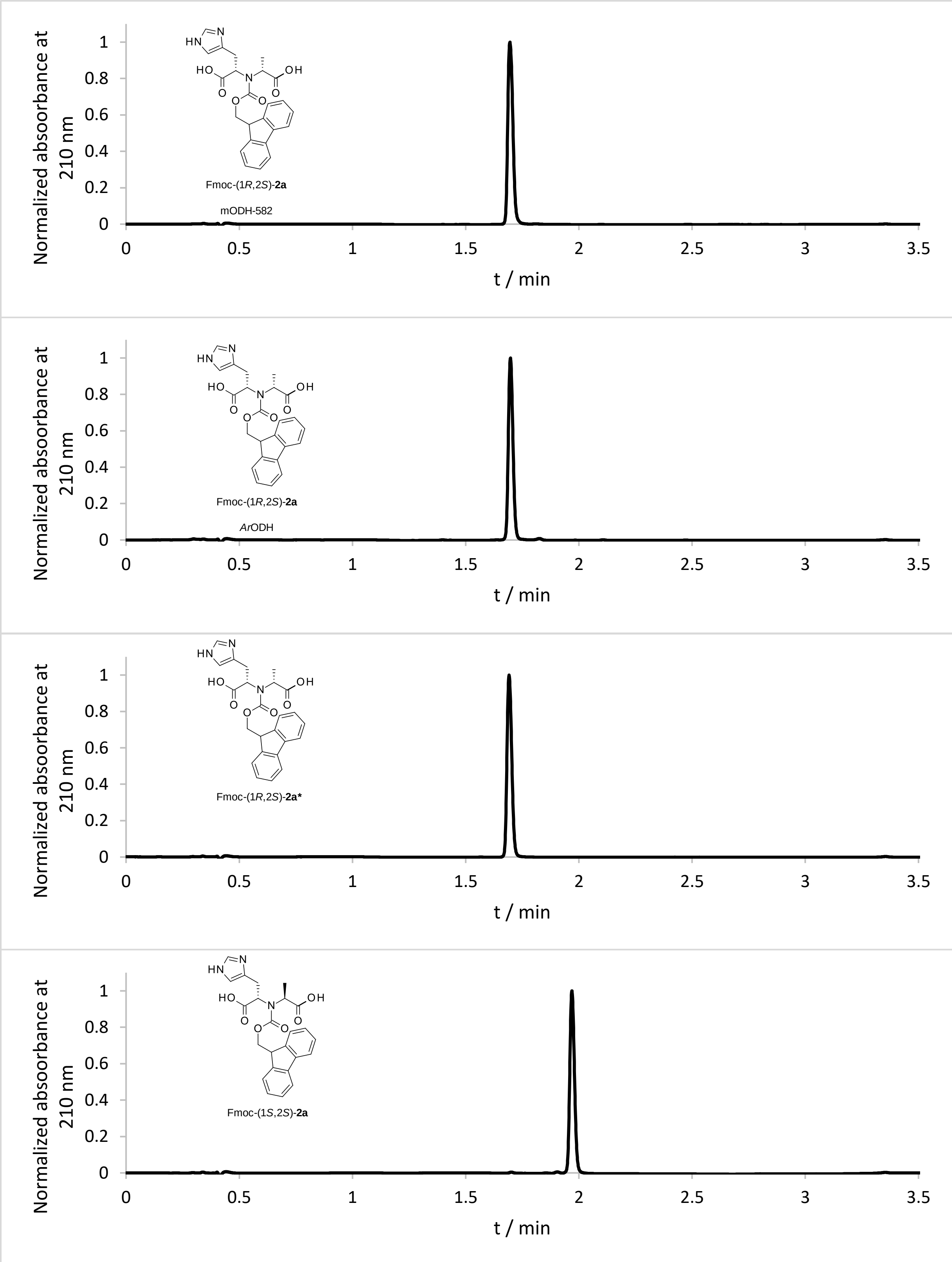

reference compounds

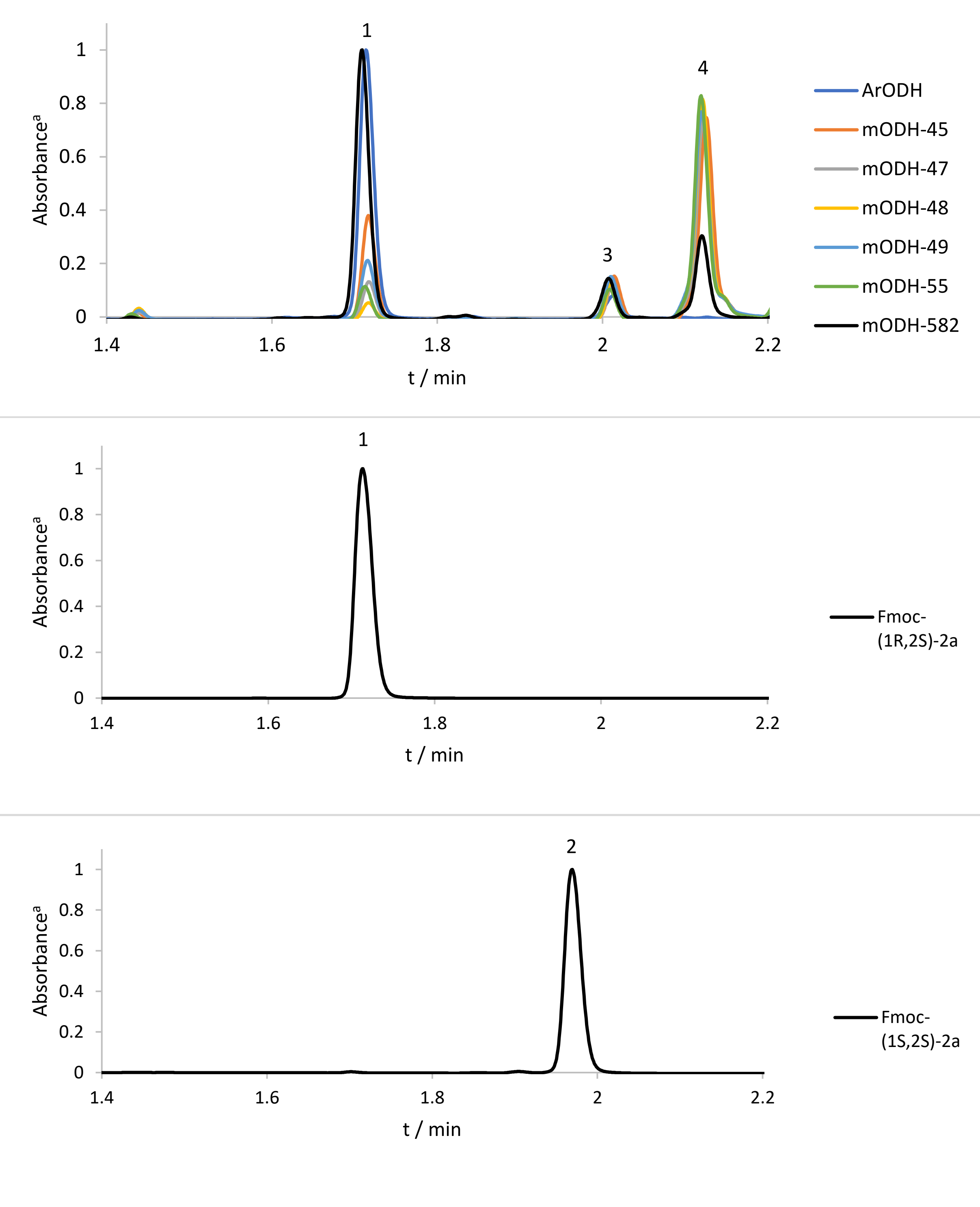

Figure S6 – Comparison of HPLC/UV chromatograms of *Ar*ODH and mODHs in enzymatic reaction with L-histidine (2) and pyruvate (a) with chemically synthesized reference compounds

a: Normalized absorbance at 210 nm; 1: Fmoc-(*1R,2S*)-**2a**; 2: Fmoc-(*1R,2S*)-**2a**; 3: uncharacterized side product (ESI^+ m/z=436) 4: Fmoc-L-histidine

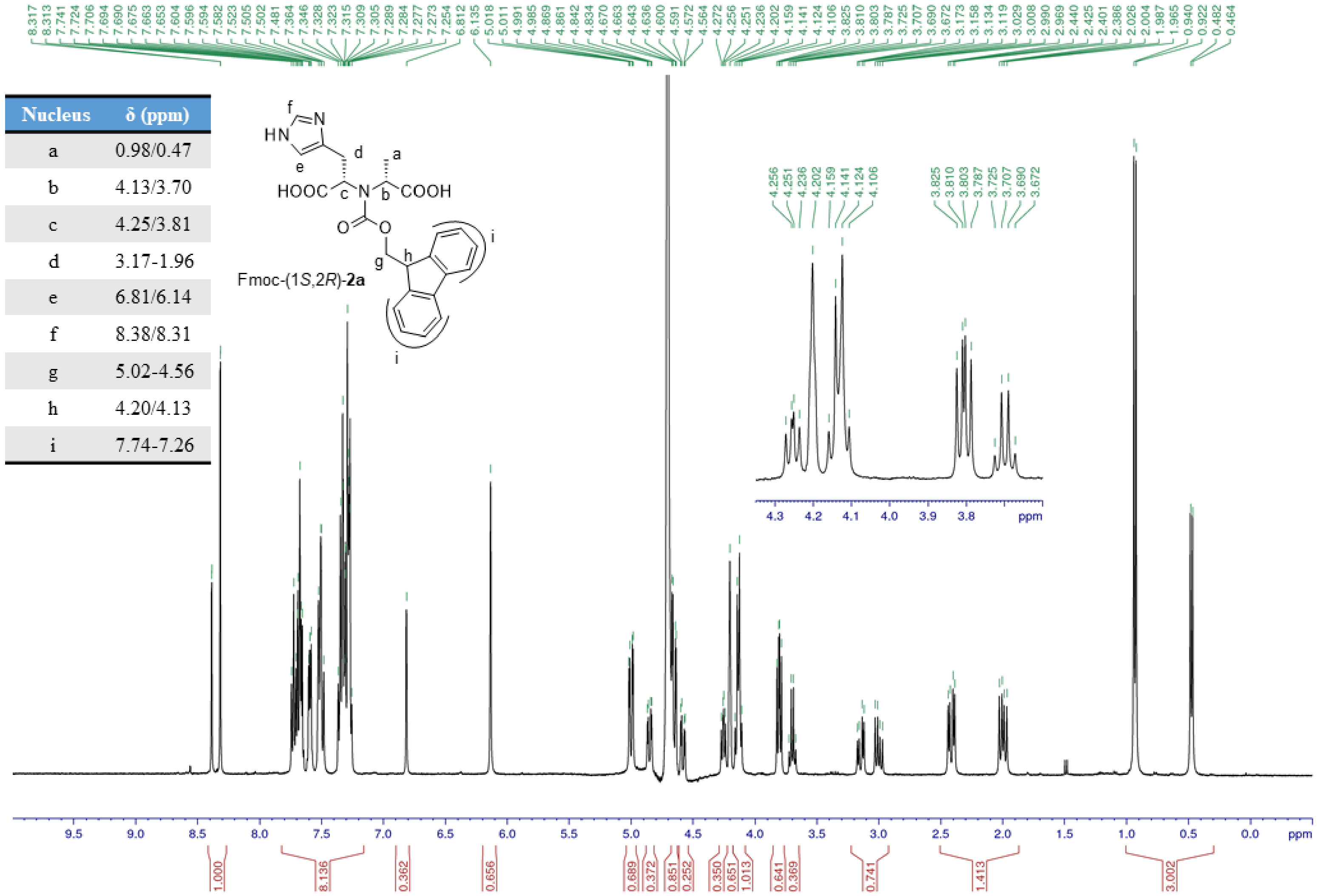

Nucleus	δ (ppm)
a	0.98/0.47
b	4.13/3.70
c	4.25/3.81
d	3.17-1.96
e	6.81/6.14
f	8.38/8.31
g	5.02-4.56
h	4.20/4.13
i	7.74-7.26

Figure S7 - ^{1}H-NMR spectrum of Fmoc-(1*R*,2*S*)-2a (prepared by mODH-582)

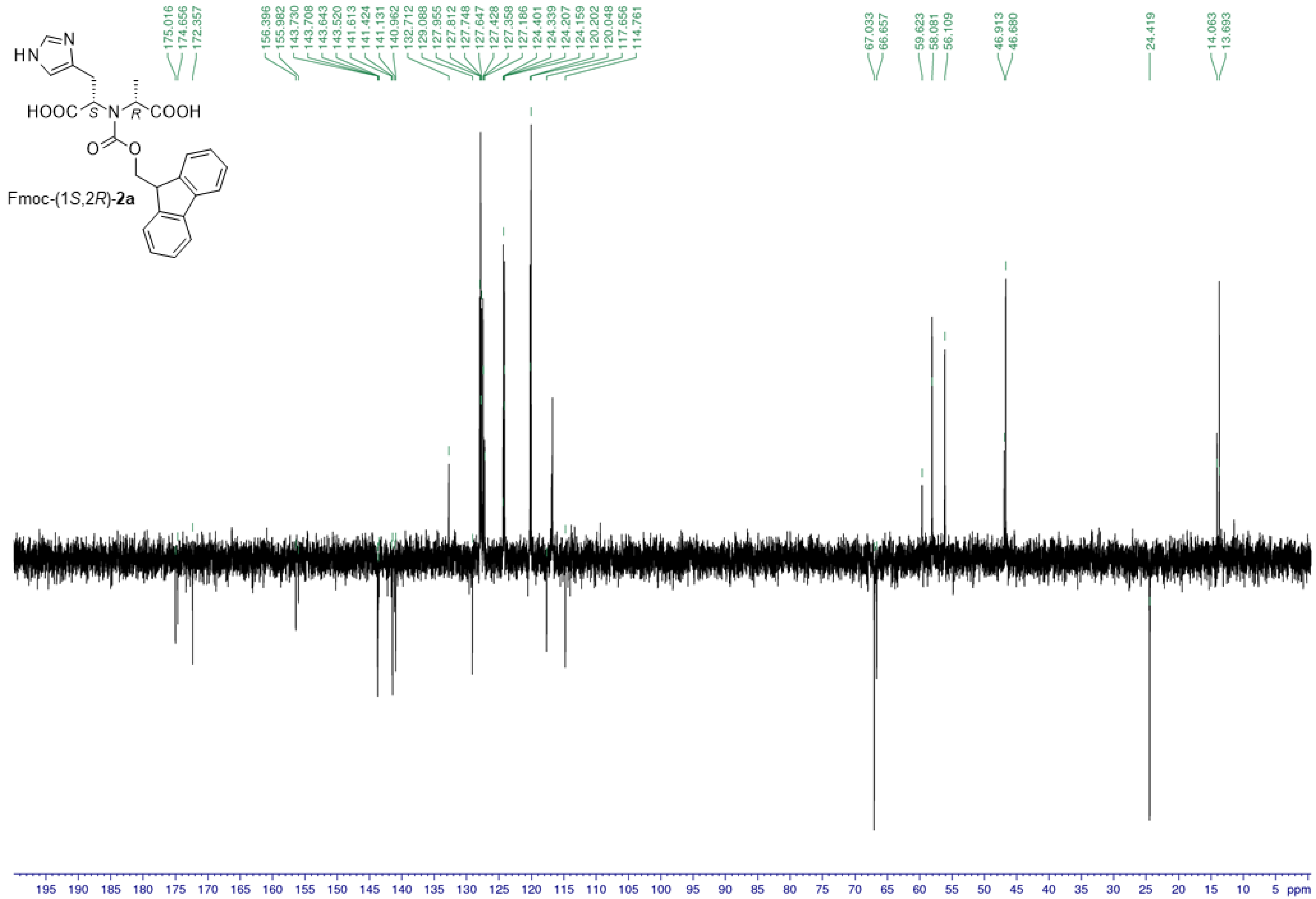

Figure S8 - ^{13}C-NMR spectrum of Fmoc-(1*R*,2*S*)-2a (prepared by mODH-582)

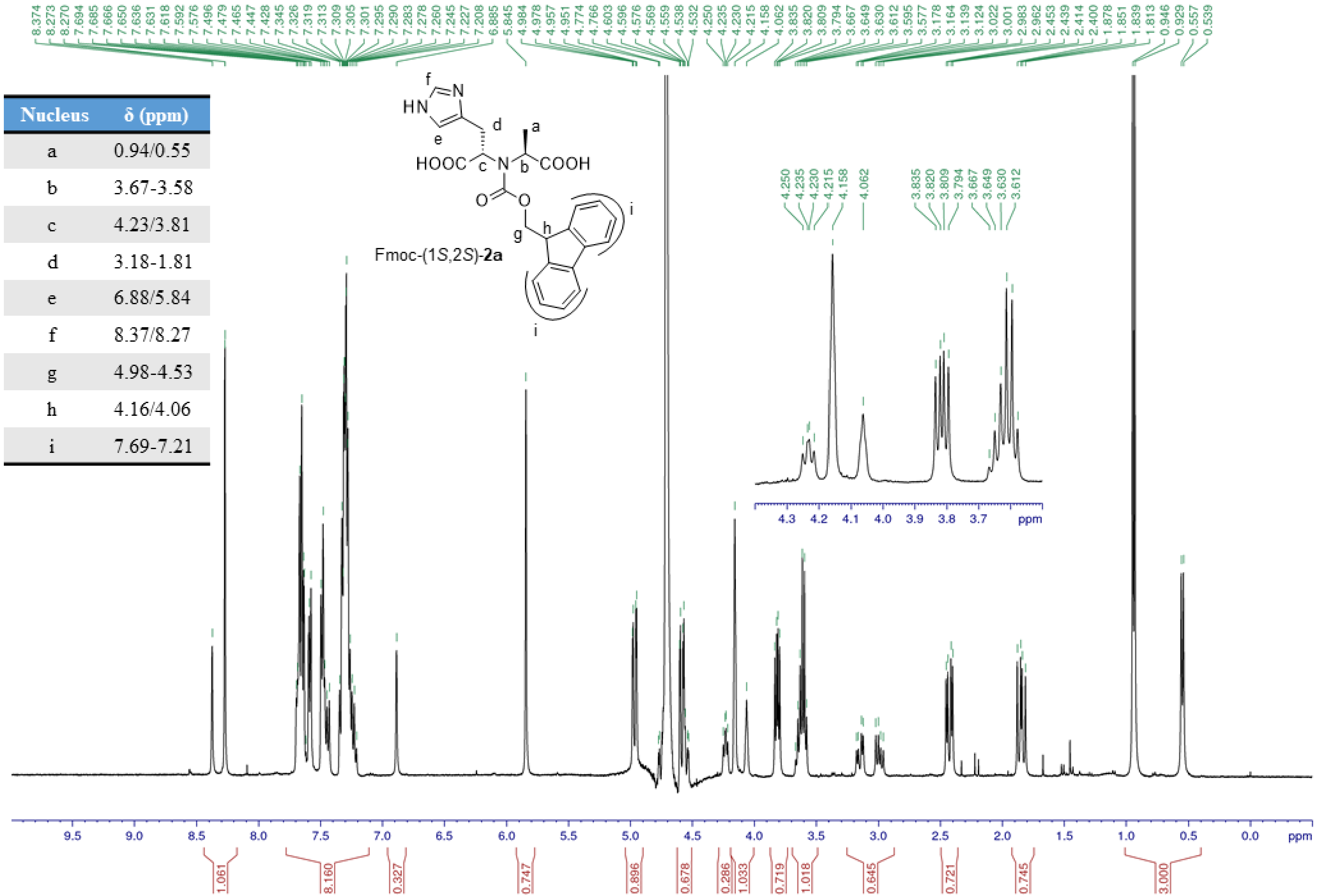

Nucleus	δ (ppm)
a	0.94/0.55
b	3.67-3.58
c	4.23/3.81
d	3.18-1.81
e	6.88/5.84
f	8.37/8.27
g	4.98-4.53
h	4.16/4.06
i	7.69-7.21

Figure S9 - ^{1}H-NMR spectrum of Fmoc-(1*S*,2*S*)-2a

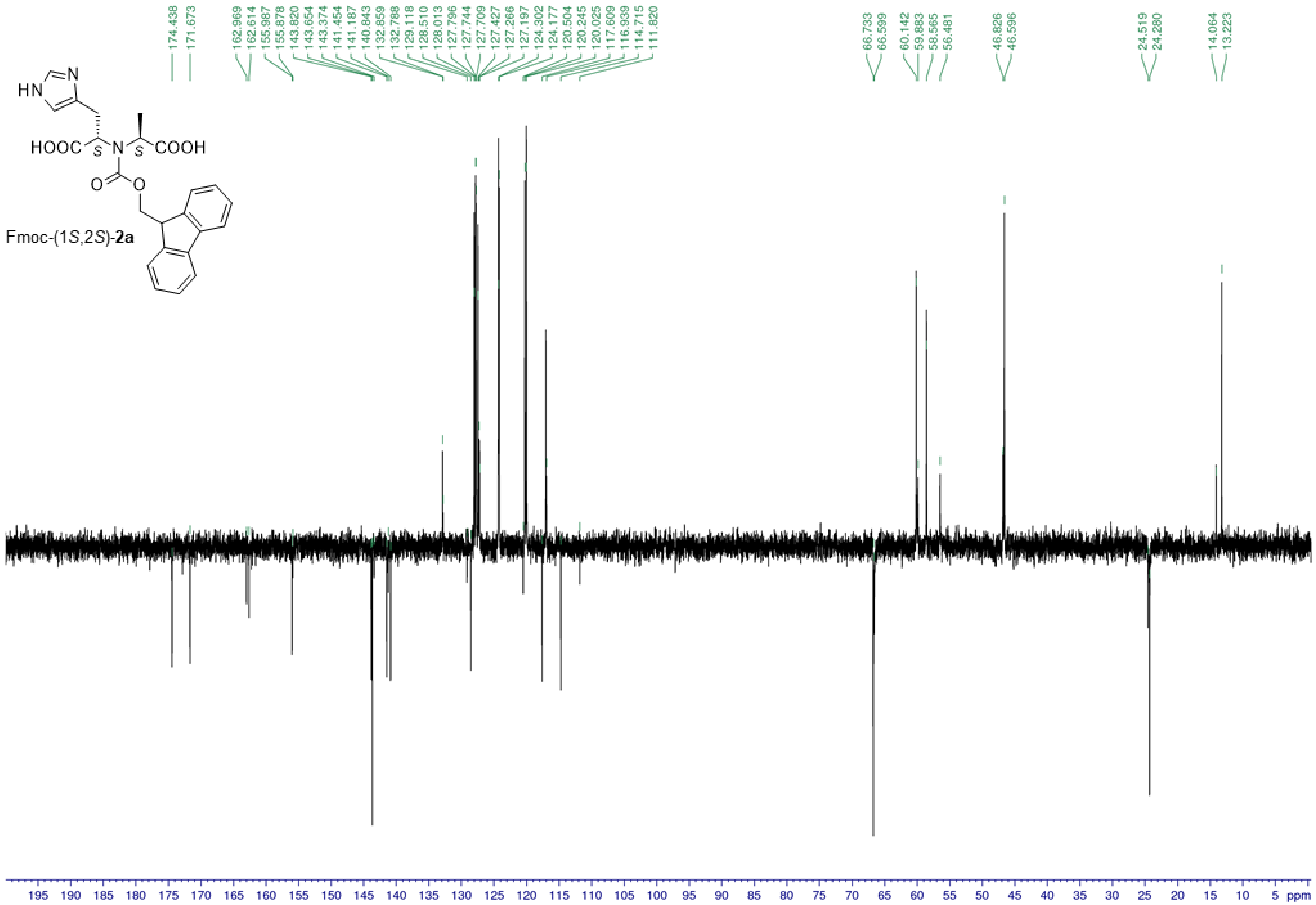

Figure S10 - ^{13}C-NMR spectrum of Fmoc-(1*S*,2*S*)-2a

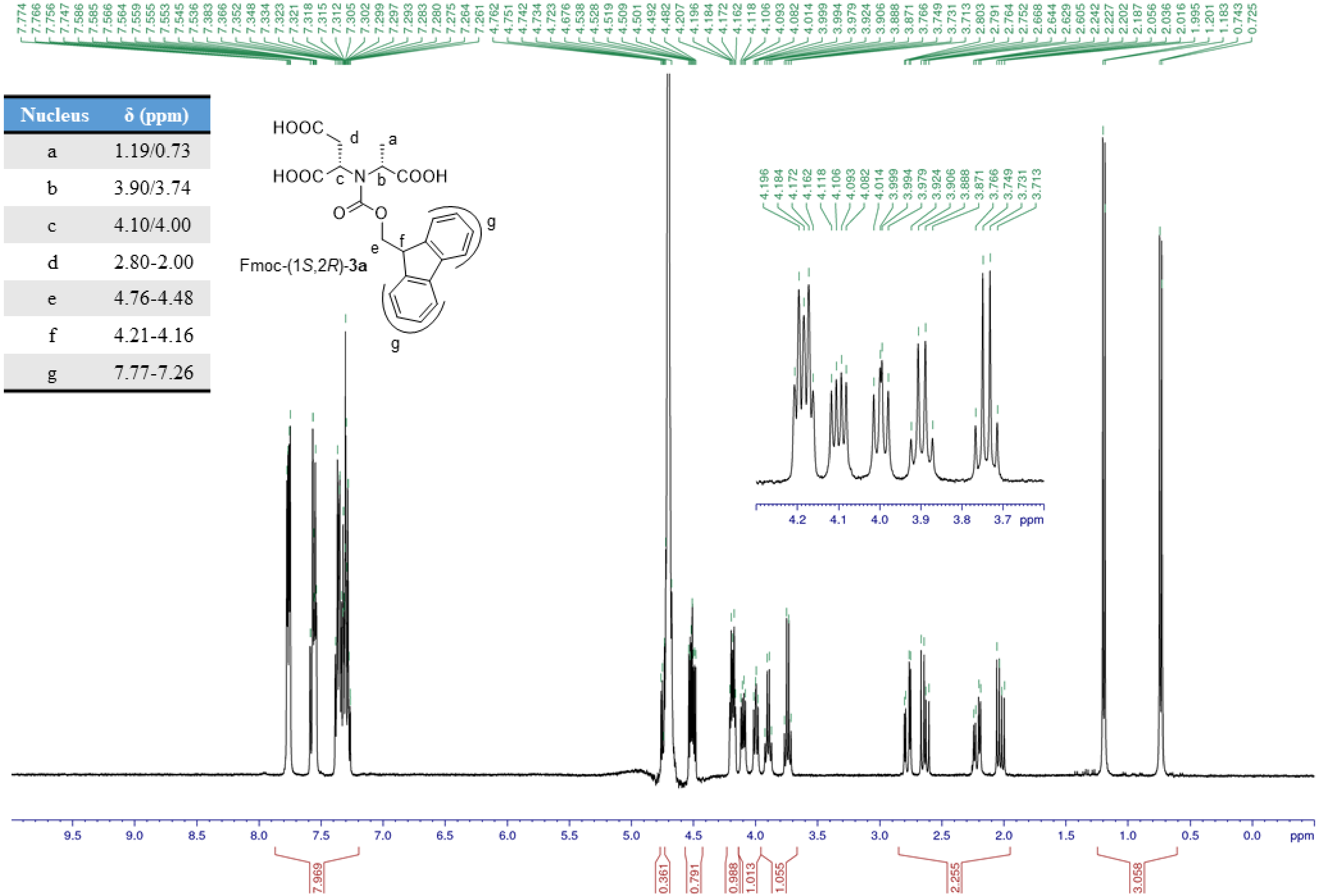

Nucleus	δ (ppm)
a	1.19/0.73
b	3.90/3.74
c	4.10/4.00
d	2.80-2.00
e	4.76-4.48
f	4.21-4.16
g	7.77-7.26

Figure S11 - ^{1}H-NMR spectrum of Fmoc-(1*R*,2*S*)-3a

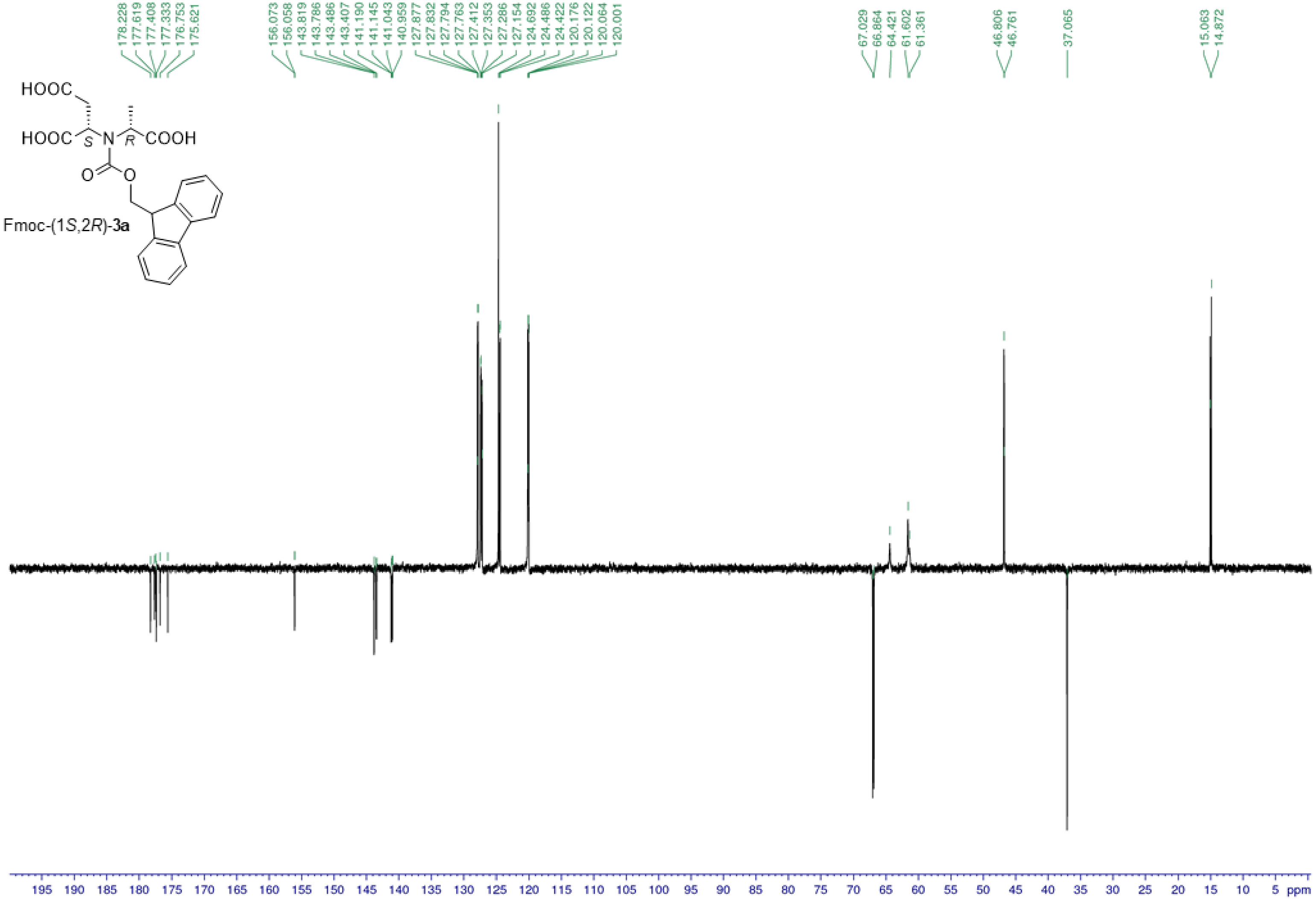

Figure S12 - ^{13}C-NMR spectrum of Fmoc-(1*R*,2*S*)-3a

Nucleus	δ (ppm)
a	1.28/0.83
b	3.81-3.74
c	4.04/3.88
d	3.47-2.13
e	4.83-4.54
f	4.17-4.14
g	7.78-7.26

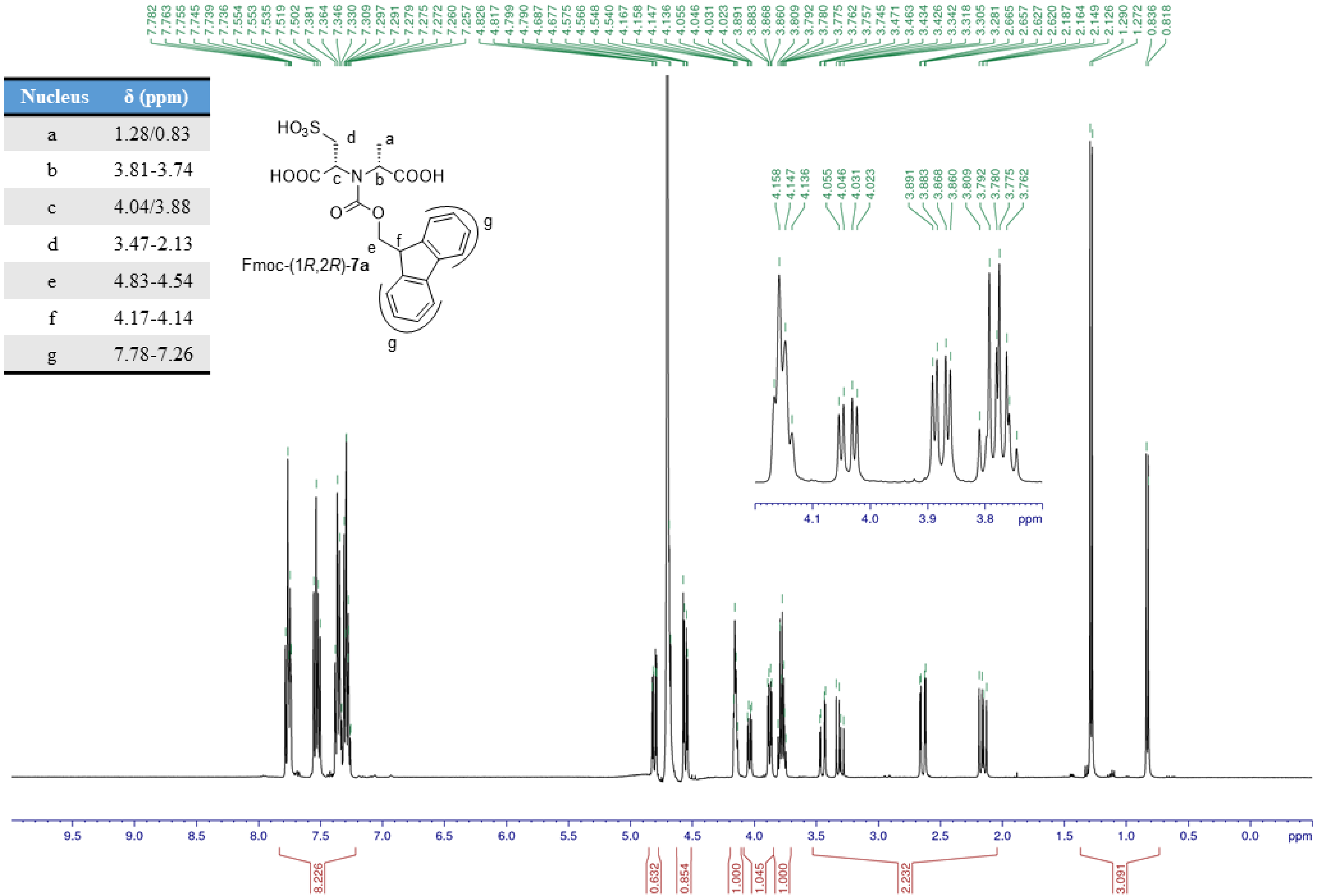

Figure S13 - ^{1}H-NMR spectrum of Fmoc-(1*R*,2*R*)-7a

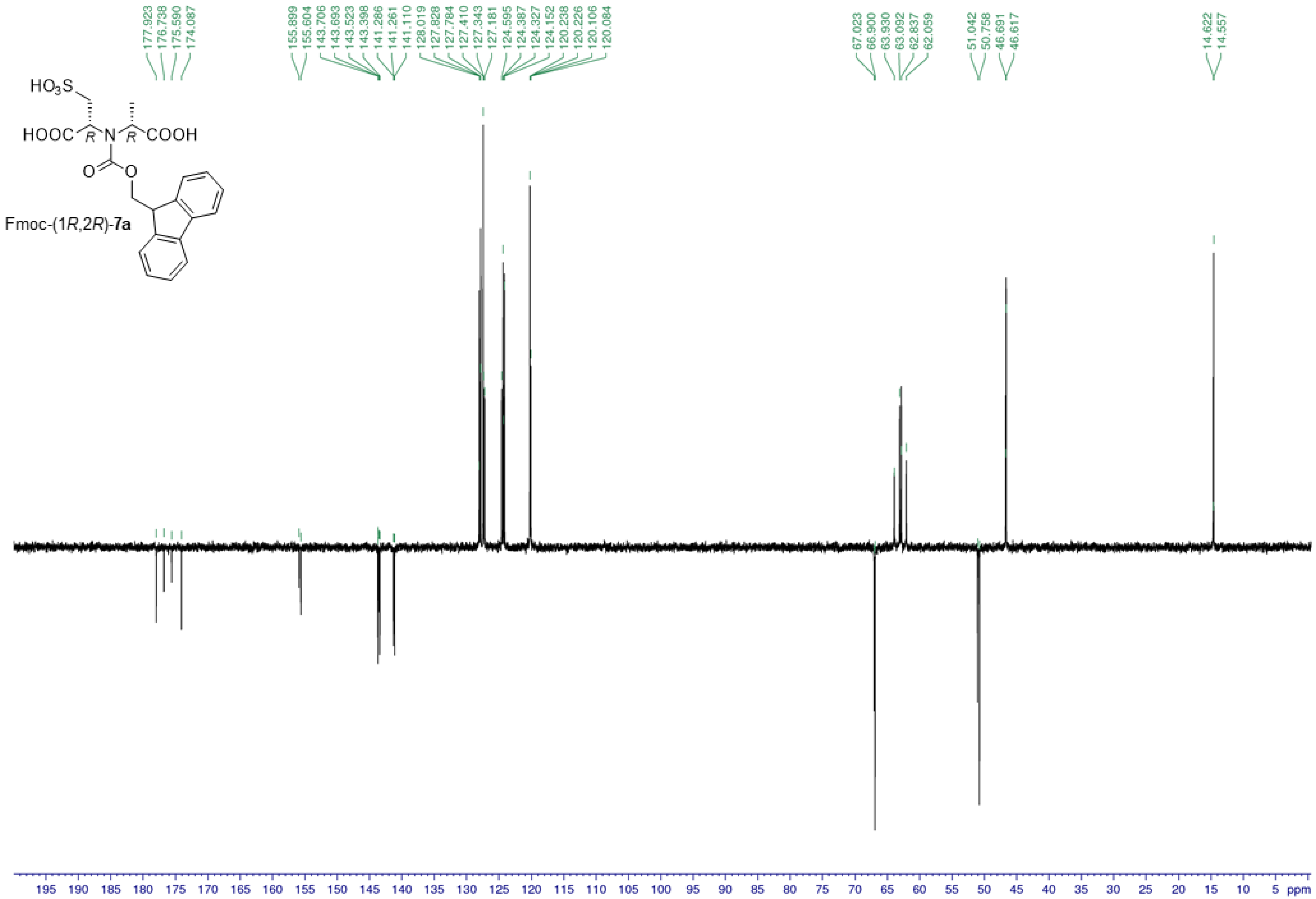

Figure S14 - ^{13}C-NMR spectrum of Fmoc-(1*R*,2*R*)-7a

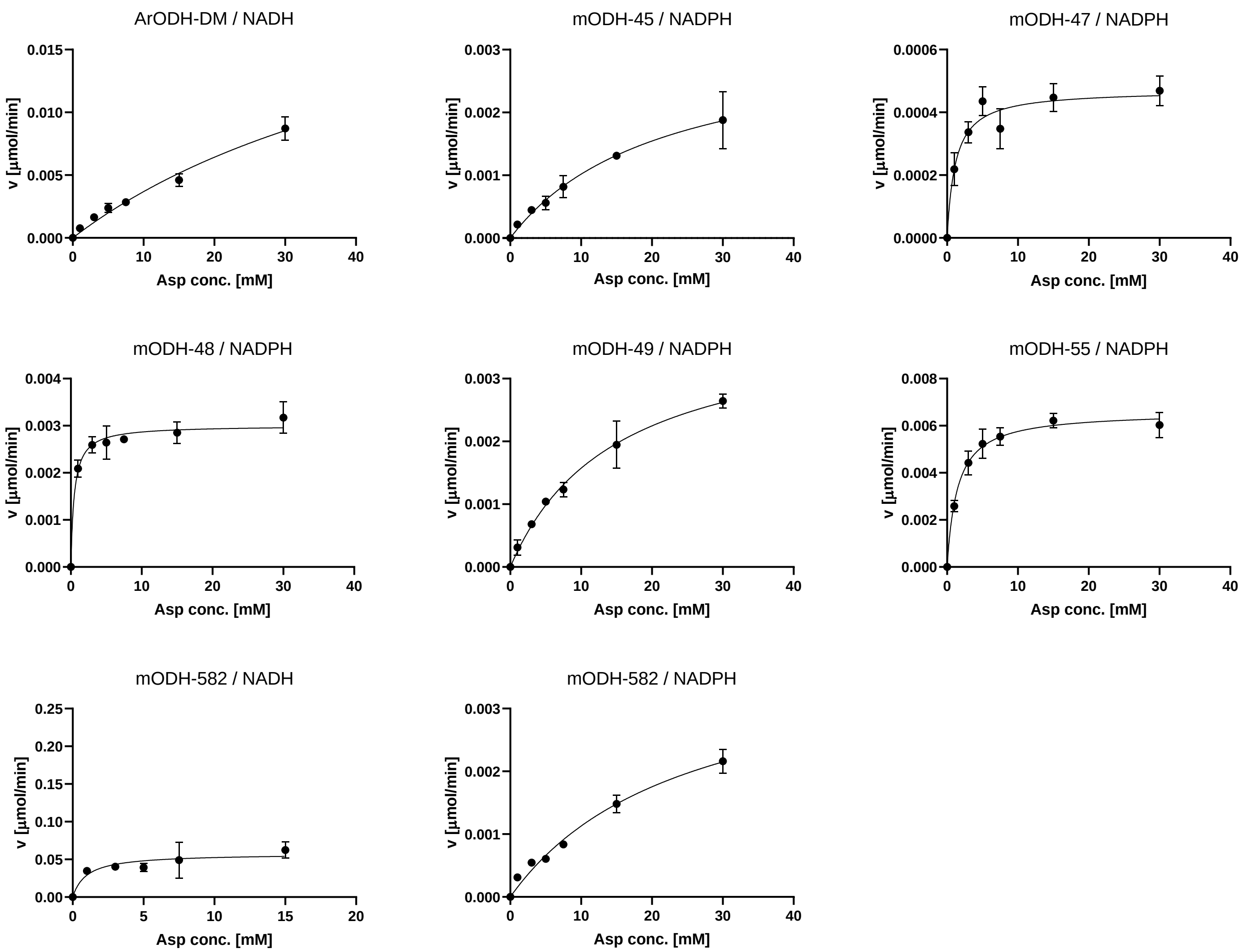

Figure S15 – Measured Michaelis-Menten curves of ODHs for l-aspartate.
mODH-582-DM corresponds to mODH-582 R110A G198N double mutant, while ArODH-DM corresponds to ArODH A111R N198G.

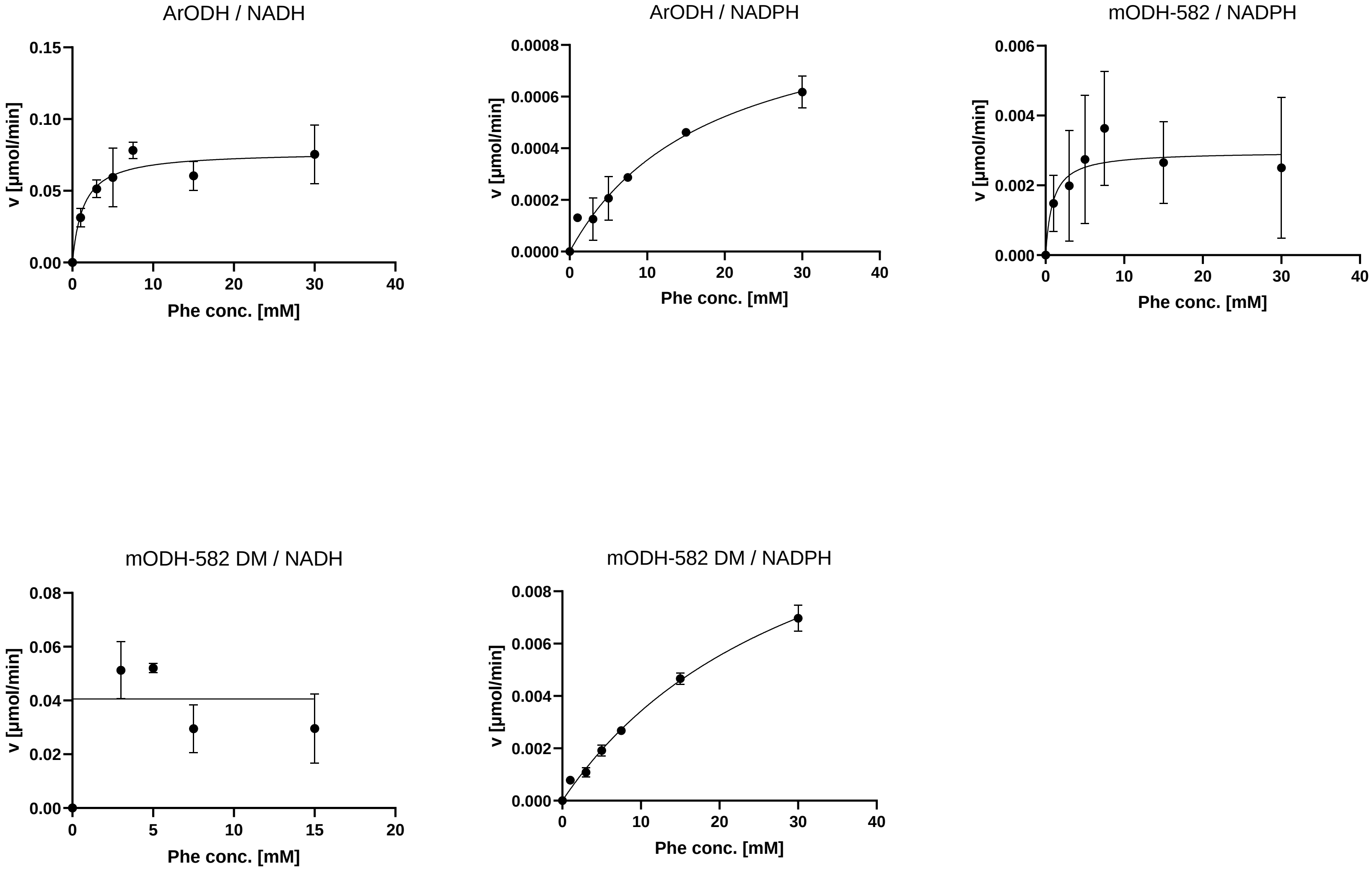

Figure S16 – Measured Michaelis-Menten curves of ODHs for L-phenylalanine.
mODH-582 DM corresponds to mODH-582 R110A G198N double mutant.

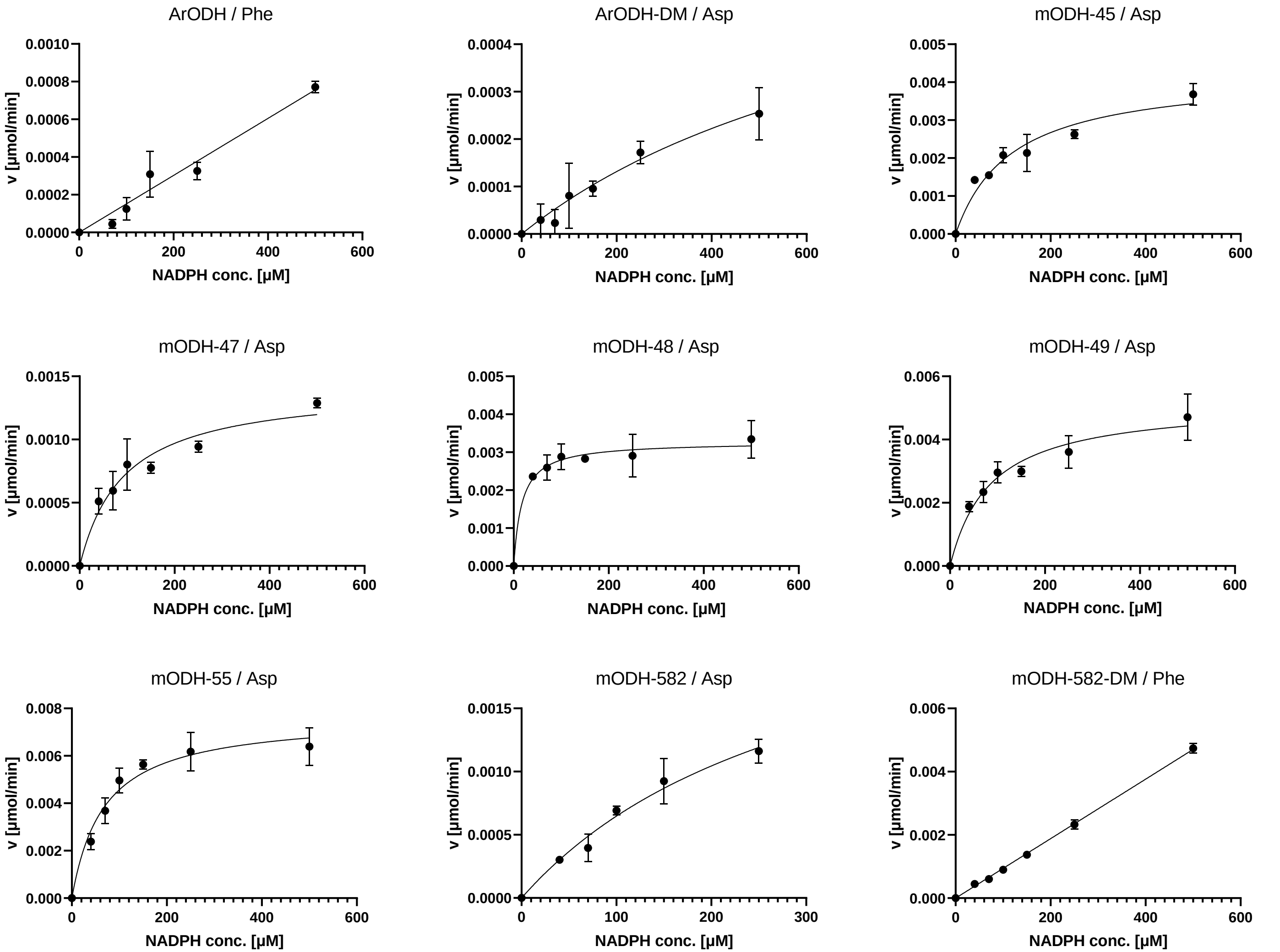

Figure S17 – Measured Michaelis-Menten curves of ODHs for NADPH.
mODH-582-DM corresponds to mODH-582 R110A G198N double mutant, while ArODH-DM corresponds to ArODH A111R N198G.

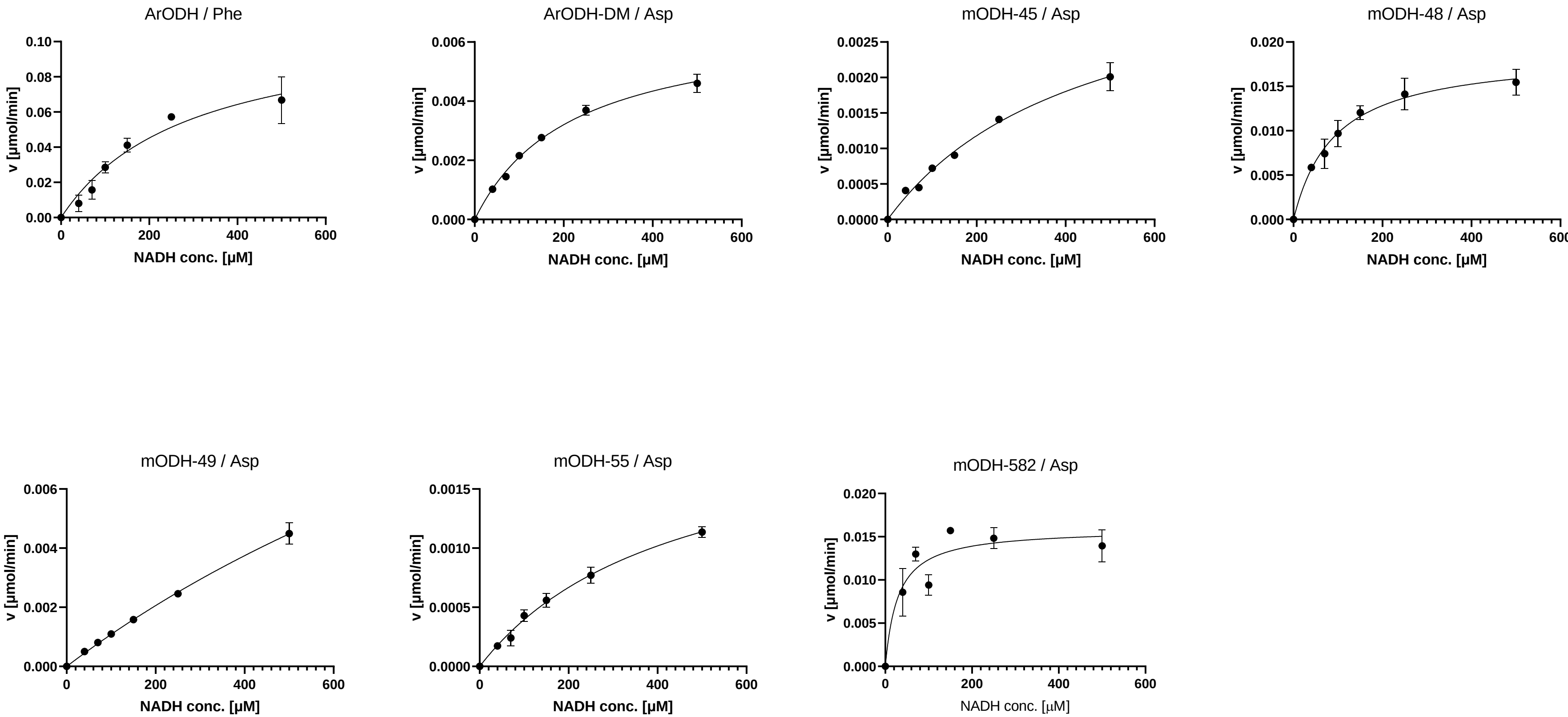

Figure S18 – Measured Michaelis-Menten curves of ODHs for NADH.
ArODH-DM corresponds to ArODH A111R N198G.
